# KLF6 activation marks an angiogenic and apoptosis resistant endothelial phenotype in pulmonary arterial hypertension

**DOI:** 10.1038/s42003-026-10493-5

**Published:** 2026-06-15

**Authors:** Rehab Alharbi, Merve Keles, Nadia Fernandes, Hannah Maude, Adam Fellows, Richard D. Williams, Chien-Nien Chen, Nathalie Lambie, Nik Matthews, May Al-Sahaf, Sam N. Barnett, Minzhe Guo, Lan Zhao, Allan Lawrie, Jeffrey A. Whitsett, Inês Cebola, Beata Wojciak-Stothard

**Affiliations:** 1 National Heart and Lung Institute, Imperial College London, London, UK; 2 Clinical Laboratory Sciences Department, College of Applied Medical Sciences, University of Hafr Al Batin, Hafr Al Batin, Saudi Arabia; 3 Section of Genetics and Genomics, Department of Metabolism, Digestion and Reproduction, Imperial College London, London, UK; 4 Department of Thoracic Surgery, Hammersmith Hospital, London, UK; 5 The Perinatal Institute and Section of Neonatology, Perinatal and Pulmonary Biology, Cincinnati Children’s Hospital Medical Center, Cincinnati, OH USA; 6 Department of Pediatrics, University of Cincinnati College of Medicine, Cincinnati, OH USA

**Keywords:** Mechanisms of disease, Preclinical research, Translational research, Cellular signalling networks

## Abstract

Pulmonary arterial hypertension (PAH) is a severe, currently incurable lung disease characterized by endothelial injury and excessive repair, leading to arterial narrowing. However, the contributory mechanisms remain poorly understood. Here we show that Krüppel-like factor 6 (KLF6) is a feature of vascular pathology in PAH. KLF6 expression is elevated in human PAH and preclinical models of PAH and promotes endothelial repair and angiogenesis through transcriptomic remodelling, with effects distinct from those of KLF2 and KLF4. Endothelial KLF6 also stimulates vascular smooth muscle cell proliferation, which is attenuated by bosentan and imatinib. DisGeNET and spatial transcriptomic analyses of control and PAH lungs reveal elevated KLF6 in PAH endothelium, endothelial progenitor cells, and PAH with alveolar capillary dysplasia. In summary, KLF6 activation uniquely orchestrates endothelial repair and is a feature of the angio-proliferative vascular phenotype in PAH.

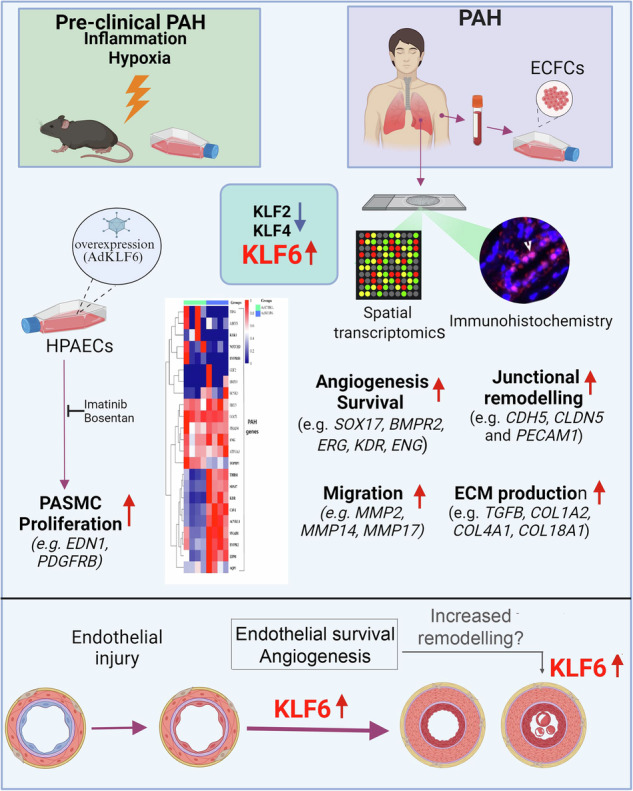

## Abstract (web summary)

KLF6 is shown to be a potential driver of the pulmonary endothelial transcriptional response to hypertension-related stressors and to contribute of plexogenic arteriopathy in pulmonary artery hypertension.

## Introduction

Pulmonary arterial hypertension (PAH) is a progressive and life-limiting vascular lung disease with no cure, despite targeted treatment with multiple therapies^1^. Pathologically, PAH is characterized by sustained pulmonary vasoconstriction and progressive obliteration of resistance pulmonary arteries and arterioles due to medial thickening, intimal fibrosis, and formation of angioproliferative (plexiform) lesions^2^. These changes result in the loss of small distal vessels, which is often described as “vascular pruning”^3^.

PAH is a multifactorial condition where genetic predisposition^4^, hypoxia^5^, and inflammation^6^ play contributory roles. Loss-of-function mutations in bone morphogenetic protein receptor 2 (*BMPR2*) and other genes, mostly from the TGF-β superfamily, have been implicated in PAH^7^.

In the process of PAH development, endothelial damage caused by genetic, epigenetic or environmental factors triggers exaggerated repair, during which clonal expansion of apoptosis-resistant endothelial cells gives rise to complex angio-proliferative (plexiform) lesions^8,9^. Loss of endothelial barrier function, pro-inflammatory and pro-thrombotic activation, endothelial-to-mesenchymal transition (EMT), as well as the proliferation and migration of pulmonary arterial smooth muscle cells (PASMCs) and adventitial fibroblasts drive pulmonary vascular remodelling^2^. The mechanisms that initiate the formation of plexiform lesions are not known, but a contribution of circulating endothelial progenitor cells^10^ or bronchopulmonary anastomoses^11^ have been suggested.

Loss of endothelial homoeostasis in pulmonary arterial hypertension (PAH) has been linked to reduced activity of the Krüppel-like transcription factors KLF2 and KLF4^12,13^. Another member of this family of transcription factors expressed in endothelium, KLF6 has been shown to enhance TGF-β signalling and promote neointima formation following arterial injury^14,15^, but its role in PAH or its relationship with the structurally and functionally related transcription factors KLF2 and KLF4 has not been investigated.

We hypothesized that KLF6 activation is a feature of vascular pathology in PAH. Accordingly, we investigated the effects of PAH-associated hypoxic and inflammatory stressors on KLF6 expression in vitro and in vivo and defined the functional and transcriptional consequences of KLF6 activation in the pulmonary vascular endothelium. We show that KLF6 activation promotes angiogenesis and reduces endothelial susceptibility to apoptosis in vitro and is a hallmark of an angio-proliferative endothelial phenotype in plexogenic arteriopathy in PAH. Furthermore, we identify similarities and differences in the activation patterns and transcriptional programmes regulated by KLF2, KLF4, and KLF6 in lung endothelial cells, highlighting the specific role of KLF6 in promoting vascular angiogenesis.

## Results

### *KLF6* expression is increased under PAH-related vascular stress conditions

**Fig. 1:**
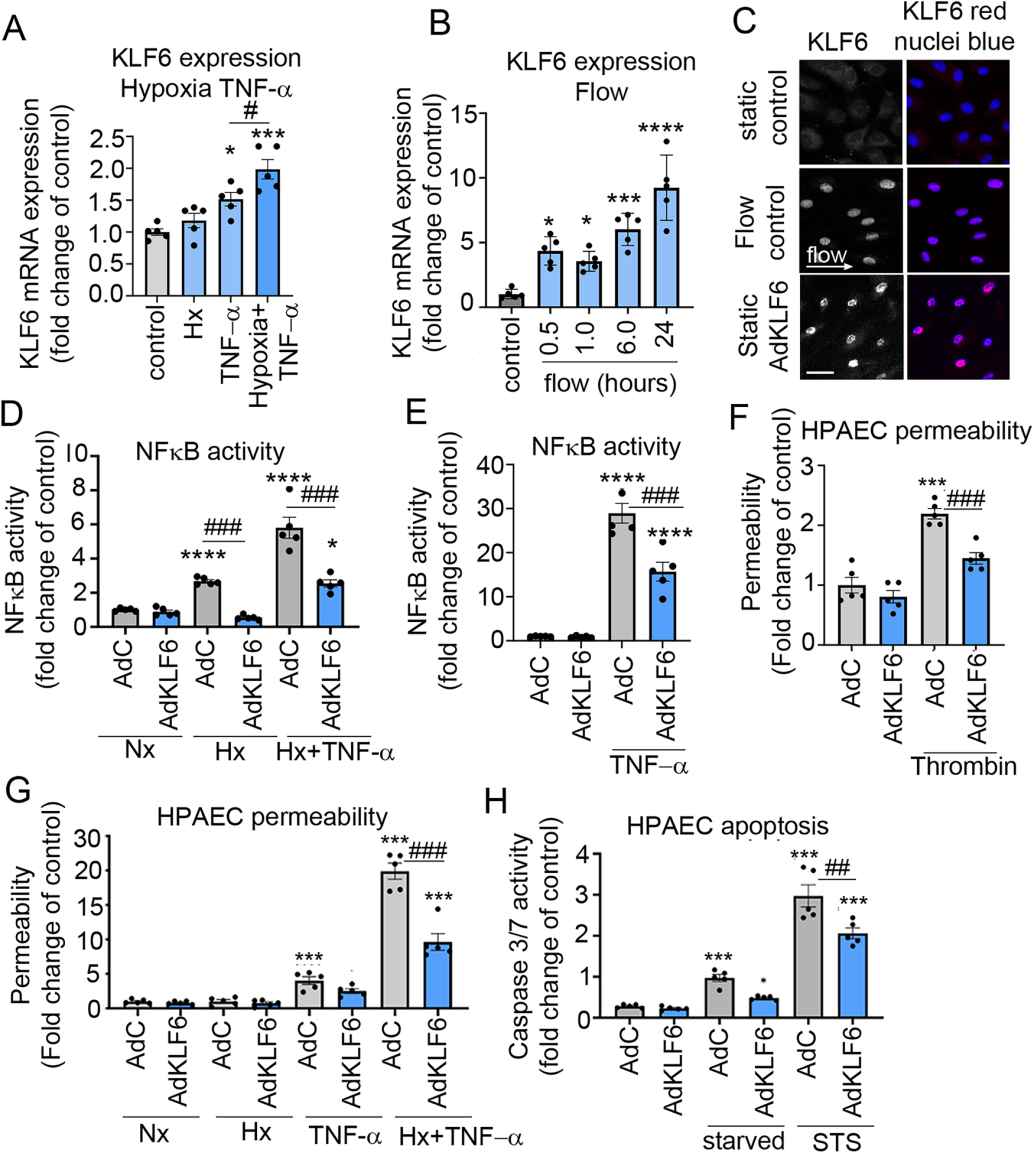
KLF6 in endothelial responses to PAH-associated stress conditions. **A** KLF6 mRNA expression in HPAECs exposed to hypoxia (2% O_2,_ 24 h) with, or without TNF-α (10 ng/ml, 24 h) or **B** in HPAECs exposed to flow (4 dynes/cm^2^ 0–24 h). *n* = 5. **C** Localization of KLF6 in HPAECs under flow or HPAECs infected with AdKLF6. In merged images, nuclei are blue and KLF6 is red, confocal microscopy. Scale bar = 10 μm. Panels** D**–**F** show effects of KLF6 overexpression on NF-κB activity in HPAECs treated with TNF-α (10 ng/ml, 24 h), hypoxia (2% O2, 24 h) or thrombin (1 U/mL, 1 h), as indicated. AdCTRL: adenoviral control; AdKLF6: adenoviral KLF6. **G** Effect of KLF6 overexpression on endothelial permeability in hypoxia (Hx) and TNF-a-treated HPAECs, as indicated. **H** Effect of KLF6 on starvation- and staurosporine (STS, 01 µM, 24 h)-induced endothelial apoptosis. In all graphs, bars show mean fold-changes of control ± SEM. KLF6 expression was normalised to B2M. *P < 0.05, ***P < 0.001, ****P < 0.0001, comparisons with untreated or viral controls, as appropriate. #*P* < 0.05, ##*P* < 0.01, ###*P* < 0.001, comparison with corresponding treatment controls, as indicated. One-way ANOVA with Tukey’s post hoc test; In (**A–H**) *n* = 5 independent experiments. Source data for graphs are provided in Supplementary data 12.

To mimic the PAH-associated vascular stress conditions in vitro, HPAECs were exposed to hypoxia (2% O₂) and TNF-α, either separately or in combination. The combined treatment was performed to establish the “double hit” conditions, known to induce a more severe vascular phenotype in experimental PAH^16,17^. When combined, hypoxia and TNF-α induced a more pronounced increase in *KLF6* expression in HPAECs than either factor alone (Fig. 1A). Notably, while *KLF6* expression was upregulated, the expressions of *KLF2* and *KLF4* were reduced (Supplementary Fig. 1A, B).

Flow exposure increased *KLF2, KLF4*, and *KLF6* expression in HPAECs, with peak expression of *KLF6* observed at later time points than *KLF2* and *KLF4* (Fig. 1B and Supplementary Fig. 1C, D).

Altogether these analyses reveal that hypoxic, inflammatory and mechanical stress increase *KLF6* expression in cultured pulmonary endothelial cells and follows a distinct activation pattern from *KLF2* and *KLF4*.

### KLF6 improves endothelial survival and stimulates angiogenesis

To investigate the effect of KLF6 activation in the pulmonary endothelium, KLF6 was overexpressed in HPAECs via adenoviral gene transfer. KLF6 exhibited nuclear localization, both when exogenously over-expressed and when induced by the onset of flow (Fig. 1C and Supplemental Fig. 1E). The viral transduction was optimised to induce a ~ 6-fold increase in KLF6 protein (Supplemental Fig. 1F), comparable with changes seen during placental differentiation^18^ and endothelial injury^15^.

KLF6 overexpression enhanced endothelial resilience and survival by suppressing hypoxia- and TNF-α-induced pro-inflammatory NFκB activation in HPAECs (Fig. 1D, E), improving endothelial barrier function (Fig. 1F, G), and reducing apoptosis caused by serum starvation and staurosporine (STS)^19,20^ treatment (Fig. 1H). Conversely, silencing KLF6 produced opposite effects (Supplemental Fig. 2A–D).

**Fig. 2:**
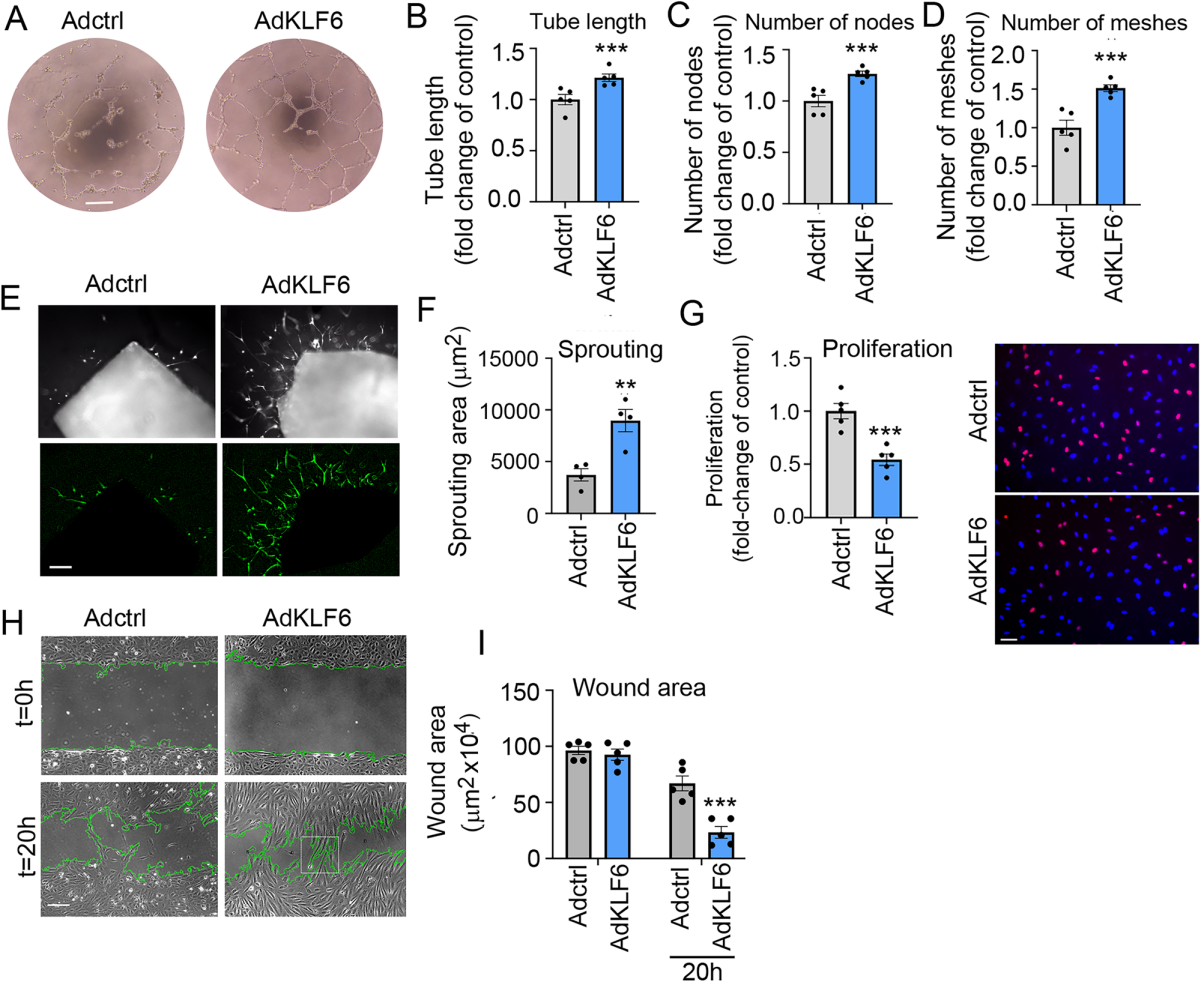
Effects of KLF6 on endothelial angiogenesis, proliferation and migration. **A** Images of tube formation in AdCTRLand AdKLF6-treated HPAECs; phase contrast microscopy. **B** Total tube length, **C** number of nodes and **D** meshes in HPAECs treated, as indicated. ImageJ with Angiogenesis Analyzer plugin. **E** Sprouting angiogenesis in human pulmonary artery explants treated with AdCTRLor AdKLF6. Explants were labelled with Vybrant CFDA SE cell tracker green, fluorescent microscopy. **F** Sprouting area in explants treated, as indicated. *n* = 4 donors; 8 explants/donor were analysed. **G** Graph and corresponding images show HPAEC proliferation; EdU incorporation assay. Images in (**H**) and corresponding graph in (**I**) show endothelial wound healing in control and KLF6-overexpressing HPAECs. Boxed area shows well polarised AdKLF6-overexpressing cells migrating into the wound area. Wound edges are traced in green. In (**B**, **D**, **F**, **G**) **P < 0.01, comparison with adenoviral controls, unpaired *t* test; In (**I**) **P < 0.01, ***P < 0.001, comparison with adenoviral controls, ^###^ *P* < 0.001, comparison, as indicated; two-way ANOVA with Tukey’s post hoc test; *n* = 4–5 independent experiments. Bars show mean ± SEM. In (**A**, **E**, **G**, **H**) scale bar = 50 μm. Source data for graphs are provided in Supplementary data 12.

KLF6 had a profound stimulatory effect on endothelial angiogenesis, as evidenced by increased capillary network formation in KLF6-overexpressing HPAECs (Fig. 2A–D) and increased sprouting of human pulmonary artery explants transduced with AdKLF6 (Fig. 2E, F). In those explants, the adenoviral transduction efficiency of the endothelial cell layer exceeded 80% (Supplementary Fig. 3).

To gain further insight into the underlying mechanisms, we examined the effects of KLF6 on endothelial wound healing in vitro. KLF6 overexpression markedly accelerated endothelial wound closure under both optimal and serum- and growth factor-reduced culture conditions, while exerting an inhibitory effect on HPAEC proliferation (Fig. 2G–I and Supplementary Fig. 4A, B). Conversely, KLF6 silencing produced the opposite effect (Supplementary Fig. 4C, D). Endothelial wound healing in vitro predominantly depends on cell migration^21^ and therefore the effects of KLF6 observed here are most likely to result from enhanced endothelial motility.

In summary, these functional studies indicate that KLF6 is induced by vascular stress to promote angiogenesis and mitigate endothelial damage.

### KLF6-regulated endothelial transcriptome HPAECs under healthy and disease conditions

**Fig. 3:**
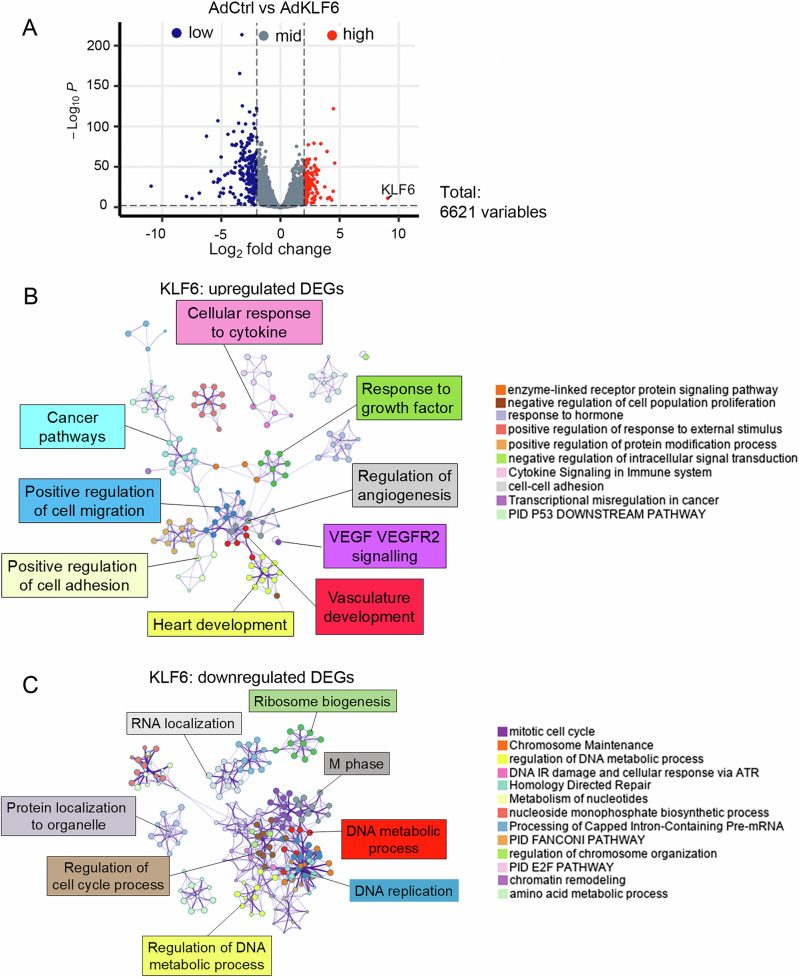
Enrichment analysis of KLF6 DEGs. **A** Volcano plot showing differentially expressed genes (DEGs) in AdKLF6 and AdCTRL treated HPAECs. Downregulated genes are blue and upregulated genes are red. *p* < 0.05 and Log_2_ fold change <–1 or >1. Metascape network of enriched terms in (**B**) upregulated and **C** downregulated DEGs coloured by cluster ID.

KLFs can act as either transcriptional activators or repressors^22^, and the functional assays described above suggest a key role for KLF6 in regulating endothelial gene programmes. Thus, we conducted RNA-seq to characterise the transcriptomic changes induced by KLF6 over-expression in HPAECs (*n* = 4 biological replicates/condition), to identify the set of endothelial genes regulated by KLF6 (Fig. 3A). We detected a total of 4732 differentially expressed genes (DEGs) in HPAECs overexpressing KLF6 (FDR < 0.05, log_2_ | FC | >0.25), including 2332 downregulated and 2400 upregulated genes. This included 204 downregulated and 157 upregulated genes passing a more stringent criteria of FDR < 0.01 and log_2_ | FC | > 2 (Supplementary Data 1). A heatmap with top 50 KLF6-upregulated and downregulated genes is shown in Supplementary Fig. 5.

Metascape^23^ gene set annotation analysis showed that the genes upregulated upon KLF6 overexpression were significantly enriched in terms associated with angiogenesis, cell adhesion and migration, vascular development and VEGF signalling (Fig. 3B). Notably, KLF6 enhanced expression of several key regulators of endothelial repair and angiogenesis, including *ERG*^24^*, ANG-2*^25^*, TIE2*^25^*, BMPR2*^26^*, KDR*^27^*, HES1*^28^, *CDH5*^24^*, PECAM1*^29^, *FLT1*^30^ and the arterio-venous specification gene *SOX17*^31^ (Supplementary Data 1). The downregulated genes were mainly enriched in generic terms, including cell cycle progression, DNA replication and DNA metabolic process (Fig. 3C). The changes in the expression of selected genes were replicated by qPCR in an additional set of HPAECs (Supplementary Fig. 6).

Given the effects we observed on KLF6 expression under hypoxia and TNF-α, two key PAH contributory factors (Fig. 1), we then investigated the impact of KLF6 on the transcriptional response of HPAECs to these two stressors. We started by assessing the transcriptional response to these two stressors in isolation: hypoxia altered 200 genes (FDR < 0.05, log_2_ | FC | > 0.25), enriched in HIF-1 and TGF-β pathways (Supplementary Fig. 7A and Supplementary Data 2), while TNF-α exposure (10 ng/mL, 24 h) affected 2,788 genes (FDR < 0.05, log_2_ | FC | > 0.25), many of which were linked to NF-κB and TNF-α signalling (Supplementary Fig. 8A and Supplementary Data 3). We observed that KLF6 overexpression synergised with hypoxia and TNF-α exposure. KLF6 overexpression under hypoxia altered expression of 1,576 genes (FDR < 0.05, log2|FC | > 0.25), impacting inflammation, autophagy, growth factor and oestrogen signalling (Supplementary Fig. 7B and Supplementary Data 4). With TNF-α, KLF6 influenced 9395 genes (FDR < 0.05, log_2_ | FC | > 0.25), which were enriched in FOXO, p53, PI3K-AKT, JAK-STAT, TNF-α and NF-κB signalling pathways (Supplementary Fig. 8B and Supplementary Data 5). In both treatment groups, KLF6-downregulated genes were enriched in cell cycle and DNA replication (Supplementary Fig. 7B and 8B), consistent with anti-proliferative effects of KLF6 overexpression seen in cultured HPAECs (Fig. 2). These findings highlight KLF6 as a key regulator of endothelial repair, angiogenesis, and of hypoxia- and TNF-α-regulated pathways central to PAH pathogenesis.

### Comparative analysis of transcriptomic changes induced by KLF2, KLF4, and KLF6 highlights unique effects of KLF6

KLF2, KLF4, and KLF6 are expressed in the pulmonary endothelium and share numerous structural and functional similarities, but the degree to which their roles are comparable remains unclear. To address this question, we overexpressed each KLF at comparable levels ( ~ 6-fold increase in protein expression in all KLFs, Supplementary Fig. 9A–D) in HPAECs. The extent of perturbation of the endothelial transcriptome by overexpression of either KLF2 or KLF4 was similar to that observed in KLF6-overexpressing cells (4,732 DEGs), with 4,256 and 4,622 DEGs for KLF2- and KLF4-overexpressing HPAECs, respectively (*p* value < 0.05, log_2_ | FC | > 0.25) (Supplementary Fig. 9E, F and Supplementary Data 6 and 7).

**Fig. 4:**
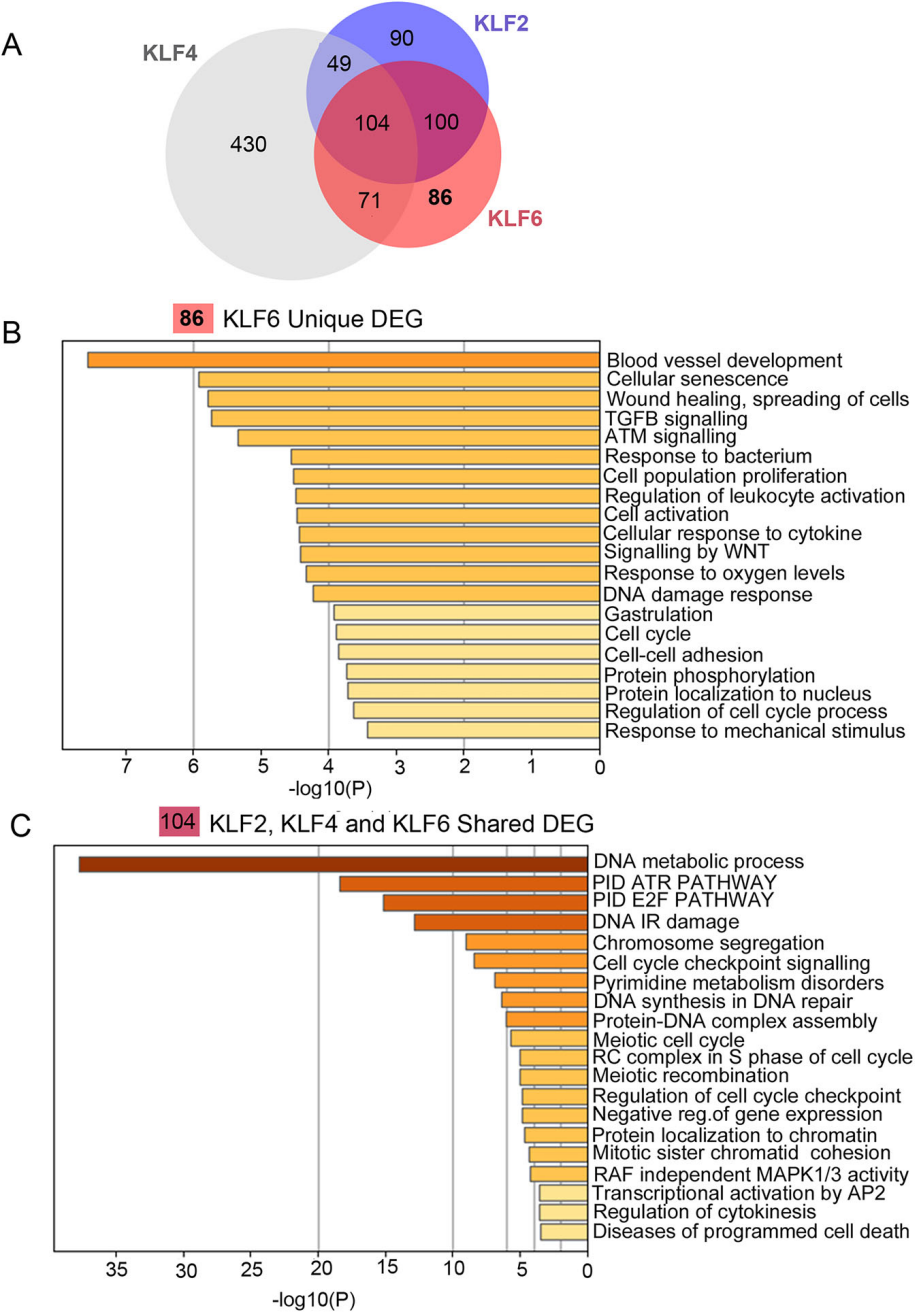
Metascape pathway and functional enrichment analysis of shared and unique KLF6-regulated DEGs. **A** Venn diagram shows the number of KLF2-, KLF4- and KLF6-regulated DEGs, with DEGs unique to KLF6 marked in red. **B** A corresponding bar graph shows Metascape pathway and process enrichment analysis of unique KLF6-regulated DEGs. **C** Metascape pathway and process enrichment analysis of shared DEGs. DEG selection criteria were FDR < 0.05, log2|FC | > 2 or <-2.

Focusing on the genes most impacted by the overexpression of either KLF factor (FDR < 0.05, log_2_ | FC | > 2), we detected 86 DEGs unique to KLF6, which were enriched in key functions promoting endothelial repair such as wound healing, vasculature development, TGF-β signalling and response to DNA damage (Fig. 4A, B). The 104 DEGs shared among KLF2, KLF4 and KLF6 (Fig. 4C) showed enrichment in processes controlling cell proliferation such as DNA metabolism, DNA replication and cell cycle regulation. Lists of unique and shared KLF2, KLF4 and KLF6-regulated DEGs are provided in Supplementary Tables 1 and 2.

Transcription factors often interact through feedback loops or hierarchies. The analysis of mutual regulatory interactions amongst the studied KLFs revealed that KLF6 increases expression of KLF2, but KLF2 inhibits expression of KLF6 (Supplemental Fig. 10A, C). This suggests that KLF2, which promotes endothelial quiescence and homoeostasis, may potentially restrict the actions of KLF6. No significant regulatory relationship was observed between KLF6 and KLF4 (*p* = 0.056, Supplementary Fig. 10B, D).

Collectively, these functional and transcriptomic analyses suggest that the net effect of KLF6 activation is twofold: minimizing injury and promoting endothelial regeneration.

### KLF6-regulated genes in PAH

**Fig. 5:**
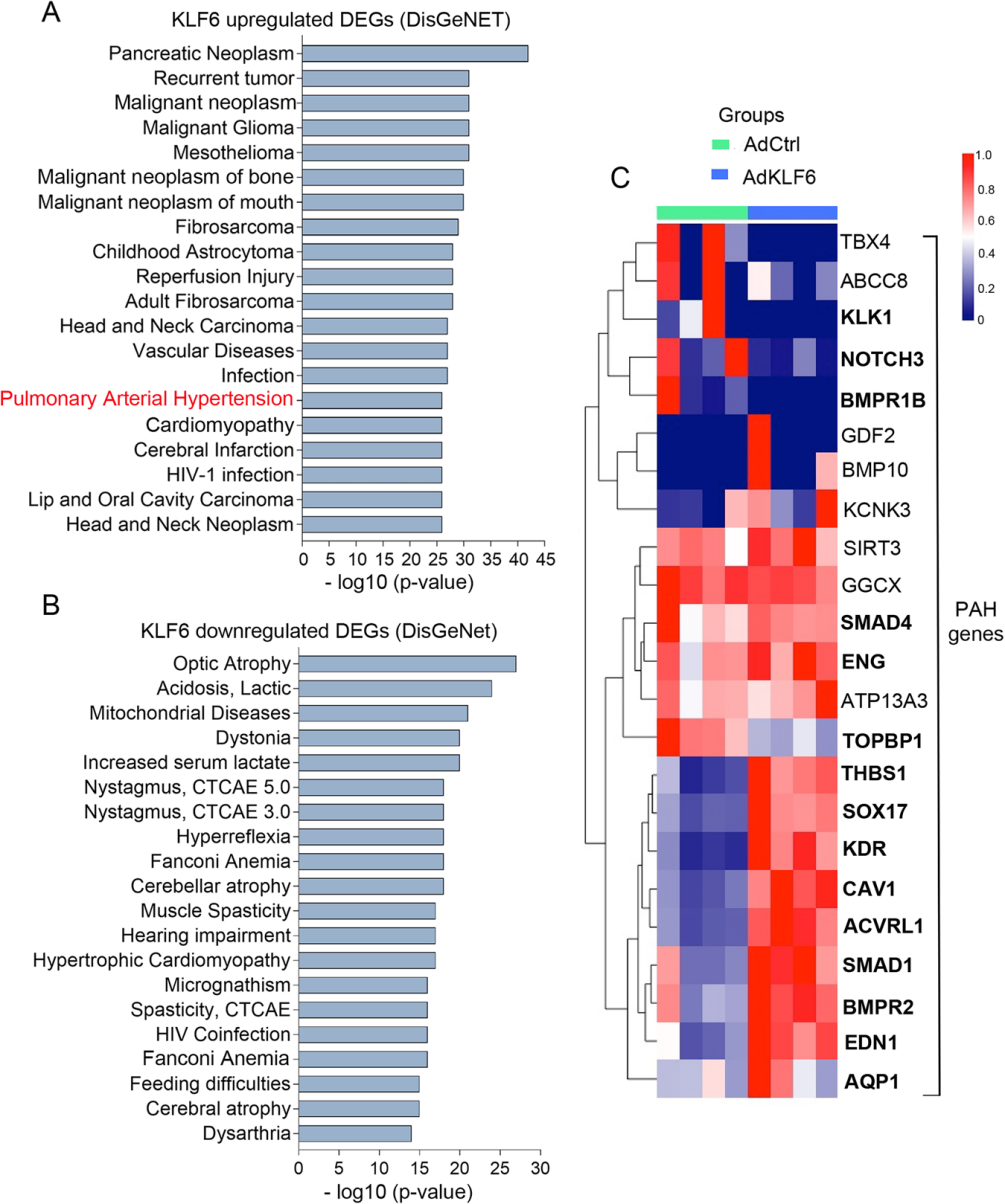
DisGeNET enrichment analysis of KLF6-regulated DEGs. Disease gene enrichment analysis of (**A**) upregulated and **B** downregulated KLF6 DEGs. Pulmonary hypertension terms are highlighted in purple.** C** Heatmap shows colour-coded changes in expression of PAH-associated genes, with hierarchical clustering in control and KLF6-overexpressing HPAECs. DEGs are marked in bold. Each column represents one experimental repeat; *n* = 4 independent samples/group.

Given the role of KLF6 in regulating endothelial responses to PAH stressors (Fig. 3), we examined its connection to PAH-related genes. DisGeNET disease enrichment analysis^32^ revealed significant associations of genes upregulated by KLF6 in HPAECs with vascular diseases, including PAH, whereas the down-regulated genes showed links with mitochondrial diseases and hypertrophic cardiomyopathy (Fig. 5A, B). Interestingly, KLF6 altered the expression of numerous genes with known H/IPAH-associated mutations, including *BMPR2, ENG, THBS1, SOX17, KDR, CAV1, ACVRL1, SMAD1, SMAD4, EDN1, ATP13A3, KLK1, NOTCH3, TOPBP1* and *BMPR1B* (Fig. 5C). Genes modulated by KLF6 in HPAECs also showed significant overlaps with publicly available sets of PAH and IPAH DEGs, including PAH HPAECs and ECFCs from microfluidic models of PAH^33^, IPAH PAECs^34^ and a previously curated list of PAH-associated genes (named herein as “known PAH genes”)^35^ (Supplemental Fig. 11 and Supplementary Data 8).

KLF6 also increased the expression of several genes implicated in the regulation of PASMC proliferation in PAH, including *EDN1*, *PDGFB* and TGF-β family genes^36^. To investigate this further, we co-cultured PASMCs with KLF6-overexpressing HPAECs. This led to significant stimulation of PASMC proliferation. This effect was prevented by a combined treatment with of ET-1 receptor antagonist, bosentan^37^ and PDGFR-β inhibitor, imatinib^38,39^ at clinically relevant doses (Supplementary Fig. 12).

**Fig. 6:**
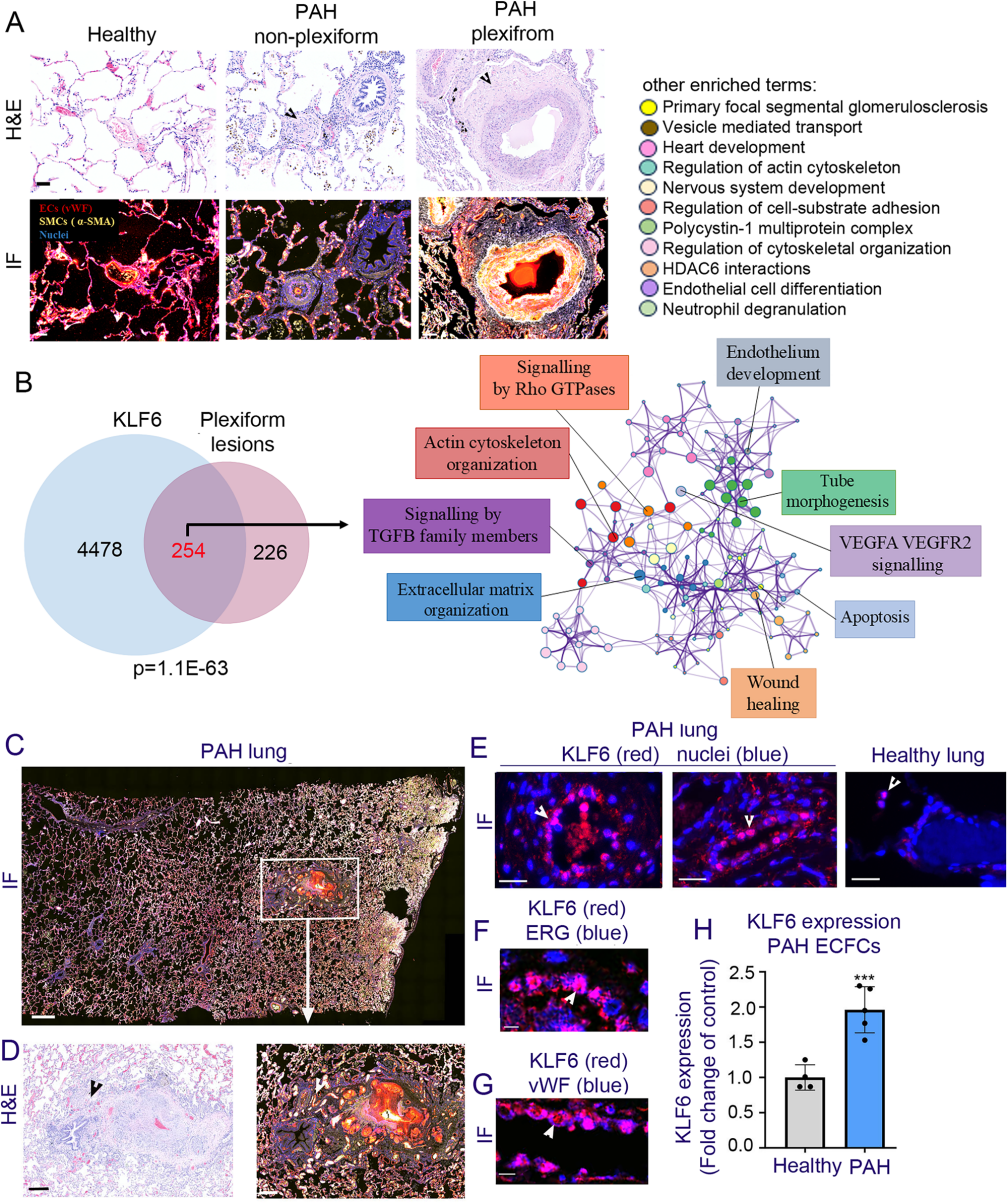
KLF6 in PAH endothelium. **A** Representative examples of healthy lung tissues, non-plexiform remodelled vessel and plexiform lesion in PAH lung used for spatial transcriptomics. H&E and IF staining was used to guide the selection of ROIs in GeoMx Digital Spatial Profiler. Von Willebrand factor (vWF; red), α-smooth muscle actin (α-SMA; yellow) and (SYTO 13, blue). 30–45 ROIs were selected in lung sections from healthy (*n* = 5) and PAH (*n* = 6) individuals, with separate annotation for non-plexiform plexiform lesions. **B** Venn diagram showing an overlap between KLF6-regulated DEGs in HPAECs and plexiform lesions DEGs identified by spatial transcriptomics; *P* = 31.1E–63, Fisher’s exact test in the GeneOverlap package in R. The associated Metascape network of enriched terms in the shared pool of DEGs coloured by cluster ID, is shown next to the Venn diagram. FDR < 0.05, log2|FC | < -0.25 or >0.25. **C** PAH lung section (Immunofluorescence) with a plexiform lesion within the boxed area. **D** Enlarged images of the plexiform lesion (H&E and IF staining), with arrowheads pointing to the area enlarged in (**E**). **E** Localization of KLF6 in PAH and healthy lung, as indicated. KLF6 is red and nuclei are blue; Arrowheads point to nuclear localization of KLF6 (pink). Panels** F**,** G** show KLF6, vWF and ERG staining, as indicated. Scale bars in (**A**) = 50 µm, in (**C**)  = 500 μm, in (**D**) = 200 µm, in (**E**) = 50 µm and in (**F**, **G**) = 10 µm. **H** KLF6 mRNA expression in healthy and PAH ECFCs. In graphs, bars are means ± SEM, ***P < 0.001, *n* = 4–5, Student *t* test. Source data for graph in (**H**) are provided in Supplementary data 12.

To examine potential contribution of KLF6 to vascular remodelling in PAH, beyond in vitro models, and to analyse site-specific changes in gene expression, spatial transcriptomic analysis of remodelled human PAH vasculature was carried out across two separate regions: (1) concentric intimal lesions with different degrees of medial and adventitial thickening and (2) vessels located within the complex plexiform lesions (Fig. 6A) [30 ROIs/lung in sections from patients with (*n* = 6)]. Equivalent size pulmonary arterioles from age- and sex-matched healthy were used as controls (*n* = 5). Plexiform lesions were identified based on established structural characteristics using α-SMA and CD31 immunostaining, along with spatial morphology consistent with published definitions^40,41^. Specifically, regions were selected where there was evidence of endothelial cell proliferation around remodelled vessels lacking a continuous media.

A total of 480 genes were detected as differentially expressed in plexiform lesions (comparison of vessels found within plexiform lesions versus controls; *p*-value < 0.05, log_2_ | FC | > 0.25) (Fig. 6B and Supplementary Data 9). Remarkably, more than half of plexiform lesion DEGs (254/480 genes) were identified as KLF6-regulated (*p* = 1.1E-63) (Fig. 6B and Supplementary Data 10). These KLF6-regulated plexiform lesion DEGs displayed significant enrichment in pathways and functions related to endothelial development, wound healing, tube morphogenesis, VEGF signalling, TGF-β signalling, extracellular matrix organization, Rho GTPase signalling and actin cytoskeleton organization (Fig. 6B). In non-plexiform, remodelled PAH vessels (displaying thickened intima and media) KLF6-regulated DEGs showed significant associations with growth factor signalling and extracellular matrix organization (Supplementary Fig. 13 and Supplementary Data 11).

Comparison of our data with the recently published spatial transcriptomic analysis of IPAH vasculature^42^ showed that KLF6-regulated genes constituted 53% of plexiform-specific DEGs, consistent with our findings (Supplementary Fig. 14 and Supplementary Data 10).

### KLF6 localises to lung endothelium in human and experimental PAH

Immunostaining of human PAH lung tissue revealed that, in the healthy lung, single KLF6⁺ cells were sparsely dispersed throughout the parenchyma and lacked a defined localization pattern (Supplementary Fig. 15A, C). In contrast, KLF6 expression in PAH lung tissues was markedly increased in the endothelial cells lining the vascular channels of plexiform lesions, showing an overall ~3.2-fold elevation compared with healthy lung tissues (Fig. 6C–G and Supplementary Fig. 15B, D, F and 16A–E). KLF6^+^ cells were also detected, albeit less frequently, in and around the remodelled vessels. Human placental tissue, known to express high levels of KLF6^43^, served as a positive staining control (Supplementary Fig. 15E). Lung tissue from a patient with tuberculosis, an infectious lung disease characterised by intense inflammation and lung-parenchymal damage^44^, taken as disease control, showed increased numbers of KLF6^+^ cells however, in contrast to PAH lung tissue, these cells did not show a collective pattern of localization (Supplementary Fig. 16F). KLF6^+^ cells, expressing the endothelial identity markers ERG and vWF (Supplementary Fig. 17A), often adopted a cuboidal shape and protruded into the lumen, suggestive of endothelial activation^45^.

A marked upregulation of KLF6 expression was also found in PAH blood-derived endothelial colony forming cells (ECFCs), compared with the corresponding healthy controls (Fig. 6H). These ECFCs display numerous characteristics of the diseased endothelium, including increased proliferative and angiogenic responses^46,47^ and are thought to play a contributory role in the formation of angioproliferative lesions in human PAH.

We also analysed archived lung tissues from pulmonary hypertensive MCT and Sugen/hypoxia rats, whose pulmonary hypertension phenotypes have been described previously^48^ and are summarized in Supplementary Fig. 18. We observed an accumulation of KLF6⁺ cells in these samples, with a relatively greater increase observed in the Sugen/hypoxia model (Supplementary Fig. 19A, B). However, the KLF6^+^ cells in these models exhibited a lower degree of structural organization compared with human PAH lung tissues.

To assess whether the molecular mechanisms we identified reflect broader aspects of pulmonary vascular pathology, we examined single-nucleus RNA-seq (snRNA-seq) data from samples of Alveolar Capillary Dysplasia with misalignment of the pulmonary veins (ACDMPV)^49^, a rare lung developmental disorder leading to persistent pulmonary arterial hypertension and fatal outcomes in newborns^50^. Recently, Guo et al. reported an integrated analysis of snRNA-seq of ACDMPV (*n* = 5) and control lungs (three 3-year-old and three preterm neonate lungs) and identified six endothelial cell (EC) subtypes, including capillary 1 (CAP1) cells, capillary 2 (CAP2) cells, systemic vascular ECs (SVECs; also known as bronchial vessels), venous ECs (VECs), arterial ECs (AECs), and lymphatic ECs (LECs)^49^. Given the pervasive role of KLF6 in regulating transcriptional programmes essential for endothelial homoeostasis, as we show in this study, and its involvement in PAH-associated vascular remodelling, we compared the expression of *KLF6* in ACDMPV vs. control lungs within each EC subtype using this integrated snRNA-seq dataset. The analysis revealed a disease-related increase in KLF6 expression in arterial ECs, and to a lesser extent in venous ECs, when compared to age-matched controls and only in arterial ECs when compared with preterm neonate controls (*n* = 3/group) (Supplementary Fig. 20 and Supplementary Table 3).

These results collectively demonstrate that the accumulation and structural reorganization of KLF6^+^ endothelial cells are characteristic of human PAH. The elevated expression of KLF6 in arterial endothelial cells in ACDMPV lungs implies a wider relevance of KLF6 upregulation in pulmonary vascular disease.

## Discussion

This study identifies KLF6 as a potential driver of the pulmonary endothelial transcriptional response to PAH-related stressors and as a significant feature of plexogenic arteriopathy.

KLF6 overexpression increased HPAEC tube formation, enhanced pulmonary arterial sprouting and significantly accelerated endothelial wound healing in vitro. These pro-angiogenic effects can be linked with KLF6-induced increase in the expression of blood vessel development genes^15,51^, such as SRY-box 17 (*SOX17*), ETS-related gene (*ERG*), VEGF receptors *FLT1* and *KDR*, angiopoietin receptor TIE2 (*TEK*), endoglin (*ENG*), activin receptor-like kinase 1 (ACVRL1), and bone morphogenetic protein receptor 2 (*BMPR2*) as well as increased expression of matrix metalloproteinases (*MMP2* and *MMP14*) and junctional proteins VE-cadherin (*CDH5*) and PECAM-1 (*PECAM1*). Interestingly, and perhaps counterintuitively, KLF6 also reduced the expression of genes involved in cell cycle progression and inhibited PAEC proliferation in vitro. Angiogenesis is a complex process involving a series of temporally and spatially controlled events involving dissolution and re-formation of cell-cell adhesions, cell migration and proliferation. Whilst the common perception is that “pro-angiogenic” factors should promote both proliferation and sprouting/migration of endothelial cells, recent studies revealed the existence of pro-angiogenic mechanisms that have an opposing effect on proliferative and migratory cellular responses^52^. For example, mitogen-induced cell cycle arrest is observed primarily in tip cells at the angiogenic front, while an increase in proliferating ECs can be observed in more mature and quiescent vascular regions^53^.

The observation of differential regulation of migration and proliferation in vascular tip cells is particularly intriguing, considering that the highest expression of KLF6 in the healthy human lung endothelium is observed in endothelial tip cells^53^ (Supplementary fig. S21). The effects of KLF6 activation or inhibition may also depend on the stage of the disease and the cellular context, similar to its target, BMPR2, which does not affect PAEC proliferation in vitro^54^, but can act as a pro-proliferative stimulus in the presence of BMP9^55^.

Spatial transcriptomic analysis of PAH lung vascular tissues uncovered a significant enrichment of KLF6-regulated genes in processes that drive vascular repair and angiogenesis. DisGeNET disease enrichment analysis^23^ confirmed a significant association between KLF6 and key PAH pathways such as FOXO, p53, TNF-α, NF-kB, PI3K-Akt, and Ras signalling^56–60^.

Our data, along with recently published spatial transcriptomic analysis of IPAH vasculature^42^ show that KLF6-regulated genes account for more than half of all DEGs found in plexiform lesions, and that the expression of genes mutated in hereditary PAH is significantly upregulated. Although this may seem paradoxical, both gain and loss of function in these genes, primarily from the TGF-β family, can be associated with vascular pathology in PAH, likely reflecting the complexity of signalling events in the context of additional environmental or genetic factors^61^.

Human PAH lung sections showed accumulation of KLF6^+^ vWF^+^ ERG^+^ cells within vascular channels in plexiform lesions and, to a lesser extent, in other parts of the remodelled lung. This selective overexpression of KLF6 in the PAH endothelium is corroborated by recent data showing that only 2 out of 8 endothelial cell clusters in PAH lung display a pro-angiogenic phenotype^62^. The MCT and Sugen/hypoxia rat lungs also showed increased numbers of KLF6^+^, however with less collective organization, compared with human PAH lung, which may reflect the limited ability of rodent models to fully replicate human plexiform-like arteriopathy^63^.

KLF6^+^ cells may potentially represent a subpopulation of endothelial progenitor cells, thought to accumulate in lung tissues in the end-stage PAH^46,64,65^. Consistent with this premise, we observed a significantly elevated expression of KLF6 in PAH blood-derived endothelial progenitor cells (also called the late outgrowth colony forming cells; ECFCs), often used as endothelial surrogates in PAH and other cardiovascular diseases^66^. One limitation of this approach is that PAH patients were generally older and receiving treatment at the time of blood collection. To account for possible effects of age and medication, future work should consider obtaining cells at diagnosis or performing longitudinal studies to assess whether PAH therapies exert lasting effects on cultured ECFCs. Although KLF6 activation has been observed primarily in PAH endothelial cells, we cannot rule out involvement of other cell types. For example, both human and murine macrophages express high levels of KLF6, where it plays a role in regulating immune responses^67^. Establishing the identity of KLF6^+^ cells through targeted functional genetics with single-cell multiomics will be vital to understand the role of KLF6 in the vascular remodelling in PAH.

Enhancement of angiogenesis was shown to have a positive impact, at least in animal models of PH^68,69^. For example, Elafin, a neutrophil elastase inhibitor, reverses severe PH in rats and improves angiogenesis in IPAH/HPAH PAECs by enhancing BMPR2 signalling in a caveolin-1- dependent manner^68^. Taking this into account, we could speculate that KLF6 activation, which significantly upregulates *BMPR2* and *CAV1* expression, may have a positive therapeutic impact. Consistent with the premise of beneficial pro-angiogenic and pro-survival signalling, the BMP4 signalling activator tacrolimus was shown to reverse severe PAH in rats and promote PAEC survival and angiogenesis in vitro – functions essential for preventing vessel loss and inducing vessel regeneration in PAH^69^. Given that angiogenesis can also contribute to the formation of vascular occlusions in PAH^70^, chronic KLF6 activation could also have a detrimental effect. The mediators of PASMC proliferative response need to be identified, but the inhibitory effects of drugs used in PAH therapy, bosentan^37^ and imatinib^38^, suggest that ET-1 and PDGF-β, both downstream targets of KLF6, may play a contributory role. It is conceivable that KLF6, much like its target gene VEGF^71^, can play a dual role, offering early protective effects but contributing to pathogenic processes at later stages of the disease.

A key question that remains is how KLF6 signalling can be targeted. Whilst specific pharmacological inhibitors or activators of KLF6 have not been identified, in cancer cells KLF6 expression can regulated by a number of factors, including HIF2α-activated and H3 acetylation-dependent super-enhancer^72^, miR-191-5p, miR-200c-3p, miRNA-543^73–75^, long non-coding RNA CR749391^76^, transcription factor SP2 and HIF-1^77–79^.

Our results suggest a regulatory interplay among KLF2, KLF4, and KLF6. Under PAH-related stress conditions, KLF6 expression increased as KLF2 and KLF4 were suppressed, possibly in a compensatory response^12,13^, and KLF6 and KLF2 showed evidence of reciprocal regulation. Though we did not see a direct relationship between KLF4 and KLF6 expression, these two transcription factors are likely to interact via their transcriptional targets. The N-terminal domain of KLF6 is responsible for recruiting and interacting with different transcription factors and cofactors, including Sp1, KLF4, p53, hypoxia-inducible factor 1 alpha (HIF1α), runt related transcription factor 1 (RUNX1), E2F1, GTF3C1 and histone deacetylase 3 (HDAC3), to regulate the transcription process in a context-dependent manner^67^. Characterizing the complex crosstalk between KLF2, KLF4, and KLF6, will require further investigation.

To determine whether the molecular mechanisms identified may represent more general features of pulmonary vascular disease, we analysed a alveolar capillary dysplasia (ACD) snRNA-seq dataset^80,81^. Although ACD is distinct from PAH, the two conditions are characterised by hypoxaemia and pulmonary hypertension and share several key pathological features, including the loss of capillaries, occlusive remodelling of the pulmonary arterial circulation and dysregulated angiogenesis^50^. Through this analysis, we identified an elevation in KLF6 expression within pulmonary arterial endothelial cells, suggesting that KLF6 may potentially act as an activator of shared molecular pathways prominent in PAH and ACD^50^, including vascular endothelial growth factor (VEGF)/VEGFR signalling, angiopoietin, hypoxia-inducible factor 1α, transforming growth factor-β, bone morphogenetic protein and NF-κB signalling.

In summary, we show that KLF6 activation is a hallmark of angio-proliferative pulmonary vascular endothelial phenotype in human PAH. Dysregulation of KLF6 signalling may have broader implications for pulmonary vascular disease.

## Methods

Detailed description of experimental protocols been provided in Supplemental Methods.

### Cell culture

Human Pulmonary Artery Endothelial Cells (HPAECs) were obtained from PromoCell (Germany, Cat. No. C-12241) and Human Pulmonary Artery Smooth Muscle Cells (HPASMCs) were obtained from Lonza (Walkersville, USA, Cat. No. CC-2581). The cells were used between passages 5-10. Donor information is provided in Tables M1 and M2 in Supplemental Methods. Cells were cultured, as described in ref. ^12^.

### Peripheral blood mononuclear cells (PBMCs) and endothelial colony forming cells (ECFCs)

Venous blood samples were obtained from healthy volunteers (*n* = 5) and from heritable PAH (HPAH) patients with rare pathogenic BMPR2 variants (*n* = 5)^33^ with the informed written consent and approval of the local ethics committee (REC Ref. 17/LO/0563) at the Pulmonary Hypertension Clinic, Hammersmith Hospital, Imperial College London. Demographic and clinical characteristics of HPAH patients and healthy volunteers are shown in Table M3 in Supplemental Methods.

### Adenoviral overexpression KLF2, KLF4 and KLF6 in HPAECs

The overexpression of most common (long) isoforms of KLF2, KLF4 and KLF6 was achieved by adenoviral gene transfer AdKLF2-GFP (Cat. No. ADV-213187), AdKLF4-FLAG (Cat. No. ADV- 213191), AdKLF6-HA (Cat. No. ADV- 213194); Vector Biolabs (Pennsylvania, USA). Adenoviral control (AdCTRL-GFP) for AdKLF2-GFP was AdGFP (Vector Biolabs, Cat. No. 1060), while AdTet-off^82^ was kind gift from Professor Stuart Yuspa (National Cancer Institute, NIH, Bethesda, USA) and was used as adenoviral control (AdCTRL) for AdKLF4 and AdKLF6.

### KLF6 silencing

The knockdown of KLF6 expression was performed using silencer select siRNA targeting KLF6 (siRNA-KLF6, Thermo Fisher Scientific, UK, Cat. No. s3376). Control siRNAs included a scrambled siRNA control (siRNA-CTRL Cat. No. 4390846) and a positive siRNA control targeting GAPDH (siRNA-GAPDH, Cat. No. 4390849).

### HPAEC culture under flow

HPAECs were cultured in Nunc Slide Flaskettes (Thermo Fisher Scientific, UK, Cat. No. 170920) until 95% confluent. The bottom slides with the cells were then detached from the flask and placed inside the flow chamber in a parallel flow apparatus^83^. The cells were then subjected to a laminar flow at 4 dynes/cm^2^, physiological for lung arteries^84^, for different periods (30 mins,1 h, 4 h, 6 h and 24 h). After the flow exposure, the cells were used for RNA extraction and RT-qPCR to measure mRNA expression levels of KLF2, KLF4 and KLF6.

### Real-time quantitative PCR (qPCR)

Protocols for RNA extraction and RTq PCR are provided in Supplemental Methods.

### RNA sequencing

RNA sequencing (RNAseq) (75 bp paired-end reads) was performed in an Illumina NextSeq 2000 sequencer (Illumina, USA) at the Imperial BRC Genomics Facility (Imperial College London) to assess the whole transcriptomic effects of KLF2, KLF4, and KLF6 overexpression by adenoviral gene transfer (AdKLF2, AdKLF4 and AdKLF6 conditions, respectively).

The quality of the paired end reads within the fastq files were assessed using FastQC (https://www.bioinformatics.babraham.ac.uk/projects/fastqc/) and MultiQC. To determine transcript abundance values, Salmon (v1.5.2) was used to map sequencing reads in the fastq files to the human genome (GENCODE v44) (https://www.gencodegenes.org/human/) and to obtain gene counts. To identify differentially expressed genes (DEGs), the gene expressions of the AdKLF2, AdKLF4 and AdKLF6 conditions (n = 4 biological replicates/condition) were compared with the corresponding negative control conditions using the R package DESeq2 (v1.38.3)^85^, with default settings. Enhanced volcano (v1.16.0) (https://github.com/kevinblighe/EnhancedVolcano 2020) and heatmaps were generated using the R package pheatmap (v1.0.12). Gene annotation was performed using biomaRt (2.46.3). The list of PAH genes was derived from^86^.

### Gene ontology (GO) and kyoto encyclopaedia of genes and genomes (KEGG) enrichment analysis

GO and KEGG pathway enrichment analyses were carried out separately on the sets of up- and down-regulated genes from each comparison using the Metascape (https://metascape.org) online tool^23^, the WEB-based GEne SeT AnaLysis Toolkit (WebGestalt) (https://www.webgestalt.org)^87^ and the clusterProfiler package (v3.14.3) in R. The DisGeNET enrichment analysis and network of enriched terms of up- and down-regulated genes was performed using Metascape. Genes that passed the thresholds of FDR < 0.01, absolute value of log_2_ (fold change)>2 were used for GO and KEGG pathway enrichment analyses, unless indicated otherwise.

### Comparative analysis with published RNA-seq datasets and disease-gene databases

Comparative analysis was carried out using RNA-seq datasets from HPAECs and ECFCs from the “double hit” microfluidic model of PAH^33^ and from PAECs that had been isolated from IPAH patients^34^ or with known PAH and IPAH-related genes from DisGeNET databases (https://www.semanticscholar.org/paper/GeneOverlap%3A-An-R-package-to-test-and-visualize-Shen/117e12840af966176bbc348db6edf034b0ea479c). Overlapping genes were visualized as UpSet plots using the ComplexUpset package (v1.3.3) in R. The significance of the overlaps between gene sets in comparison to the genomic background was calculated by one-tailed Fisher’s exact test using the GeneOverlap package (v.1.22.0) in R

### Spatial transcriptomics

To assess the transcriptomic changes in PAH lungs, spatial transcriptomics of lung tissues from healthy controls and PAH patients were carried out using NanoString GeoMx® to enable high-plex spatial profiling of RNA or protein within specific areas of interest in the tissue^88^

Formalin fixed and paraffin embedded (FFPE) lung tissue sections (3–4 μm) from PAH patients (*n*  =  6) and age- and sex-matched healthy volunteers (*n*  =  5) were obtained from the Royal Papworth Hospital NHS Foundation Trust Tissue Bank (Cambridge, UK) with informed written consent and ethical approval by (ICHTB HTA licence: 12275; REC Wales approval: 22/WA/0214). Donor information for FFPE lung tissue samples is shown in Table M8 in Supplemental Methods.

### Spatial transcriptomics data analysis and visualization

The raw counts were processed using NanoString’s GeoMx NGS pipeline software V2.2 (Nanostring Technologies, USA), where they were converted into digital count conversion (DCC) files. The GeomxTools package (V3.5.0) was used for quality control (QC), and the downstream analysis of the DCC Data s was performed using R. Differential gene expression analysis was performed using a linear mixed model (LMM) as recommended in the GeomxTools manual. The adjusted *p* values were calculated using the Benjamini-Hochberg multiple test correction, and differentially expressed genes in all analyses were defined as FDR < 0.05 and absolute value of log_2_ (fold change) > 0.25. For spatial deconvolution, the SpatialDecon R package (https://www.nature.com/articles/s41467-022-28020-5) was used to identify the cell type composition of each ROI using the IPF Lung Cell Atlas (https://www.science.org/doi/10.1126/sciadv.aba1983) as the cell profile matrix, which is composed of 36 cell types.

Gene ontology and KEGG pathway analyses were performed using the WEB-based Gene SeT AnaLysis Toolkit (WebGestalt) (https://www.webgestalt.org).

^87^ The ComplexUpset R package (v1.3.3) was used to visualize the number of overlapping genes between different DE gene datasets (https://zenodo.org/records/7314197).

The GeneOverlap R package (v.1.22.0) was used to test the degree and significance of overlap between two gene lists in comparison with a genomic background using Fisher’s exact test (https://www.semanticscholar.org/paper/GeneOverlap%3A-An-R-package-to-test-and-visualize-Shen/117e12840af966176bbc348db6edf034b0ea479c).

### Analysis of endothelial *KLF6* expression in single nucleus RNA sequencing of alveolar capillary dysplasia with misalignment of pulmonary veins (ACDMPV) and control lungs

Integrated single nucleus RNA-seq (snRNA-seq) of ACDMPV and control lungs were obtained from Guo et al^49^., which contains snRNA-seq of five ACDMPV lungs (2 weeks to 3.5 years), three 3-year-old control lungs, and three preterm neonate lungs (1–4 days old, 29-31 weeks of gestational age). Detail donor information can be found in Guo et al^49^. We subset the integrated data to the identified endothelial cells (ECs). Gene expression was SoupX-corrected and normalized by Seurat NormalizeData function. Differential expression test was performed in each EC cell type for *KLF6* between ACDMPV vs. 3-yr control lung cells and between ACDMPV vs. preterm neonate lung cells.

Tests were performed using the Seurat 4 FindMarkers function using Wilcoxon Rank Sum test. Genes with the following criteria were considered up in an EC subtype in ACDMPV: p_val<0.05, log2FC > =log2(1.5), pct.1 > = 0.2, “ident1.pct > =20.nSample” >=2 (i.e., pct > =20% in at least 2 ACDMPV samples) in the cell type. Genes with the following criteria were considered down in an EC subtype in ACDMPV: p val<0.05, log2FC <= -log2(1.5), pct.2 > = 0.2, “ident2.pct > =20.nSample” >=2 (i.e., pct > =20% in at least 2 Control or 2 Preterm samples) in the cell type.

### Immunocytochemistry (ICC) and immunohistochemistry (IHC)

Protocols and reagent lists are provided in Supplemental Methods.

### NF-κB luciferase reporter assay

HPAECs were left untreated or were infected with adenoviral NF-κB luciferase reporter^89^ (AdNFkB-luc, Vector Biolabs, Cat. No.1740), AdCTRL and AdKLF6 or were transfected with siRNA-KLF6 and scrambled-siRNA control, as required. Since the basal KLF6 expression in unstimulated, quiescent HPAECs was insufficient to produce a measurable effect, the effects of KLF6 silencing were studied in cells cultured under flow. 3 hours after adenoviral infection or 48 h after siRNA exposure, the culture media were replaced with a fresh EGM2 medium with or without 10 ng/mL of TNF-α (R&D Systems, USA, Cat. No. 210-TA-020). The cells were then incubated under either normoxic or hypoxic conditions for 24 h. A luciferase reporter assay (Promega, USA, Cat. No. E1500) was then performed according to the manufacturer’s instructions. The intensity of luminescence, proportional to the level of NF-κB–driven luciferase activity, was measured in GloMax® luminometer (Promega, UK).

### Transwell permeability assay

Endothelial permeability assay was measured in cells grown in Transwell plates (Corning, USA, Cat. No. 3413). Passage of 40 kDa FITC-Dextran (Sigma-Aldrich, Dorset, UK, Cat. No. FD40S) following cell treatments was measured at excitation/emission 490/525 nm using a GloMax® luminometer (Promega, UK)^12^.

### Angiogenesis assays

Angiogenesis was assessed in a tube formation assay in vitro and in an ex vivo pulmonary arterial explants sprouting assay. For the tube formation assay, HPAECs infected with either AdCTRL or AdKLF6 were seeded into a 96-well plate pre-coated with growth factor-reduced Matrigel (Scientific Laboratory Supplies, UK, Cat. No. 354230). After 20 hours of incubation, endothelial tube formation was imaged using a phase*-*contrast microscope. Images were then analysed using ImageJ software (Fiji, version 2.14.0) with an Angiogenesis Analyzer plugin to quantify the number of nodes and meshes as well as the total tube length.

For the arterial explants sprouting assay, surgical specimens of human pulmonary arteries were cleaned of blood and attached connective tissues in PBS. Donor characteristics—including age, gender, height, weight and diagnosis—are summarized in Table M11 in Supplemental Methods. AdCTRL or AdKLF6 was added on top of the endothelial layer, and the explants were then incubated for 3 h in a humidified incubator. Afterwards, the arterial tissue was washed, cut into roughly 1 mm^2^ fragments which were then embedded in Matrigel and incubated for 3 weeks in a humidified incubator under normoxic conditions. Endothelial sprouts were fluorescently labelled with Vybrant™ CFDA SE Cell Tracer (Invitrogen, USA, Cat. No. V12883) before being imaged under a fluorescent microscope. The total area of sprouting was measured using ImageJ software (Fiji, version 2.14.0).

### EdU cell proliferation assay

Cell proliferation assay was performed using an EdU Cell Proliferation Assay Kit (EdU-594, EMD Millipore Corp, USA, Cat. No. 17-10527), according to the manufacturer’s instructions.

### Proliferation of PASMCs in co-culture with HPAECs

Control (AdCTRL) and AdKLF6-overexpressing HPAECs were grown to confluence in 6.5 mm Transwell inserts with 1.0 µm pore size transparent PET membrane (Corning, Cat. No 353104) in EGM2 culture media containing serum and growth factors. HPASMCs were seeded at the bottom of 24-well plates (50 × 10^3^ cells/well) or on the other side of empty Transwell inserts or inserts containing endothelial cells (20 × 10^3^ cells/insert) in EGM2 medium containing 5% FBS and no growth factors. Following 2-h incubation in a humidified culture incubator, unattached PASMCs were washed away with PBS and Transwell inserts containing HPAECs or co-cultures of HPAECs and HPASMCs were inserted into the wells of 24 well plate. Culture media were replaced with EGM2 medium containing 0.2% FBS, antibiotics and EdU (for further detail please see EdU Cell Proliferation Assay), with no growth factors. In some wells, ET-1 receptor antagonist bosentan^37^ and tyrosine kinase inhibitor, imatinib^38^ were added at clinically relevant doses, previously shown to inhibit ET-1-induced PASMC proliferation (bosentan, Stratech S3051; 20 µmol/L)^37^ and PDGF-β-induced PASMC proliferation (imatinib, Enzo ALX-270-492; 10 µmol/L)^33,38,39^. PASMCs cultured in full PASMC medium served as a positive control.

### Wound healing assay

Endothelial cell migration was assessed in a wound healing assay using ibidi Culture-Insert 2 Well in µ-Dish 35 mm (ibidi, Germany, Cat. No. 81176) or in a scratch assay under flow.

The wound area was measured using a custom-made Wound Healing Tool ImageJ macro (written by Stephen Rothery, Facility for Imaging by Light Microscopy (FILM), Imperial College London).

### Apoptosis caspase-Glo 3/7 assay

Apoptosis was assessed by measuring caspase 3/7 activity using a Caspase-Glo 3/7 Assay Kit (Promega, Southampton, UK, Cat. No. G8091), according to the manufacturer’s instructions.

### TUNEL apoptosis assay

TUNEL Assay was performed using a Click-iT Plus for In Situ Apoptosis Detection Kit with Alexa Fluor 647 dye (Invitrogen, Cat. No. C10619), according to the manufacturer’s instructions.

### Animal experimentation

All animal studies were approved by the University of Sheffield Animal Welfare and Ethical Review Body and conducted under the authority of a UK Home Office project licence, in accordance with the UK Animals (Scientific Procedures) Act 1986 and Directive 2010/63/EU of the European Parliament on the protection of animals used for scientific purposes.

In this study, we used archived samples from healthy and pulmonary hypertensive MCT and Sugen/hypoxia rats used in our previously published work^48^. Briefly, the Sugen-hypoxia model, PAH was induced in male Wistar (Charles River, UK) rats of 200–220 g by a single subcutaneous injection of Sugen5416 (Tocris, Bristol, UK) at 20 mg kg−1 followed by housing in hypobaric chambers at an equivalent of 18,000 ft for 3 weeks, followed by normobaric pressures for remaining 6 weeks. In the MCT model, PAH was induced in male Sprague Dawley rats (Charles River UK) by a single subcutaneous injection of monocrotaline (MCT, Sigma Aldrich, St. Louis, MO, USA) at 60 mg kg^−1^ in rats 200–210 g, alongside saline injected control animals. Animals were sacrificed at day 3, 7 and 14 post-MCT injection.

Left and right ventricular catheterisation was performed under isoflurane-induced anaesthesia (maintenance at 1.5% after induction with 3% isoflurane) by using a closed chest method. Following haemodynamic measurements, animals were maintained under deep anaesthesia with isoflurane inhalation (4%) and euthanasia was performed by exsanguination with cardiac puncture followed by cervical dislocation in accordance with institutional and national ethical guidelines for animal care and use. Tissues were then harvested for the analyses.

### Study approval

All ethical regulations relevant to human research participants were followed. Venous blood samples were obtained with the informed written consent and approval of the local ethics committee (REC Ref. 17/LO/0563) from healthy volunteers and from HPAH patients with a rare pathogenic variant of *BMPR2* gene at the Pulmonary Hypertension Clinic, Hammersmith Hospital, Imperial College London.

Human artery tissue samples were obtained following ethical approval from the Imperial College Healthcare Tissue Bank (ICHTB HTA licence: 12275; REC approval: 17/WA/0161).

Formalin fixed and paraffin embedded lung tissue sections from healthy donors and PAH patients were obtained from the Royal Papworth Hospital NHS Foundation Trust Tissue Bank (Cambridge, UK) with informed written consent and ethical approval by (ICHTB HTA licence: 12275; REC Wales approval: 22/WA/0214).

### Statistics and reproducibility

All graphs and statistical analyses were performed using either GraphPad Prism software 9 (GraphPad Software, USA) or Rstudio (RStudio Inc, version 1.2.5042).

All experiments were performed in at least four biological replicates, with 3 technical replicates performed per experiment, unless stated otherwise. All data were tested for normal distribution using the Shapiro-Wilk test. An unpaired student’s t test was used to analyse the normally distributed data from two sample groups, while a one- or two-way ANOVA test was used to analyse three or more sample groups as appropriate.

Statistical significance was accepted when *p-*values were less than 0.05. All error bars are representative of mean ( ± SEM).

## Supplementary information

Transparent Peer Review file

Supplementary Information

Description of Additional Supplementary Files

Supplementary Data 1

Supplementary Data 2

Supplementary Data 3

Supplementary Data 4

Supplementary Data 5

Supplementary Data 6

Supplementary Data 8

Supplementary Data 7

Supplementary Data 9

Supplementary Data 10

Supplementary Data 11

Supplementary Data 12

## Data Availability

Transcriptomic data are available from the European Genome Phenome Archive (EGA) under accession numbers: EGAS50000001853 (Bulk RNAseq of KLF2, KLF4 and KLF6 overexpressing HPAECs) and EGAS500000018534 (Spatial Transcriptomics of Pulmonary Vascular Remodelling in IPAH). Source data for all graphs are provided in Supplementary Data 12. All other data are available from the corresponding author on reasonable request.
