# Supplementary material for: KLF6 activation marks an angiogenic and apoptosis resistant endothelial phenotype in pulmonary arterial hypertension
Source: Commun Biol. 2026 Jun 15;9:846. doi: 10.1038/s42003-026-10493-5 (PMC13282386; doi:10.1038/s42003-026-10493-5)
Supplement: Supplementary file 2 — Supplementary Information [file 42003_2026_10493_MOESM2_ESM.pdf]

Alhharbi et al.

Supplemental Figures - p. 2-22

Supplemental Tables - p. 23-26

Supplemetal Methods - p. 27-49

Supplemental References - p. 48-49

SUPPLEMENTAL FIGURES

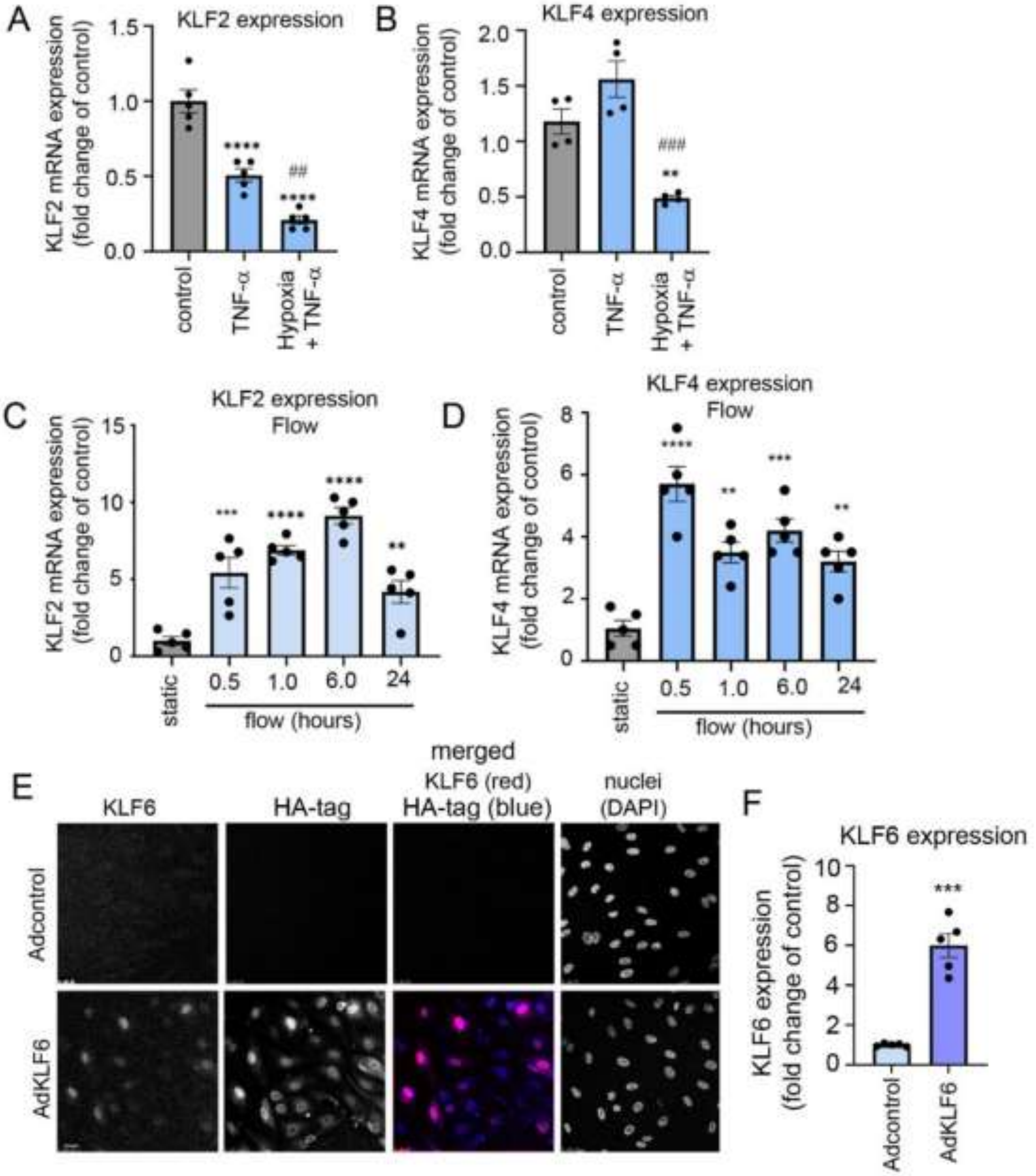

Supplementary Figure 1. KLF2, KLF4 and KLF6 overexpression in HPAECs under different experimental conditions. ***(A)*** *KLF2 and* ***(B)*** *KLF4 mRNA expression in HPAECs exposed to TNF-α (10 ng/ml, 24h) under normoxic or hypoxic (2% O_2, 24h) conditions, as indicated.* ***(C)*** *and* ***(D)*** *show KLF2 and KLF4 expression in HPAECs exposed to flow (4 dynes/cm2 0-24h), respectively.* ***(E)*** *Representative confocal microscopy images showing nuclear localization of recombinant HA-tagged KLF6 (AdKLF6) in HPAECs 24 hours post-infection. Scale bar =10μm; n=3.* ***(F)*** *KLF6 protein and* ***(G)*** *KLF6 mRNA levels in HPAECs infected with AdCTRL or AdKLF6. **P<0.01, ***P<0.001, ****P<0.0001, comparison with relevant study controls. ##P<0.05 and ###P<0.001, comparisons between TNFα and TNFα+hypoxia, as indicated. In (A-D) one-way ANOVA with Tukey post-test, n=5; In* ***(F, G)*** *unpaired t-test; n=3 in (F) and n=5 in (G). Bars are means ±SEM.*

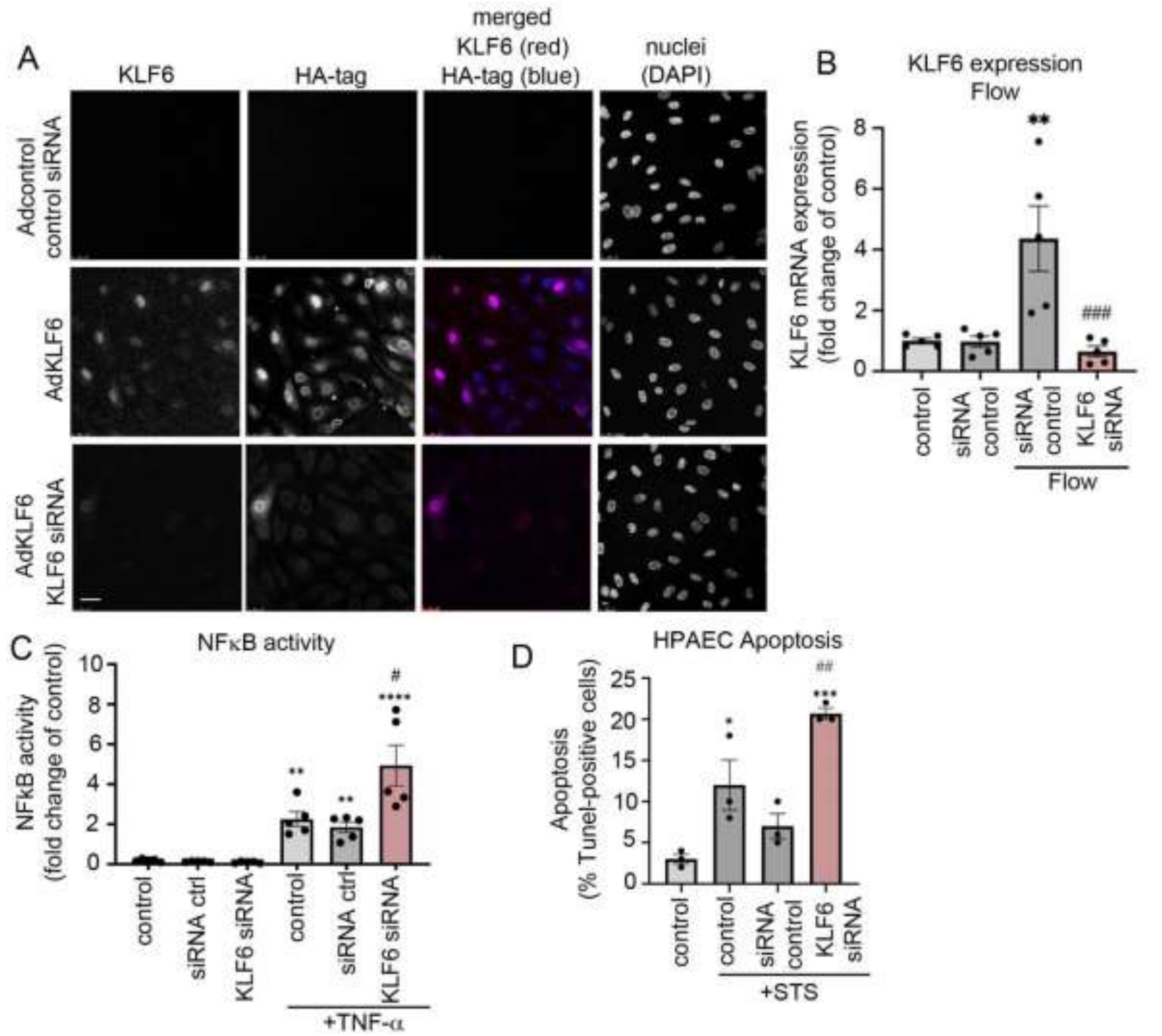

Supplementary Figure 2. Effects of KLF6 silencing in HPAECs. (A) *Representative confocal microscopy images showing localization of endogenous and recombinant HA-tagged KLF6 (AdKLF6) in HPAECs transfected with control, non-targeting siRNA or KLF6 siRNA, as indicated. HPAECs were transfected with siRNA 6 hours post-adenoviral infection and incubated for further 48 hours before immunostaining. Scale bar=10µm.* ***(B)*** *KLF6 mRNA levels in HPAECs transfected with control siRNA or KLF6 siRNA and exposed to flow (4 dynes/cm^2; 24h).* ***(C)*** *NFκB activity (luciferase reporter assay) in HPAECs transfected with control or KLF6 siRNA and treated with TNF-α (10 ng/ml, 24h) under flow (4 dynes/cm^2, 24h);* ***(D)*** *Apoptosis (Tunnel assay) in HPAECs transfected with control or KLF6 siRNA and treated with staurosporine (STS, 0.1µM, 24h) under flow. *P<0.05, **P<0.01, ***P<0.001, ****P<0.0001, comparisons with controls; $^{\#}$P<0.05, $^{\#\#}$P<0.01, $^{\#\#\#}$P<0.001, comparison with relevant treatment controls; one-way ANOVA with Tukey post-test. Bars are means ±SEM. In* ***(B, C)*** *n=5, in* ***(D)*** *n=4.*

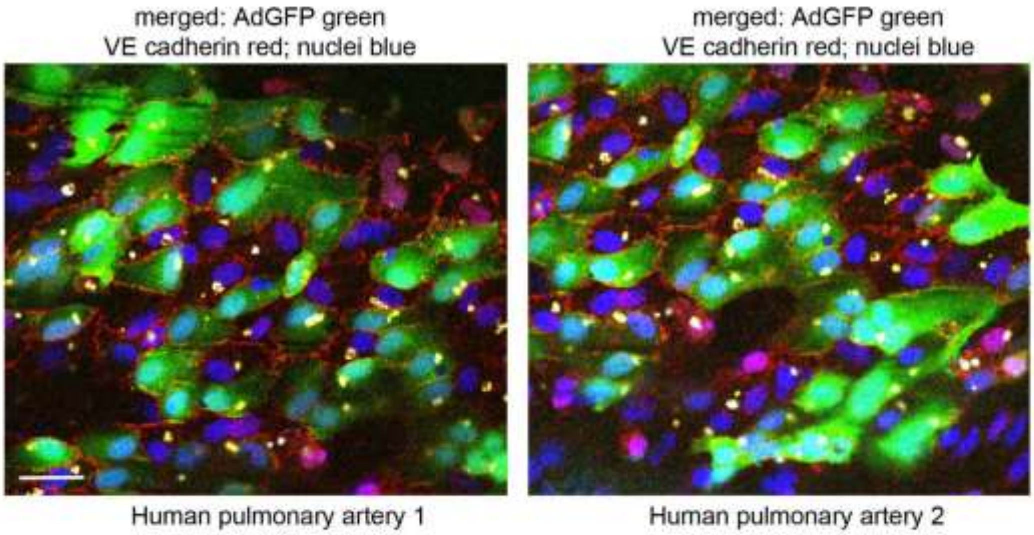

Supplementary Figure 3. Adenoviral transduction efficiency in the endothelium of human pulmonary artery explants*. Surgically removed pulmonary arteries were opened up and the endothelial side was incubated with AdGFP for 24 hours. In confocal images of the pulmonary arterial endothelium shown above, the cells overexpressing AdGFP are green, VE-cadherin is red and nuclei are blue, as indicated. Bar=20 µm.*

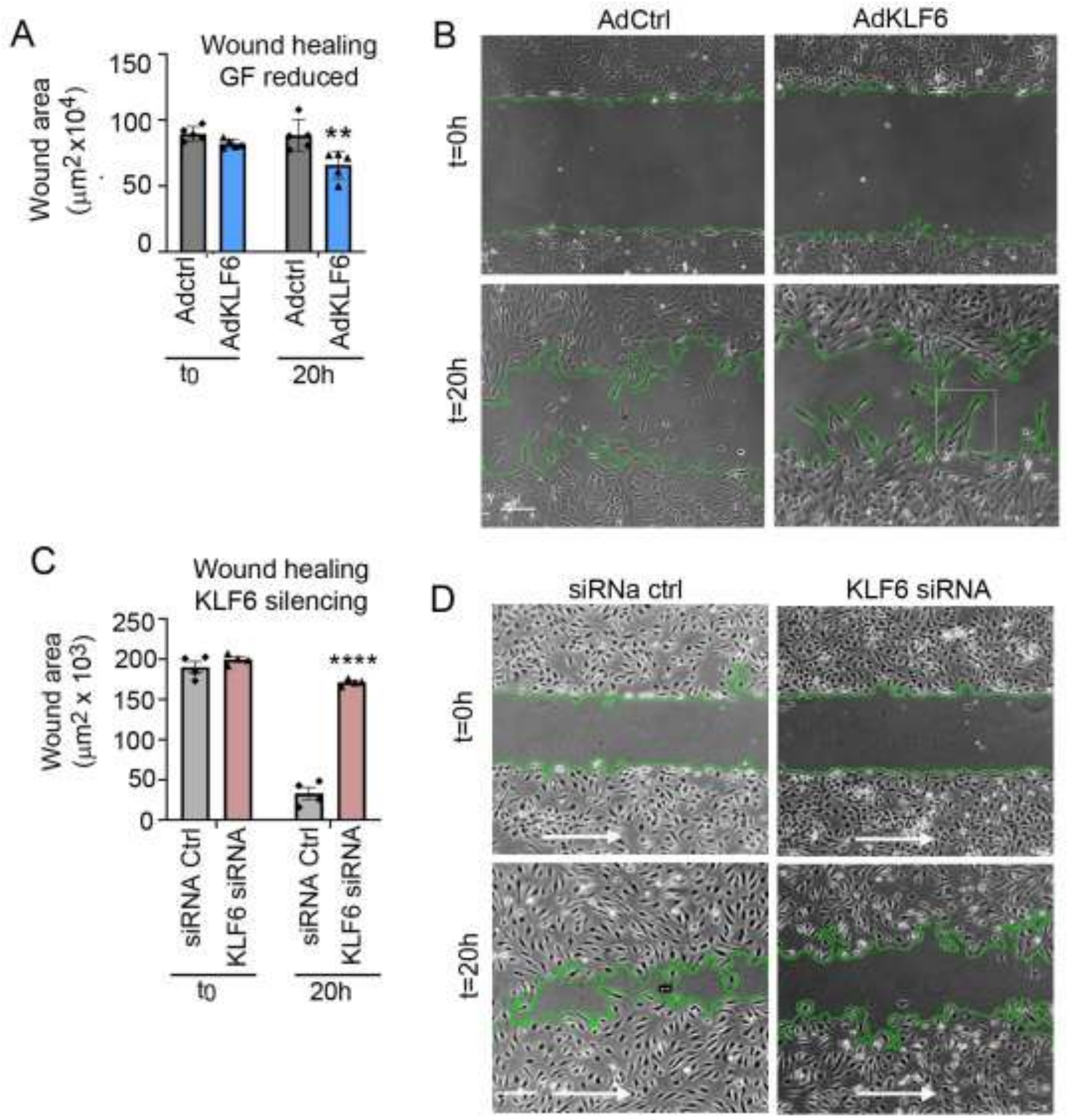

Supplementary Figure 4. Effects of KLF6 overexpression and silencing on endothelial wound healing in vitro. *Graph in **(A)** and corresponding light microscopy images in **(B)** show endothelial wound healing (measured as reduction in the wound area), in serum-and growth factor-starved control (AdCTRL) and KLF6-overexpressing (AdKLF6) HPAECs at t=0 and t=20h post-wounding. In **(B)** Boxed area shows well polarised AdKLF6-overexpressing cells migrating into the wound area. Graph in **(C)** and corresponding representative images in **(D)** show wound healing in flow-exposed (4 dynes/cm^2) HPAECs 48h post-transfection with control siRNA or KLF6 siRNA at t=0 or t=20h post-wounding. Green line demarcates wound edges and the arrow indicates direction of flow. **P <0.01, ****P<0.0001, comparison with relevant treatment controls, one-way ANOVA with Tukey post-test; n=4-5. Bars show mean ±SEM.*

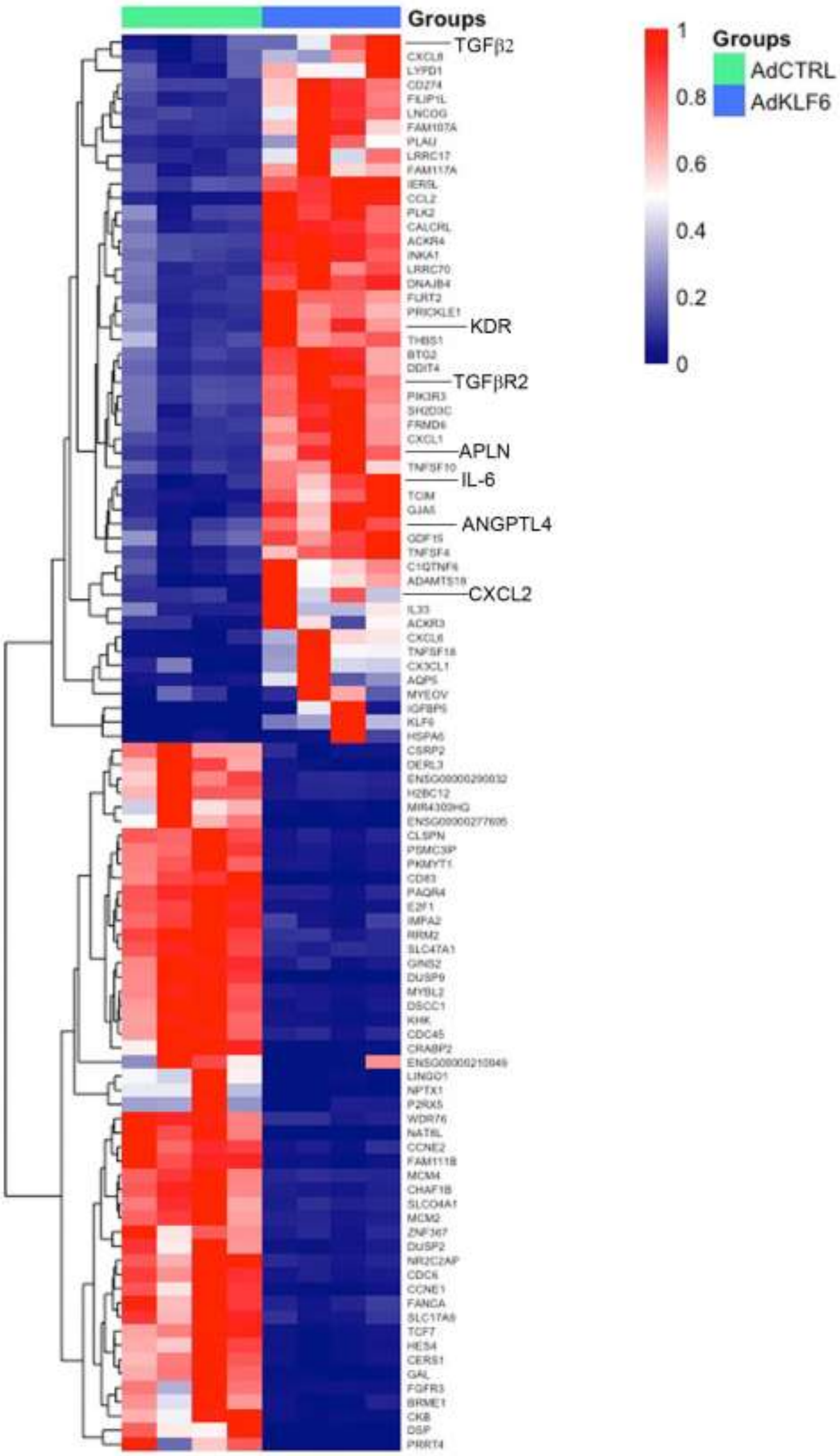

Supplementary Figure 5. Heatmap of top 50 upregulated and 50 downregulated KLF6-regulated genes. *Hierarchical clustering heatmap representing the top 50 up- and 50 down-regulated genes (FDR <0.05, $log_2|FC|>0.25$) upon KLF6 overexpression in HPAECs. The coloured scale bar represents expression z-scores across the dataset.*

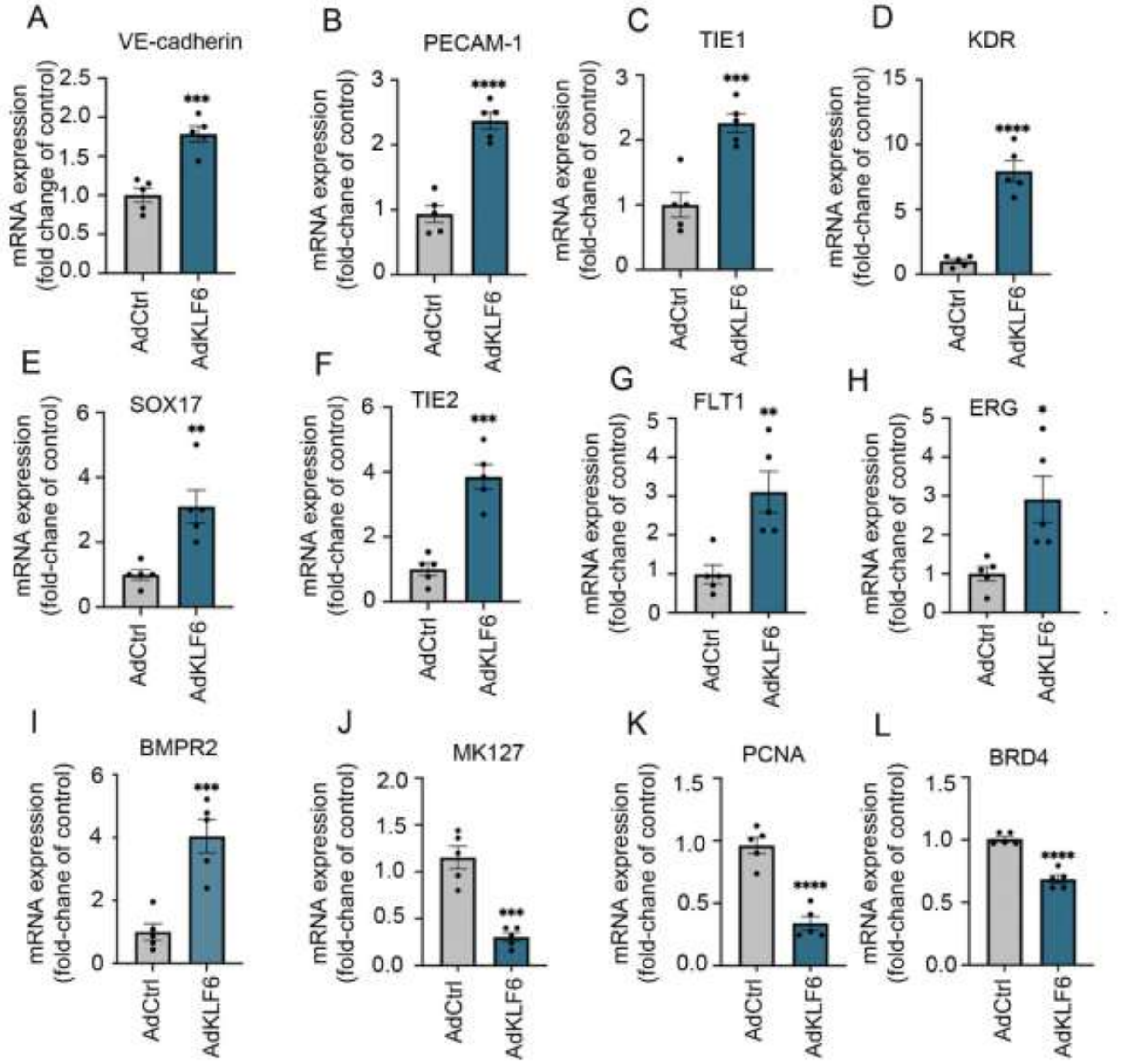

Supplementary Figure 6. Expression of selected KLF6-regulated genes.

*Gene expression of VE-cadherin, PECAM-1, TIE1, KDR, SOX17, TIE2, FLT1, ERG, BMPR2, MK127, PCNA and BRD4 was measured by qPCR and data were normalised to β-actin. N=5, *P<0.05, **P<0.01, ***P<o,001, ****P<0.0001, comparison with AdCTRL; unpaired Student t-test, n=5.*

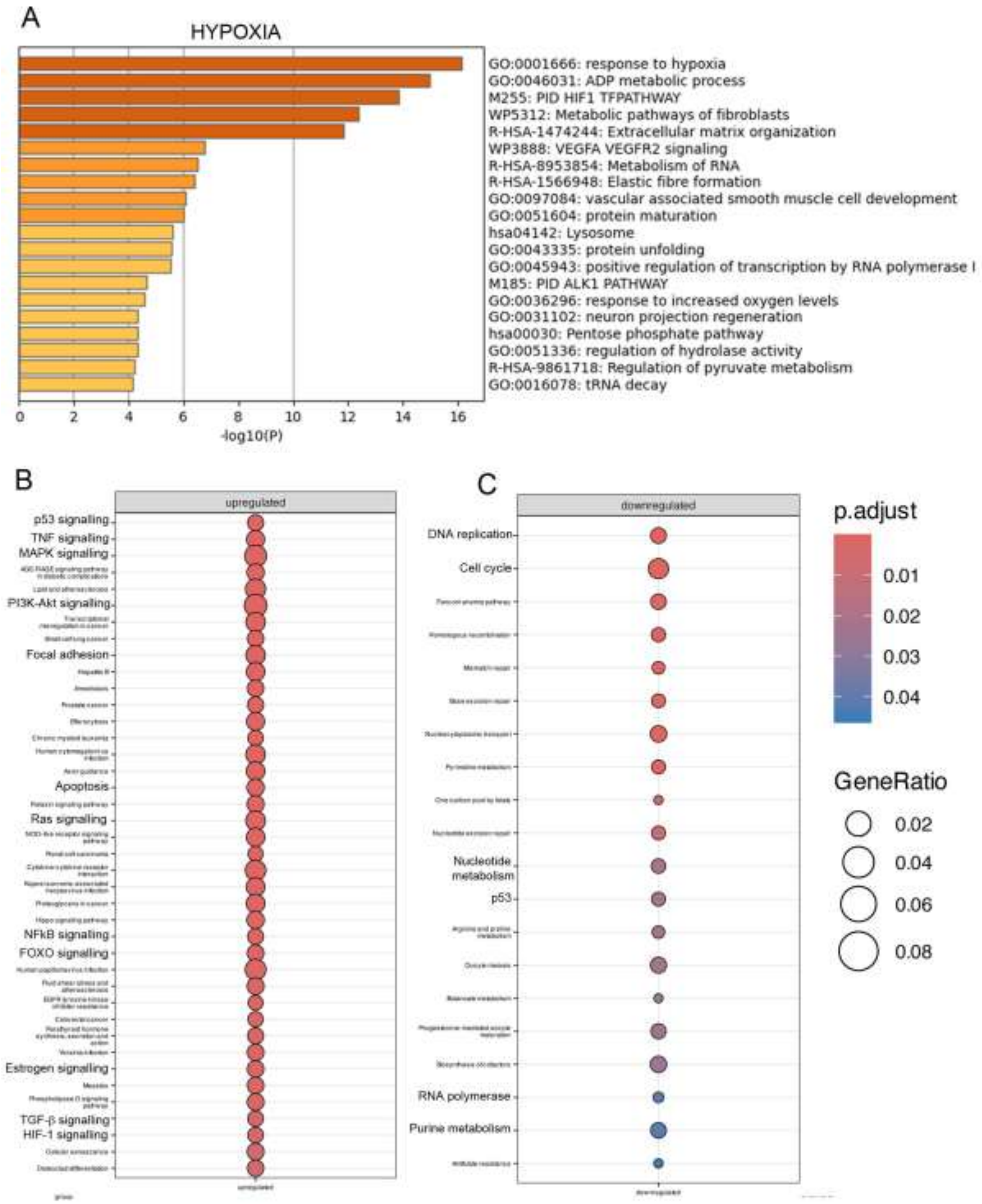

Supplementary Figure 7. Effect of KLF6 on hypoxia-induced changes in gene expression profile in HPAECs. ***(A)*** *Bar graph showing Metascape pathway and process enrichment analysis of DEGs following exposure of HPAECs to hypoxia (2% O2) for 24 hours. Bars are coloured by -log10 (p-value) and darker colour indicates more significant enrichment.* ***(B and C)*** *Dot plots showing the enriched KEGG pathways of (B) upregulated and (C) downregulated genes in KLF6 overexpressing HPAECs under hypoxic conditions. The colour of the dots represents the adjusted P-value, and the size of the dots represents the number of DEGs in the pathway.*

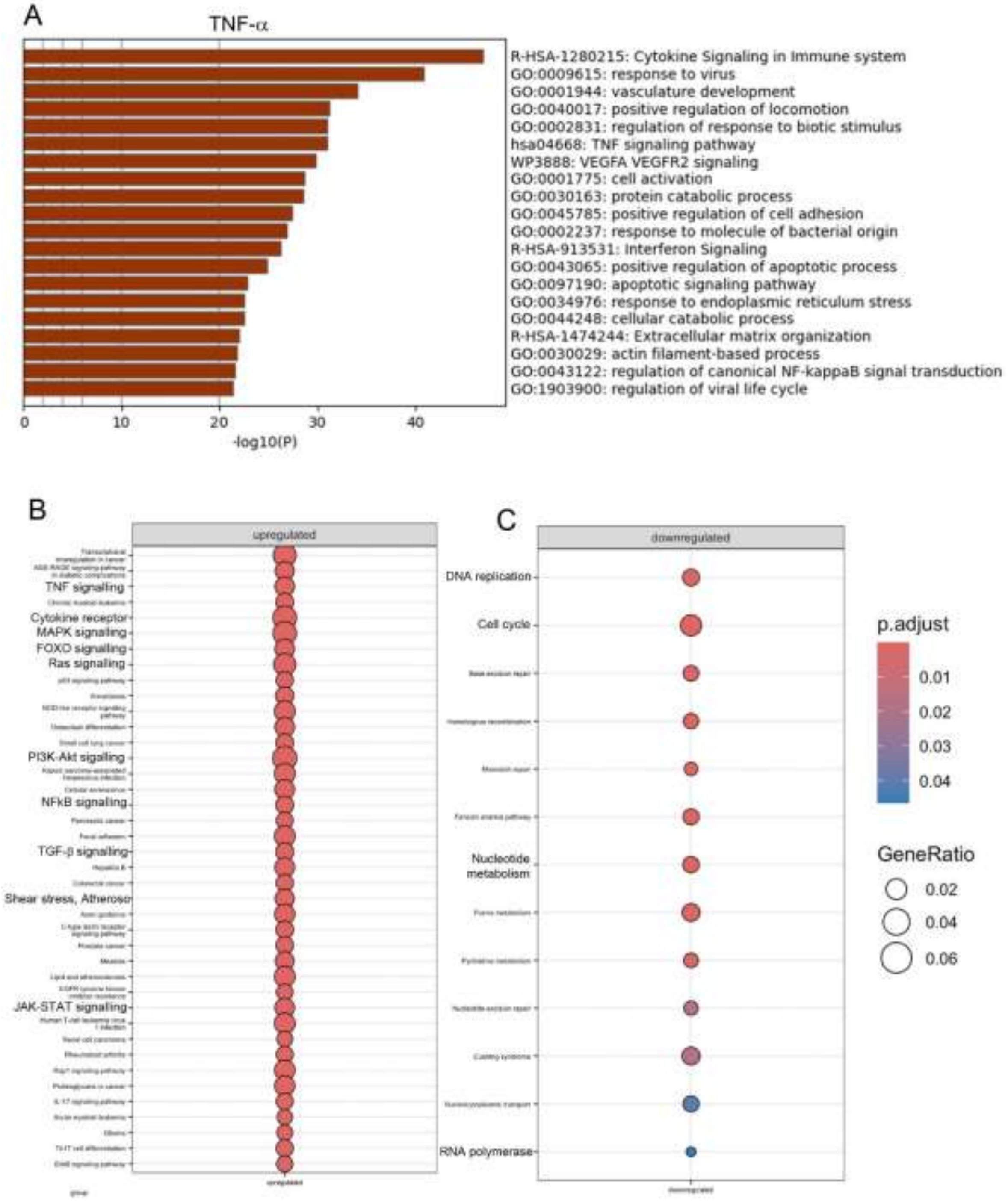

Supplementary Figure 8. Effect of KLF6 on TNF-a-induced changes in gene expression profile in HPAECs*. (A)** *Bar graph showing Metascape pathway and process enrichment analysis of DEGs following treatment of HPAECs with TNF-α (10 ng/ml) for 24 hours. Bars are coloured by -log10 (p-value) and darker colour indicates more significant enrichment.* ***(B, C) *Dot plots showing the enriched KEGG pathways of (B) upregulated and (C) downregulated genes in KLF6 overexpressing HPAECs treated with TNF-α. The colour of the dots represents the adjusted P-value, and the size of the dots represents the number of DEGs in the pathway.*

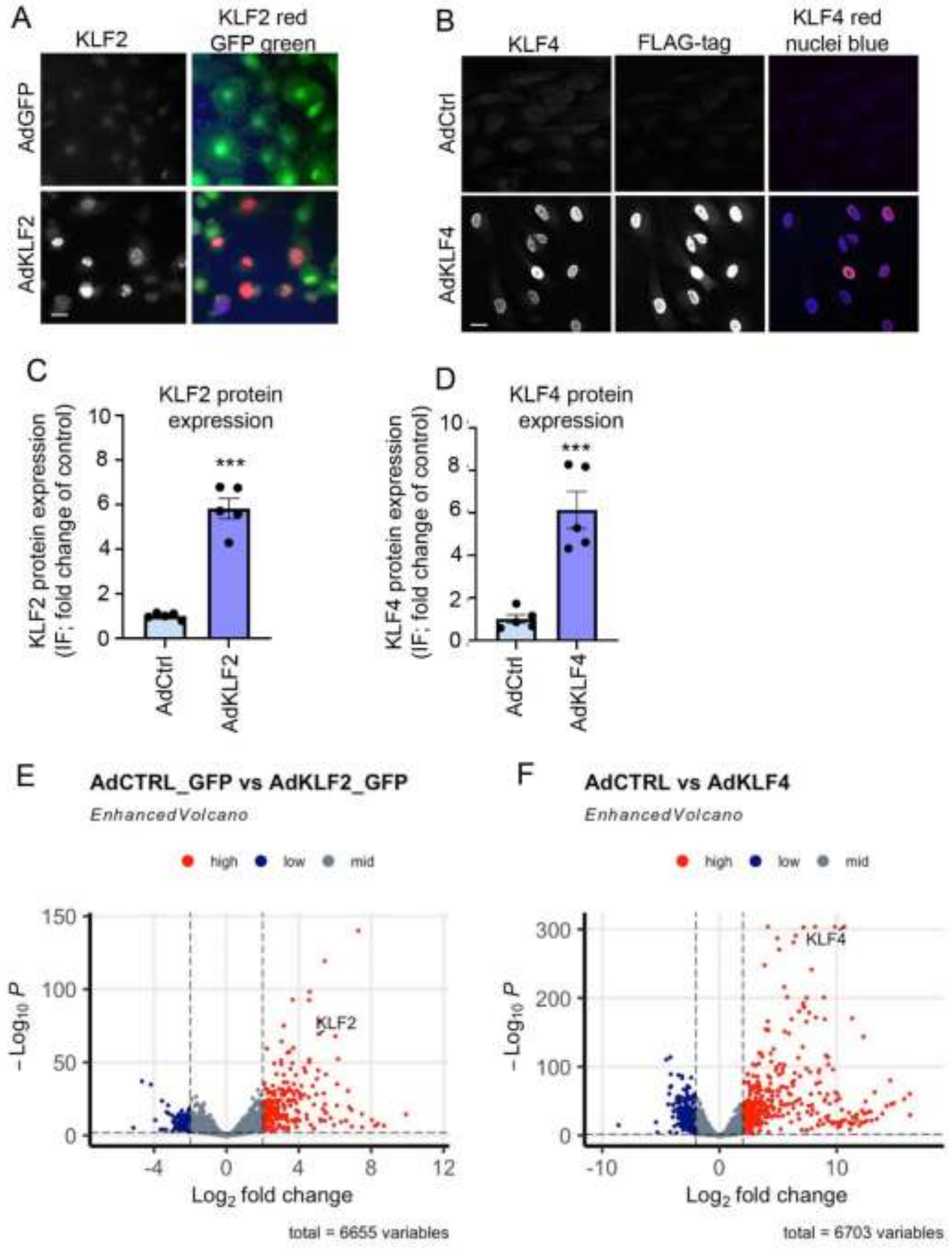

Supplementary Figure 9. Gene expression changes in HPAECs overexpressing KLF2 and KLF4. (A, B) *representative confocal microscopy images showing localization of (A) KLF2 and (B) KLF4 in HPAECs 24h post- infection with AdGFP (adenoviral control; AdCtrl), AdKLF2-GFP or AdKLF4-FLAG, as indicated. Bar=10µm.* ***(C, D)*** *show protein expression of (C) KLF2 and (D) KLF4 in HPAECs.* ***(E, F)*** *Volcano plots showing gene expression changes induced by overexpression of (E) KLF2 or (F) KLF4. Blue dots denote downregulated genes and red dots upregulated genes. Thresholds: P<0.05, 0.25-fold-change.*

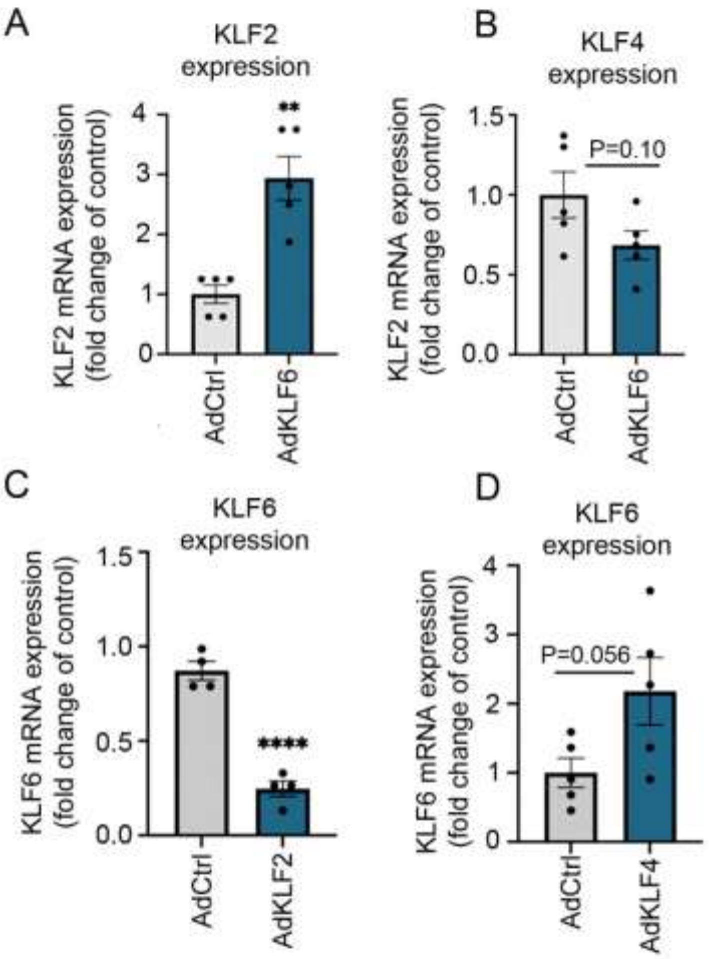

Supplementary Figure 10. Regulatory feedback relationships between KLF2, KLF4 and KLF6.

(A) *KLF2 mRNA and* ***(B)*** *KLF4 mRNA expression in HPAECs infected with AdCTRL or AdKLF6 for 24 h.* ***(C)*** *KLF6 mRNA expression in HPAECs infected with AdCTRL or AdKLF2 for 24 h.* ***(D)*** *KLF6 mRNA levels in cells infected with AdCTRL or AdKLF4 for 24 h, as indicated. In* ***(A-D)*** *data were normalized to the reference gene (β-Actin). **P<0.01; ****P<0.0001, comparison with adenoviral controls, unpaired t-test. Error bars indicate mean ±SEM; n=5.*

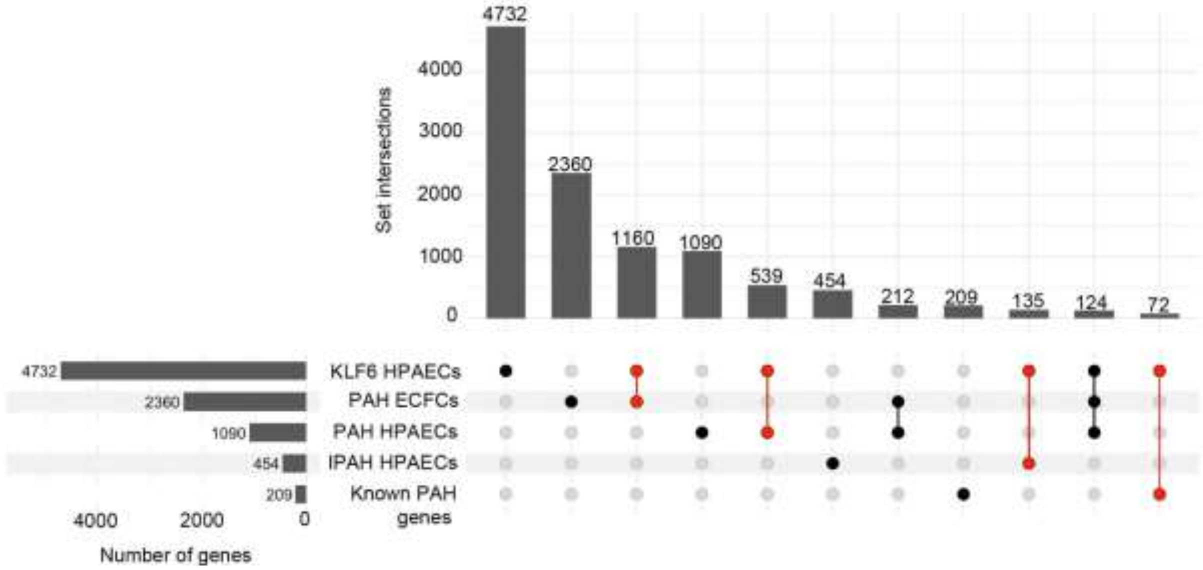

Supplementary Figure 11. Overlap of DEGs in KLF6 overexpressing HPAECs and PAH DEG databases. *Upset plot displaying the comparison between DEG lists of KLF6 HPAECs, PAH HPAECs, PAH ECFCs and IPAH PAECs, where connected black dots represent overlapping gene sets and vertical bars at the top show the number of overlapping genes in the respective overlap category. The size of the gene sets interrogated is shown as horizontal bars on the left side. Gene lists are provided in Supplementary Table S3.*

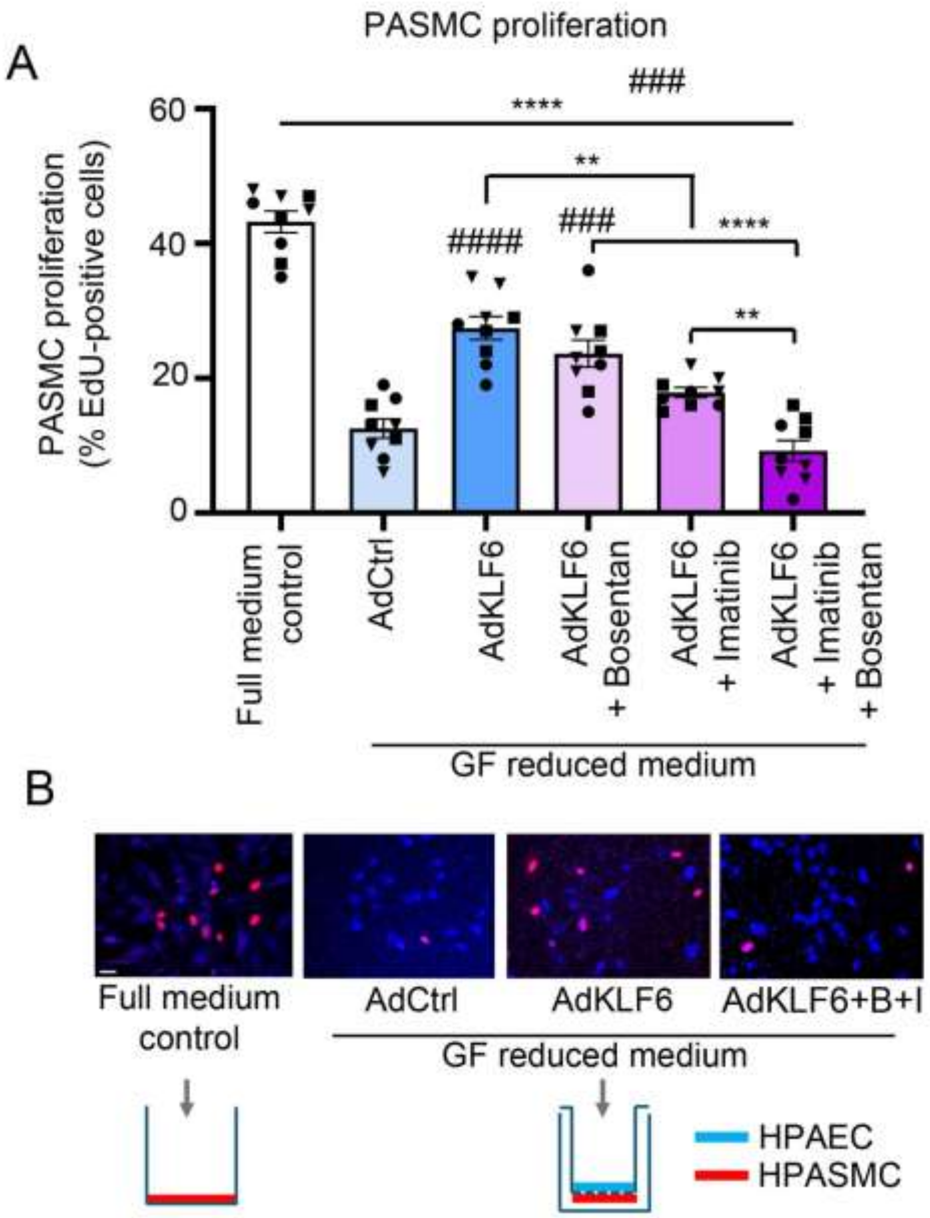

Supplementary Figure 12. Effect of endothelial KLF6 overexpression on HPASMC proliferation in vitro.

(A) *Graph showing proliferation of HPASMCs cultured alone or co-cultured with AdCTRL- or AdKLF6-treated HPAECs, as indicated; EdU incorporation assay. HPASMCs were cultured alone in optimal media (Full medium control) or were co-cultured with AdCTRL- or AdKLF6-overexpressing HPAECs in Transwell inserts, with the two cell types placed on either side of the porous membrane pore size 4µm in growth factor-depleted, serum-reduced (0.2% serum) medium for 16 hours. Bosentan (B; 20 µmol/L) and Imatinib (I; 10 µmol/L, were added to the cells separately, or in combination (B+I), as indicated. Error bars in (A) show means ±SEM; **P<0.01,***P<0.0001, ****P<0.0001, comparisons with full medium control, $^{###}$P<0.001, comparison, as indicated; one way ANOVA, n=9 (3 experimental repeats with 3 different biological donors ● Donor 1; ▼Donor 2; ■ Donor 3).* ***(B)*** *Corresponding representative fluorescent microscopy images of HPASMCs under different culture conditions, illustrated in diagrams below the images. Bar=10µm.*

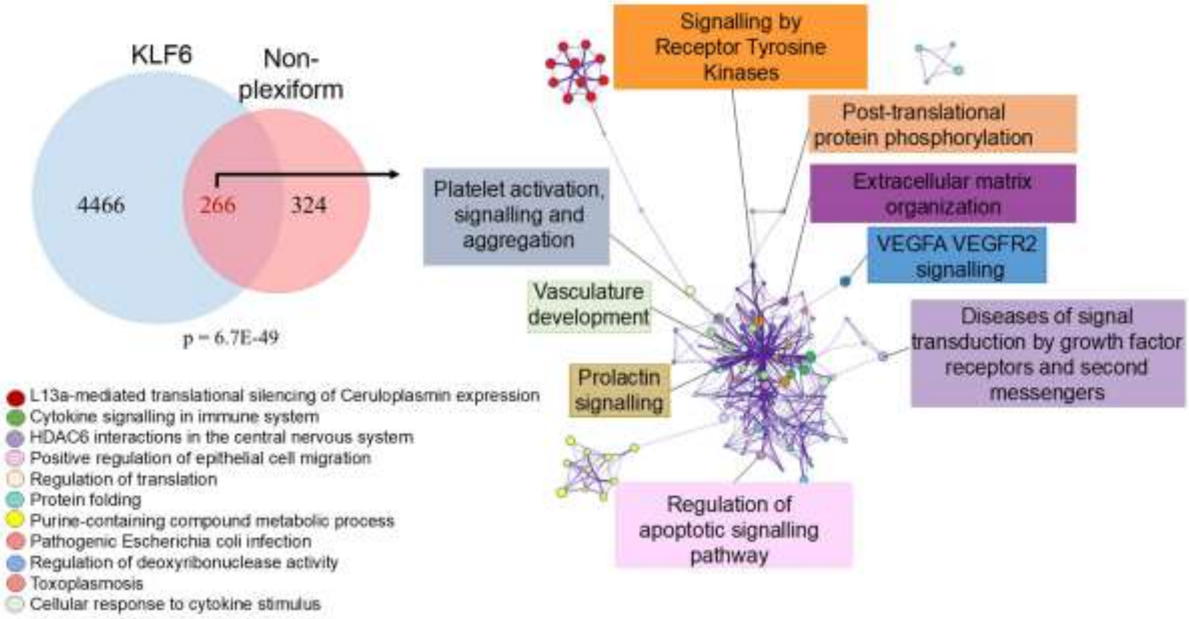

Supplementary Figure 13. KLF6-regulated DEGs in non-plexiform vascular lesions in PAH lung. *Venn diagram showing the overlap between KLF6-regulated DEGs in HPAECs and non-plexiform lesions DEGs identified by spatial transcriptomics; P= 6.7E-49, Fisher's exact test in the GeneOverlap package in R. The associated Metascape network of enriched terms in the shared pool of DEGs coloured by cluster, is shown on the right. FDR<0.05, log2|FC|>0.25.*

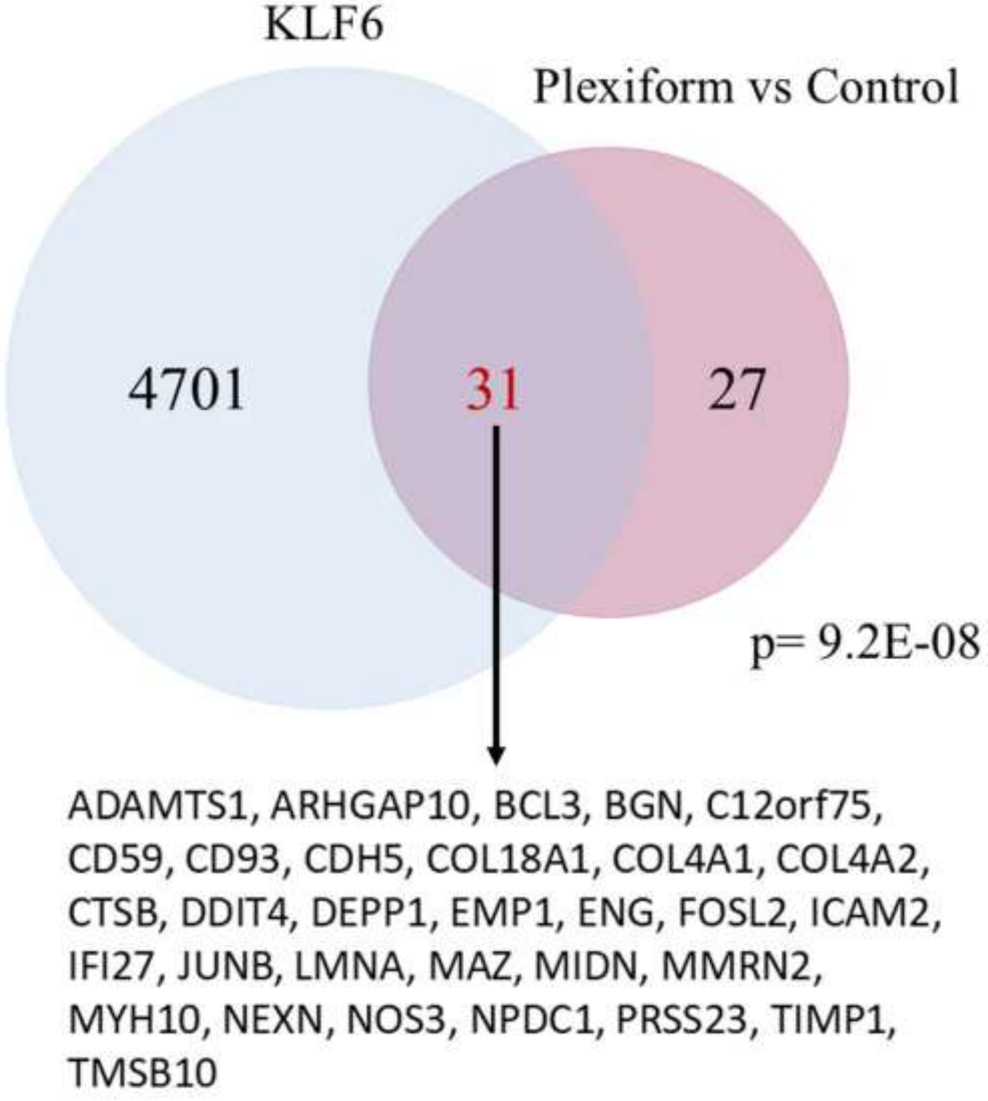

Supplementary Figure 14: Venn diagram showing an overlap between KLF6-regulated DEGs in HPAECs and plexiform lesion-specific DEGs in Tuder et al. (2024)*. The list of plexiform lesion-specific DEGs was taken from Table E6A (https://doi.org:10.1164/rccm.202307-1310OC). P= 9.2E-8, Fisher's exact test in the GeneOverlap package in R.*

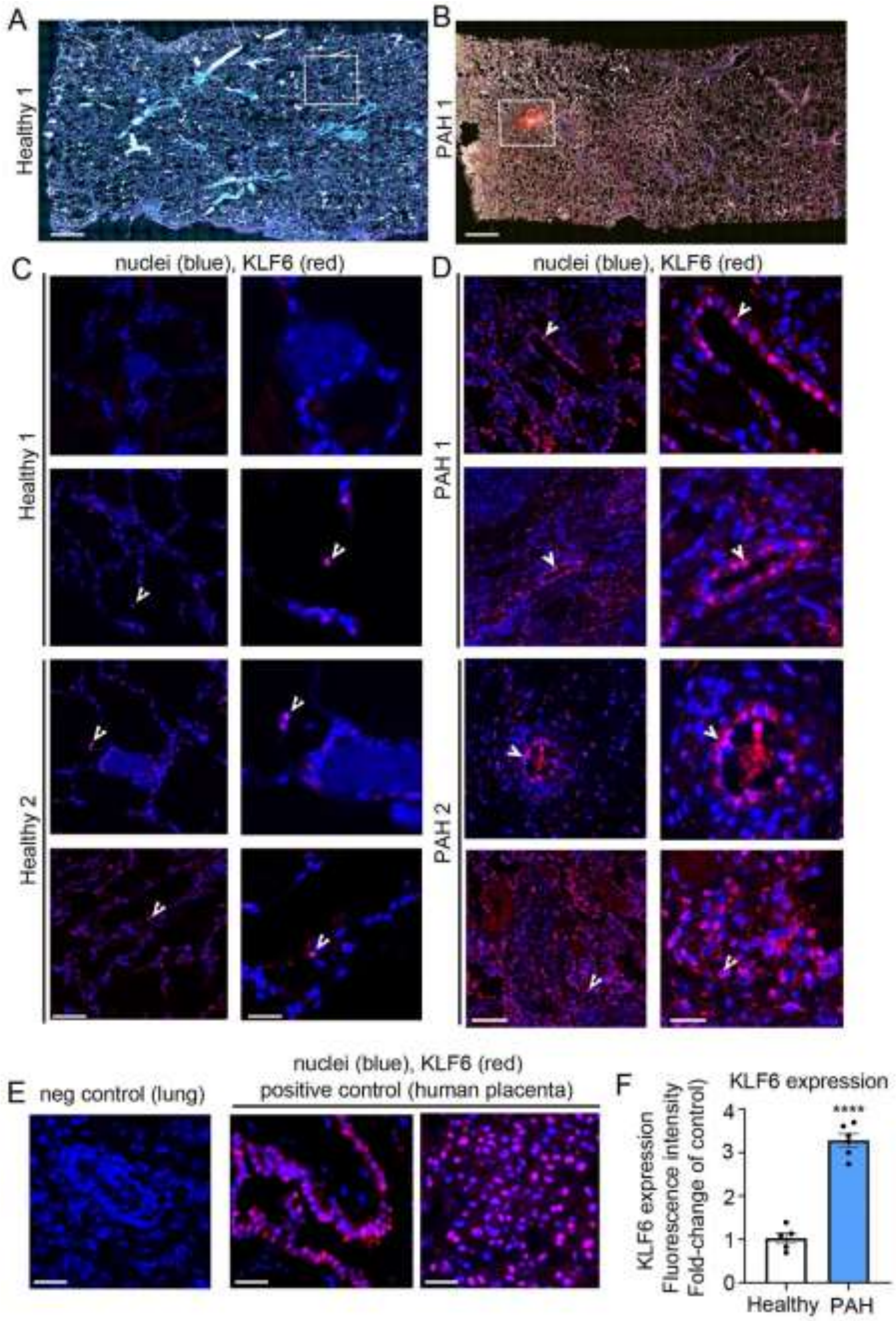

Supplementary Figure 15. Representative images of tissue sections used for spatial transcriptomic and KLF6 localization. (A) *Human healthy and* ***(B****) PAH lung, immunofluorescence: vWF (red), aSMA (yellow), nuclei (blue). Boxed areas indicate regions illustrated in (Healthy 1) and (PAH1) images below. Bar=1000µm.* ***(C)*** *KLF6 localization in healthy and* ***(D)*** *PAH lung tissues from 2 donors; immunofluorescence: KLF6(red) and nuclei (blue); Bar=50µm. Enlarged corresponding images are shown in the right panel; Bar=20µm Arrowheads point to nuclear localization of KLF6 (pink).* ***(E)*** *negative control (secondary antibody only) and positive control showing localization of KLF6 in human placenta, as indicated. Bar=50µm.* ***(F)*** *KLF6 expression PAH lungs (fluorescence intensity, fold-change of healthy control). Bars are means ± SEM; ****P<0.0001, comparison with healthy control; unpaired t-test n=5(healthy) and n=6 (PAH).*

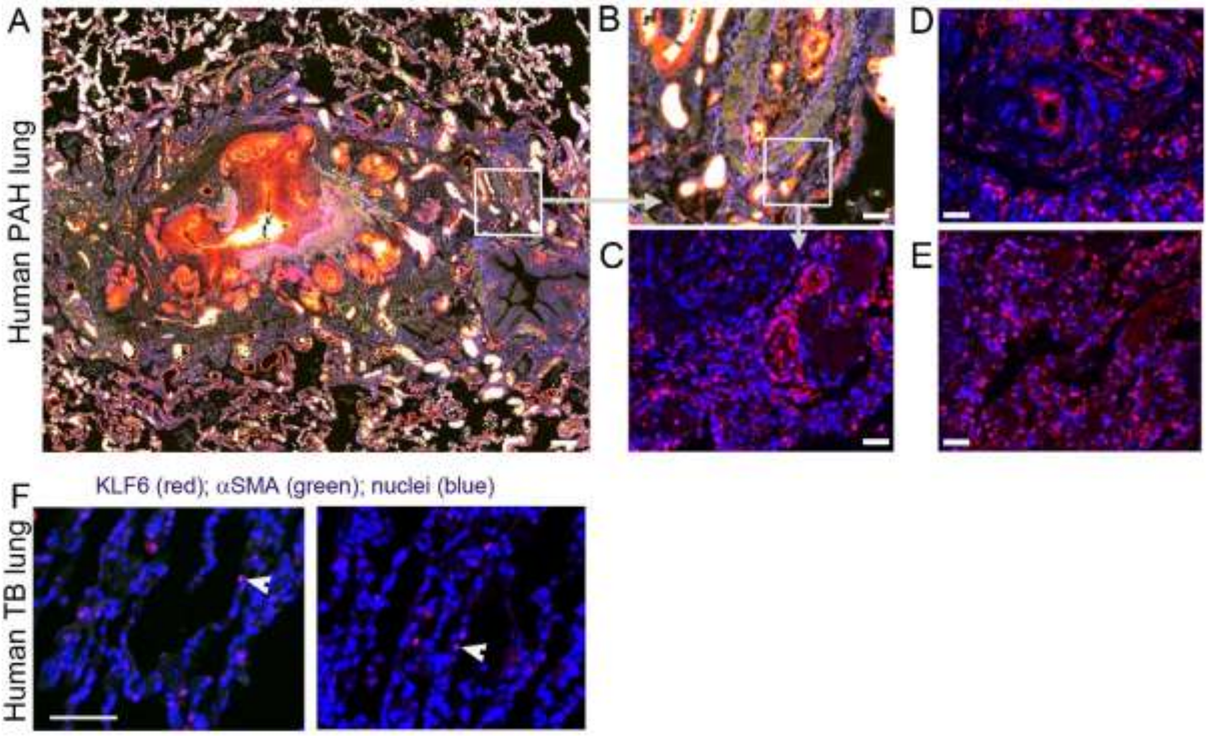

Supplementary Figure 16. KLF6 localization in human lung. (A-E) Immunostained tissue sections used for spatial transcriptomic and KLF6 localization in human PAH lung. (A) *Plexiform lesion;* ***(B****) Enlarged image of the boxed area from (A);* ***(C)*** *Enlarged image of vascular channels from the boxed area in (B).* ***(D, E)*** *Examples of KLF6 localization in the remodelled PAH lung, immunofluorescence;* ***(F)*** *KLF6 localization in the lung of patient with tuberculosis. Immunofluorescence, pseudocolours. In (A) and (B) vWF (red), aSMA (yellow), nuclei (blue); In (C, D, E, F) KLF6 (red) and nuclei (blue). In (A, F) Bar=100µm, in (B) Bar=50µm, in (C, D, E) Bar=20µm*

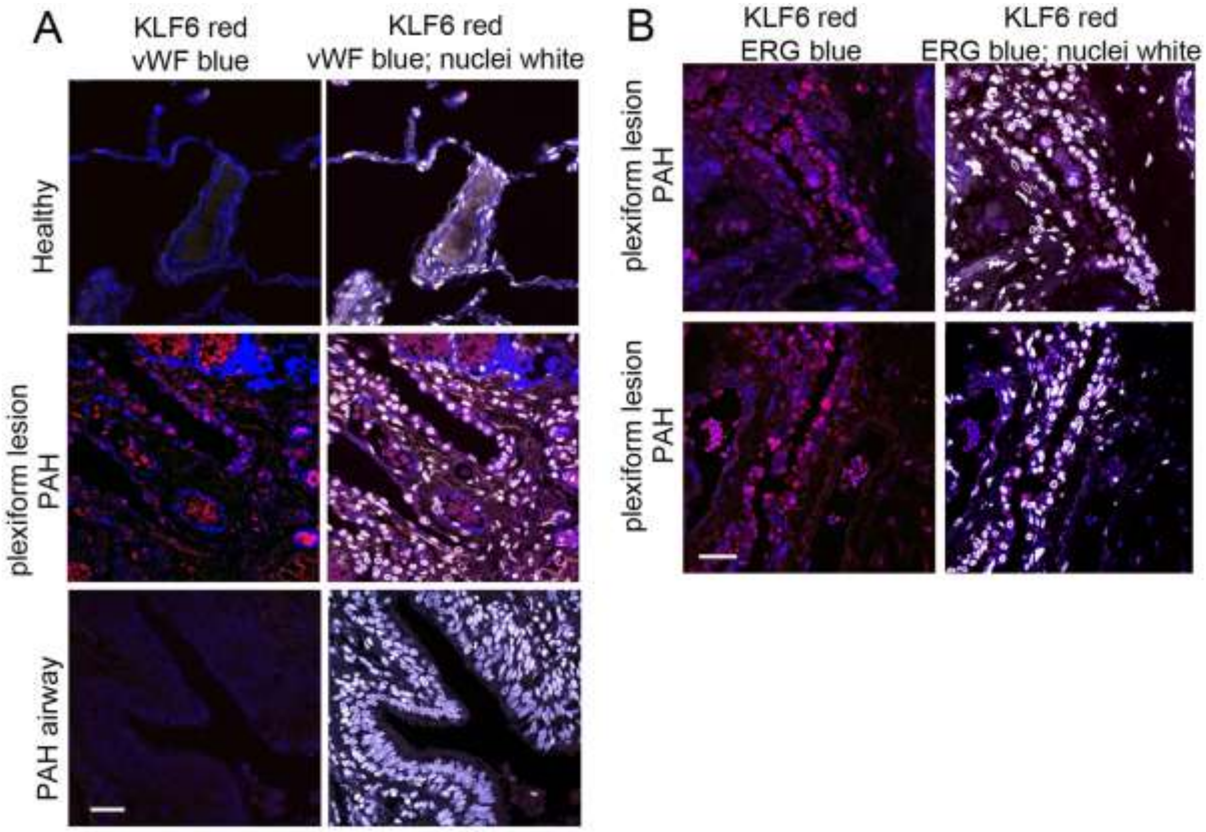

Supplementary Figure 17. KLF6 colocalization with endothelial markers Erg and vWF. (A) *vWF and* ***(B)*** *ERG immunostaining in healthy and PAH lung tissues, as indicated. KLF6 is red, vWF and Erg are blue and in merged images, nuclei are white. PAH tissues shown in panels A and B are part of a larger lesion illustrated in Figures S15B and S16A. Immunofluorescence, pseudocolours. Bar = 50µm.*

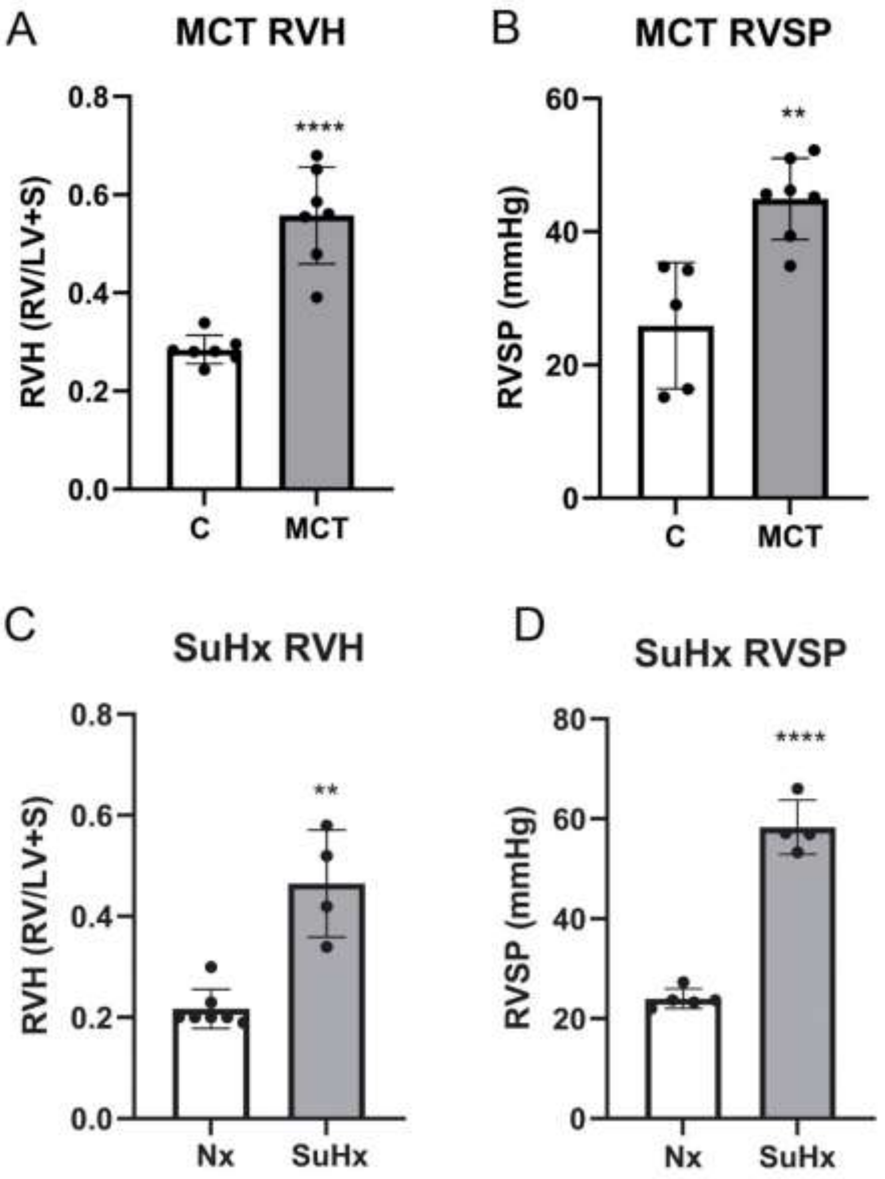

Supplementary Figure 18. Right ventricular hypertrophy (RVH) and Right Ventricular Systolic Pressure (RVSP) in Monocrotaline (MCT) and Sugen/hypoxia (SuHx) rats. *(A) RVH in MCT rats, (B) RVSP in MCT rats, (C) RVH in Su/Hx rats, (D) RVSP in SuHx rats. C-control, Nx-normoxic control, RV/LV+S – right ventricle/left ventricle+septum. Graphs are based on data published in [1]. Bars are means ± SEM. In (A, B) n=7, in (C, D) n=4-6. **P<0.01; ****P<0.0001, unpaired t-test, comparisons with healthy controls.*

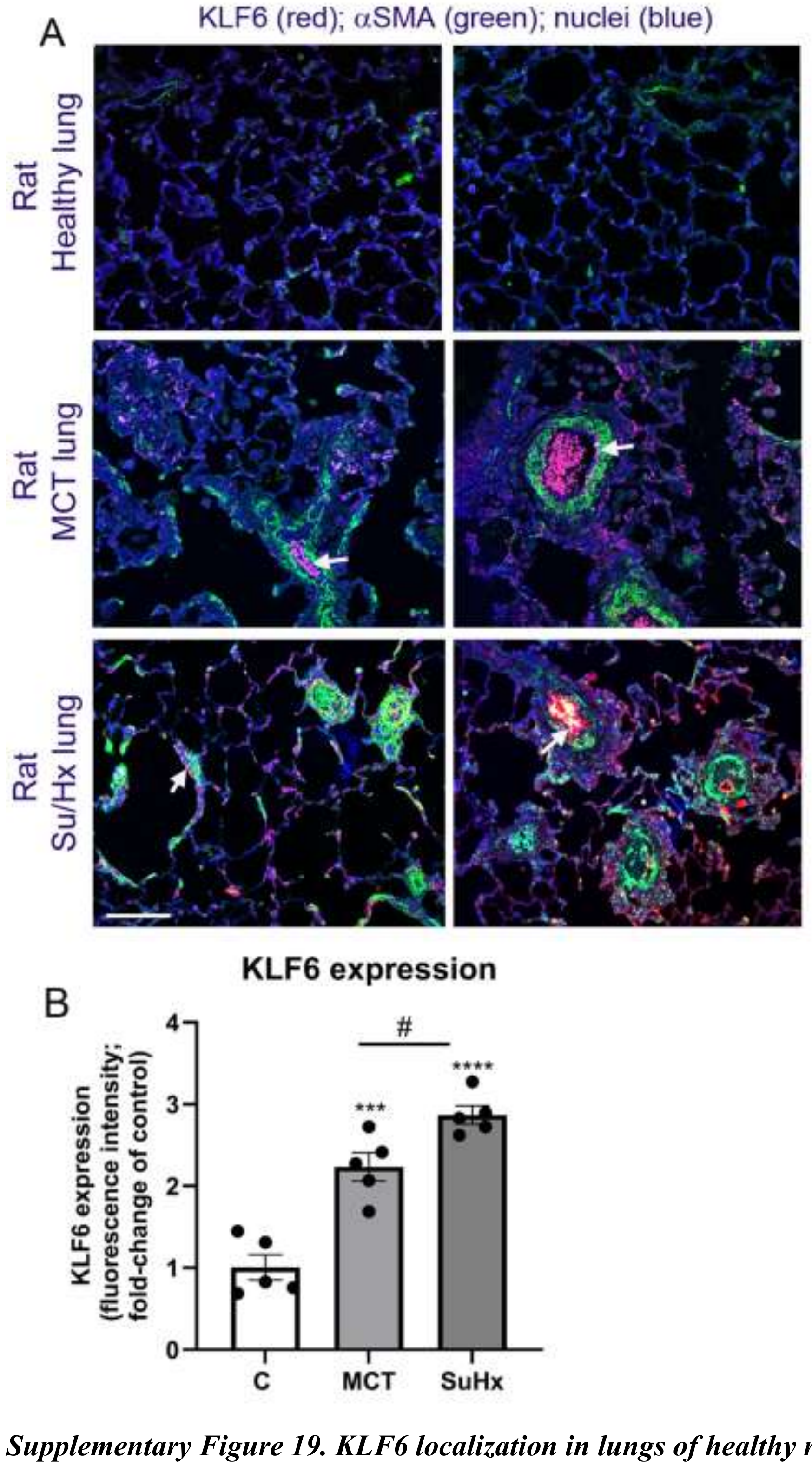

Supplementary Figure 19. KLF6 localization in lungs of healthy rats and rats with MCT and Sugen/hypoxia-induced PAH. *(A) Representative imunofluorescent images of KLF6 localization in healthy, MCT and Sugen/hypoxia (SuHx) rats, as indicated. KLF6 is red, α-SMA is green and nuclei are blue. Arrows point to nuclear localization of KLF6 (pink). Bar = 50μm. (B) KLF6 expression in MCT and SuHx rat lung tissues (fluorescence intensity, fold-change of healthy control).* Bars are means ± SEM*; ***P<0.001; ****P<0.0001,*

comparison with control; #P<0.05, comparison, as indicated; one-way ANOVA with Tukey post-test;

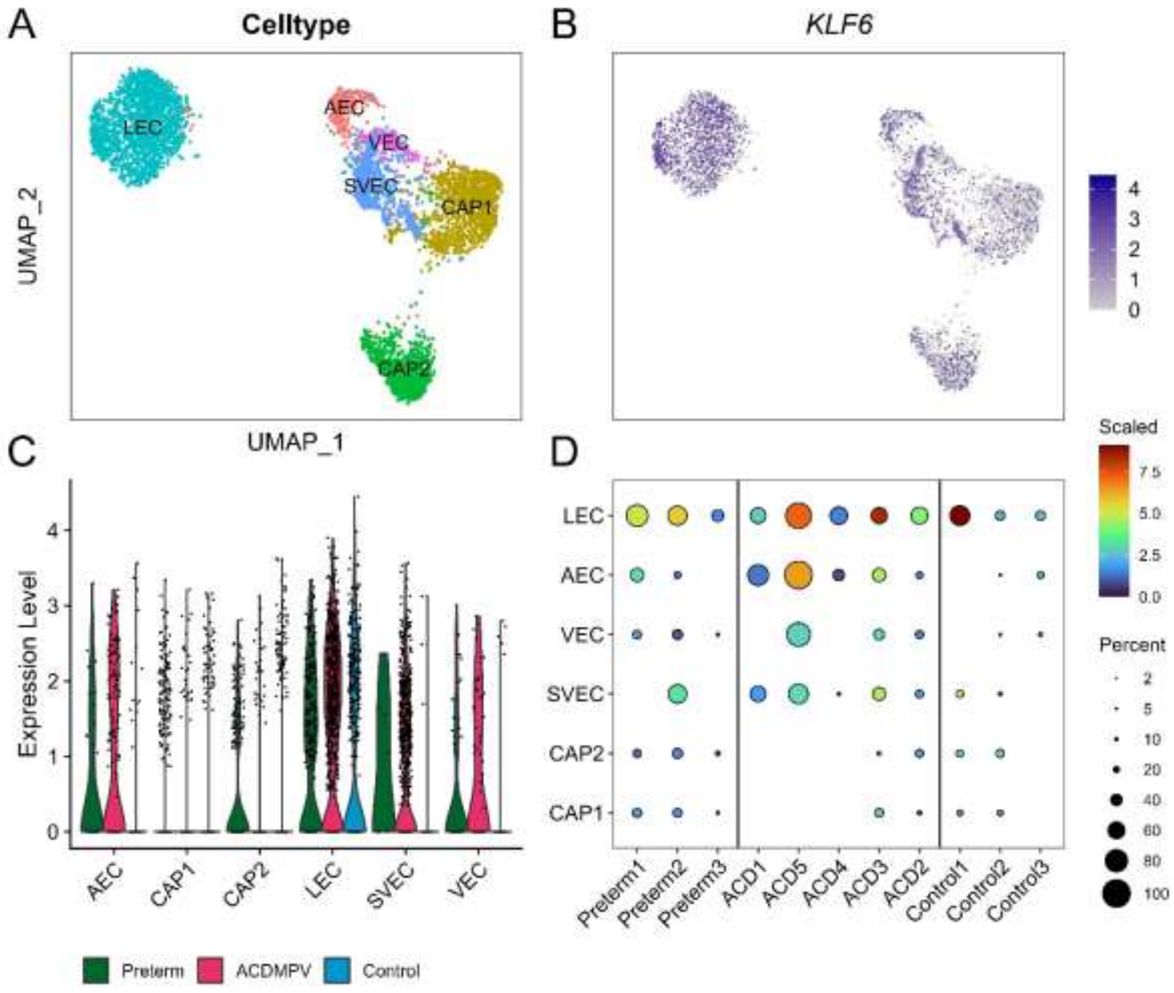

Supplementary Figure 20. Single-nucleus RNA-seq analysis of KLF6 expression in endothelial cells (ECs) in alveolar capillary dysplasia with misalignment of pulmonary veins (ACDMPV) and control human lungs. (A) *UMAP of identified EC subpopulations.* ***(B)*** *KLF6 expression in the identified EC populations. The level of KLF6 expression in each cell is shown by the intensity of blue colour. Cells with detectable KLF6 were plotted on top of cells without KLF6 expression (grey).* ***(C, D)*** *show quantitative evaluation of KLF6 expression in different cell subpopulations, as indicated: AECs are arterial endothelial cells, VEC are venous endothelial cells, LECs are lymphatic endothelial cells, SVECs are systemic vascular endothelial cells and CAP1 and CAP2 are capillary cells. Differential expression was performed using Seurat 4 FindMarkers function with Wilcoxon rank sum test. p<0.05, FC>=1.5, pct>20%.*

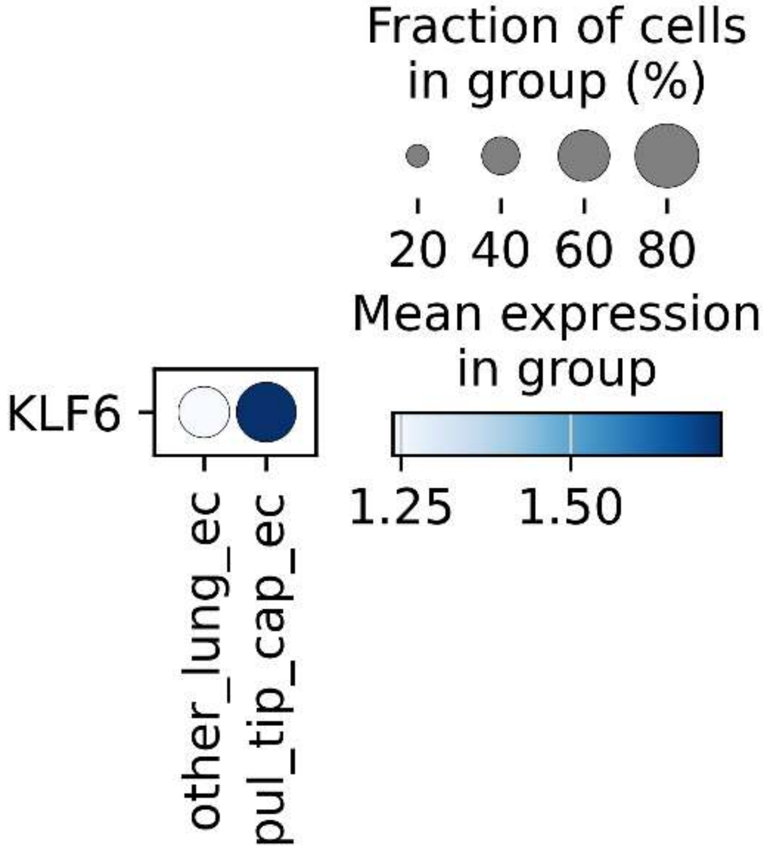

Supplementary Figure 21. *Dot plot representation of KLF6 expression in lung tip endothelial cells (pul_tip_cap_ec) compared to other merged lung blood vessel endothelial cell populations (other_lung_ec) using single-cell RNA-seq data acquired from Barnett et al. 2024 Nature Medicine*[2].

SUPPLEMENTARY TABLES

Supplementary Table 1: List of unique DEGs for KLF2, KLF4 and KLF6.

Name	Unique genes
KLF2	*ENSG00000196274, RN7SL718P, RN7SL670P, KLF2, ICAM1, ENSG00000237611, ENSG00000267834, RN7SL455P, RN7SL602P, ENSG00000210100, ENSG00000278903, MIR3648-1, EZH2, CABLES2, MIR663AHG, HSPA14, GINS3, TRAF3, MSH6, ACD, FAM210A, ENSG00000234160, SCML1, ENSG00000280800, SNHG1, PRC1, USP36, LOC122526782, SRSF7, TOP3A, NCAPD3, TEX30, RN7SL3, IMMP1L, PHGDH, ENSG00000255198, HLA-DPB1, CCDC14, TFAM, RFLNB, RRP12, RANBP1, FAM124B, SUOX, TRIM69, MX2, HHIP, TFPI, IFI6, OAS2, TNIK, NRP1, XAF1, TRIM5, ISG15, GRAP, GIMAP8, RIGI, MX1, ERG, AMIGO2, STAT2, EPSTI1, MARCKS, LYL1, ZNF561, RNF144B, IFI35, RSAD2, TM4SF1, CASP1, ENC1, DUSP6, EFNA1, JUP, SAMD9L, CD93, EDN1, IFI44L, ENSG00000289833, TP53I11, FAM43A, STAT1, IFITM1, ALDH1A1, LOC102724951, SCHIP1, CLIC1, CXCL11, CXCL10*
KLF4	*SEMA3F, LASP1, ATOSB, ST3GAL1, IL32, PLAUR, DNASE1L1, SERPINB1, OSBPL5, TIMP2, VCAN, RRM2B, NEDD4L, ATP2B4, SLC2A3, TNK2, GPC1, DLX3, DGKA, SLC9A3, ELOVL1, RASGRP2, PPP2R5B, BCL3, FSTL3, GBA2, STK10, ATP2A3, MGLL, FSCN1, NMRK2, LRCH4, TP53INP2, TNS1, CHRNA3, BCORL1, WNT11, PTHLH, PPP1R13B, SMOX, SLC8B1, RCOR1, CHRD, UNC13D, TBC1D2, CBARP, PALM, MKNK2, SH3BP1, SUN2, APOL1, GSKIP, CPNE6, ELF4, TIMP1, MEDAG, MT4, MMP15, NDRG4, CEMIP, NDRG1, KCNN4, TUBB4A, CKM, DMPK, SNAPC2, EBI3, CNFN, CYTH2, CLEC11A, RASA4, LFNG, TESK1, CREB3, VSIR, SLC9A3R1, CDR2L, PMP22, ALDOC, KLF3, UGDH, SLC15A3, CD81, PITPNM1, KRT18, ART4, MGP, RAB5B, VEGFA, HES1, TNNC1, CLCN2, IL1RL1, GBP3, VAMP8, PGF, FKBP1B, TEK, FABP3, GLIPR2, DNPEP, SNAI1, PMEPA1, GRM4, LRRFIP1, WNT1, MT1G, DOK4, IRF1, TNFSF9, C3, ID1, SLURP1, ARMCX1, EDN2, FBXL12, APOL2, RAC2, FLNC, LOXL1, KLK14, ADCY4, CDKN1C, TNNI3, HELZ2, H19, CALY, ULBP2, EPS8L1, ACSS2, NFATC1, MAP1B, RARA, DNAJB1, TRIM21, CARD6, HSPA12B, CASP9, CCNA1, PLAAT4, IRAK2, BHLHE40, BIN1, KLF4, TUBB2A, IER3, IL18BP, LRRC32, SQOR, DUSP5, SHF, CERS5, RHOF, BAHD1, PSTPIP1, OSGIN1, CBX4, STAC2, COL6A1, COL6A2, ATP1B1, EFNA3, CSRNP1, SLC16A2, SLC25A25, LCN2, ADIRF, TNKS1BP1, TAGLN, HSPB8, ZFP36L2, CHD1, TRIM11, RABGEF1, ADAMTS1, KLF10, PCDH1, MYO1E, TMEM164, MRAS, CDA, SPON2, ZBTB7B, LMNA, SQSTM1, SCGB3A1, PLCD3, TAMALIN, BCL6B, HS3ST6, FGF19, LAPTM5, SYNC, KIAA1522, CAMK2N1, ATF3, IER5, IHH, MELTF, DUSP7, CSF2, MICALL2, TSC22D4, GOLGA2, CA4, MIDN, SERTAD3, PSCA, LY6D, RILP, KLK1, KLK13, SPRYD3, NSG1, LGALS9, ASMTL, NPR1, FASN, KLF13, CST1, KRT8, KISS1, PFKFB3, SERPINB9, HTRA3, SOX7, BBLN, JUNB, KRT19, KRT13, CXXC5, ZNF274, SPSB1, MAL, ORMDL3, LRRC15, ISG20, CEBPB, PRSS27, RAB43, OVOL1, RARG, HEG1, RNF213, PHLDA3, SLCO2A1, DES, MARCKSL1, DDIT3, TBC1D10C, DRAP1, BAIAP2, TPRN, LPCAT4, EPS8L2, ULK1, CASKIN2, GPR4, PNPLA2, CRACR2B, MLF1, ZFAND2A, PARP10, THBD, ZBTB7A, CDC42EP4, PJA1, PHLDA2, ADGRB1, FBXL6, TSKU, AFMID, SMTN, OAF, ALDH1A3, PTP4A3, SOCS3, TRARG1, DGAT1, MAFF, SIGIRR, IL3RA, IRF7, AHNAK2, ARAP1, PDE2A, PRR5, VSIG10L, KRT16, HEXIM1, KRT14, TRABD2A, ESPN, PLCD1, MT1X, BCAM, PEAR1, S100A3, FAM25A, FAM83G, SBSN, FAM25G, CLDN4, S100A4, HRCT1, S100A2, LAMB3, PDLIM7, SERTAD1, LYPD2, STMN3, EOLA1, SLC22A20P, AKAP17A, TPM2, HSPA1B, PSORS1C2, CDSN, C6orf15, AKT1S1, ENSG00000204791, ADGRG1, MT1M, KRT6A, RNU6-36P, RNU6-33P, GPX3, ACKR1, LTC4S, LCAT, DDAH2, PPM1N, NPTXR, PLEKHM1, CBLL1-AS1, PLEKHO2, PEG10, TNFRSF6B, GPR162, LINC00520, WFDC21P, SCRT1, LIN37, SLURP2, ENSG00000289316, ENSG00000291309, SCMH1, POLA2, BID, MRTO4, MBD3, TIPIN, SPAG5, UBE2T, CAD, BIRC5, NAT14, VRK1, NME4, BCAT2, SNX8, NCS1, NPM3, CCDC34, PTPMT1,*

	FOXM1, OGG1, GPX7, POLR1G, DYNC2I2, CLCC1, CSE1L, PDCD2L, BCL2L12, SRD5A3, PAICS, DUT, GAMT, EXOSC2, SCLY, PCNA, NASP, ADCK2, LPIN1, DZIP1, CDK4, RNASEH2B, POLR1E, SLC37A4, ATIC, HERC5, GCSH, ARRB2, NFIC, PARP1, GINS4, SIGMAR1, GBGT1, B3GAT3, GXYLT1, SPC25, ADGRA3, AGPAT5, NDUFAF6, CIP2A, CXCL5, RPL39L, CDCA7L, FABP5, COMTD1, PACSIN3, CLMP, C19orf48, PBK, DTYMK, CDK1, FABP4, SPTBN2, SPHK1, PFAS, AURKB, RCC1, IDH2, SFXN4, WDR5, IPO4, TFDP1, JPT2, STIMATE, BOP1
KLF6	*DCBLD2, KLF6, PIR, PALMD, CAV1, SMURF2, SULT1E1, NEDD9, PLSCR4, ST3GAL5, RND3, GADD45A, PIK3R3, TNFSF18, FAM117A, CDKN1A, CXCL6, LRRC17, AJUBA, RNASE1, ITGB4, SLC38A2, MDM2, RAPGEF5, IL33, BCAR3, HECW2, ARHGAP24, PRICKLE1, ACVRL1, LPAR6, CCN1, MALL, HMGA2, ROBO4, LY96, TSC22D3, ETS2, BTG2, VCAM1, SOX17, MAMDC2, MTMR10, CXCL8, ZMAT3, CD34, C11orf68, PRR15, CITED4, FAM167B, ZFP36L1, KANK3, PDCD1LG2, LGR4, ENSG00000233818, LINC00607, RPL32P29, MIR100HG, ENSG00000256664, LNCOG, TXNIP, NCAPH2, MTHFD1, IL27RA, FBRSL1, H2BC11, XRCC3, TUBA4A, TOMM40, HAUS2, DNAJA4, SLC39A4, PDSS1, MKI67, SLC43A1, PUSL1, MCRIP2, SHMT1, POLE, SLC25A22, SEPTIN5, TOR3A, H2AX, ATAD3A, EIF4EBP3, EEF1AKMT4*

Supplementary Table 2: List of differentially expressed genes shared between KLF2, KLF4 and KLF6.

All have gene symbols or Ensemble ID.

Comparison List	Overlapping genes
KLF2, KLF4 and KLF6	*ANGPTL4, APOE, ASF1B, ATAD2, ATAD3B, BRME1, CCNE1, CDC25A, CDC45, CDC6, CDCA5, CDK2, CENPK, CENPM, CENPQ, CENPU, CENPV, CHAF1B, CKB, CLDN5, CLSPN, CRABP2, CX3CL1, DERL3, DNMT1, DSCC1, DSP, DTL, DUSP2, DUSP9, EEF1A2, EMP2, ENSG00000259316, ENSG00000277605, ENSG00000279573, ENSG00000290032, ENSG00000291224, ERFE, ESCO2, EXOSC5, FAM111B, FANCI, FEN1, FGFR3, FOXRED2, FRMD6, FSD1, GINS2, H2BC12, HADH, HELLS, IL6, KHK, LINC01551, LINGO1, LRP3, MAD2L1, MCM2, MCM3, MCM4, MCM5, MCM6, MCM7, MCM8, MIR4300HG, MSH2, MT1A, MYBL2, MYO19, MYRF, NAT8L, NETO2, NPTX1, NR2C2AP, OLFM2, P2RX5, PCLAF, PDGFB, POLA1, PRRT4, PSMC3IP, RAD51, RAD51AP1, RBBP8, RECQL4, RFC2, RFC3, RFC4, RFC5, RRM1, RRM2, SDC1, SLC25A19, SLC47A1, TEDC2, TMEM106C, TONSL, TYMS, UHRF1, UNG, VASN, WDR76, ZNF367, ZWINT*
KLF2 and KLF4	*ANGPTL4, APOE, AQP1, AQP3, ARC, ASF1B, ASS1, ATAD2, ATAD3B, BCYRN1, BRME1, C11orf96, CA2, CCNE1, CD52, CD55, CDC25A, CDC45, CDC6, CDCA5, CDK2, CENPK, CENPM, CENPQ, CENPU, CENPV, CHAF1B, CKB, CLDN5, CLIC3, CLSPN, COL1A1, COL2A1, COL9A3, COMP, CRABP2, CRIP1, CRLF1, CSPG4, CX3CL1, DEPP1, DERL3, DNMT1, DSCC1, DSP, DTL, DUSP2, DUSP9, ECM1, EEF1A2, EMP2, ENSG00000259316, ENSG00000277605, ENSG00000279573, ENSG00000282993, ENSG00000290032, ENSG00000291224, ERFE, ESCO2, EXOSC5, FAM111B, FANCI, FEN1, FGFR3, FOXRED2, FRMD6, FSD1, GFPT2, GIMAP6, GINS2, H2BC12, HADH, HBA1, HBA2, HELLS, IFIT1, IGF2, IGFBP6, IL11, IL6, KHK, KISS1R, KLK10,*

	LCN6, LINC01551, LINGO1, LRP3, MAD2L1, MCM2, MCM3, MCM4, MCM5, MCM6, MCM7, MCM8, MIR4300HG, MSH2, MT1A, MYBL2, MYO19, MYRF, NAT8L, NETO2, NGEF, NGFR, NOS3, NPPC, NPTX1, NR2C2AP, OLFM2, P2RX5, PCLAF, PDGFB, PI16, PLVAP, POLA1, PRRT4, PSMC3IP, PTGDS, RAD51, RAD51AP1, RBBP8, RECQL4, RFC2, RFC3, RFC4, RFC5, RRAD, RRM1, RRM2, S100P, SCNN1D, SDC1, SLC17A7, SLC25A19, SLC47A1, SLC6A8, SOX8, TEDC2, TMEM106C, TNFAIP2, TNNT1, TNXB, TONSL, TRH, TYMS, UHRF1, ULBP1, UNG, VASN, WDR76, ZNF367, ZWINT
KLF2 and KLF6	*ACKR4, ADAMTS18, ADCY3, AK4, ANGPTL4, ANKRD50, APLN, APOC1, APOE, ARHGAP18, ARRDC3, ASF1B, ATAD2, ATAD3B, BBC3, BMP4, BMX, BRME1, BTG3, CALCRL, CCDC80, CCL2, CCNE1, CCNE2, CD274, CD83, CDC25A, CDC45, CDC6, CDCA5, CDK2, CDT1, CENPK, CENPM, CENPQ, CENPU, CENPV, CERS1, CHAF1A, CHAF1B, CHRNA5, CKB, CLDN11, CLDN5, CLEC14A, CLSPN, CRABP2, CSRP2, CX3CL1, DDIT4, DERL3, DNAJB4, DNMT1, DONSON, DSCC1, DSP, DTL, DUSP2, DUSP9, E2F1, EEF1A2, EMCN, EMP2, ENO2, ENSG00000259316, ENSG00000267102, ENSG00000267583, ENSG00000277605, ENSG00000279337, ENSG00000279573, ENSG00000290032, ENSG00000291224, ERFE, ESCO2, EXOSC5, FAM111B, FANCA, FANCI, FEN1, FGFR3, FGFRL1, FILIP1L, FLRT2, FOXRED2, FRMD6, FSD1, GAL, GASK1B, GBP2, GIMAP2, GIMAP7, GINS2, GJA5, GMNN, H2BC12, HADH, HELLS, HES4, IL6, INKA1, KDR, KHK, LDB2, LINC01013, LINC01235, LINC01551, LINGO1, LIPG, LRP3, LRRC70, LYPD1, MAD2L1, MCM2, MCM3, MCM4, MCM5, MCM6, MCM7, MCM8, MIR4300HG, MSH2, MT1A, MTHFD2, MYBL2, MYO19, MYRF, NAT8L, NETO2, NPTX1, NR2C2AP, NR2F2, NREP, OLFM2, ORC6, P2RX5, PAQR4, PCLAF, PDGFB, PDXP, PHLDA1, PKMYT1, PLK2, POLA1, POLD3, PPIF, PRPS2, PRRG1, PRRT4, PSMC3IP, PTX3, RAD51, RAD51AP1, RBBP8, RECQL4, RFC2, RFC3, RFC4, RFC5, RGS3, RRM1, RRM2, SAMD1, SDC1, SH2D3C, SLC25A19, SLC39A8, SLC43A3, SLC47A1, SLC7A5, SLCO4A1, SMCO4, SOX4, SPAAR, SPRY2, TCF19, TCF7, TCIM, TEDC2, TGFB2, TGFBR2, THBS1, TK1, TM4SF18, TMEM106C, TMEM140, TMEM201, TMEM38B, TNFSF10, TNFSF4, TONSL, TSEN54, TUBGCP4, TYMS, TYRO3, UHRF1, UNG, VASN, WDR76, ZMYND8, ZNF189, ZNF367, ZNF521, ZSCAN31, ZWINT*
KLF4 and KLF6	*ACKR3, ADAMTS4, ADAMTS9, ALKBH2, ANGPTL4, APOE, AQP5, ASF1B, ASNS, ATAD2, ATAD3B, BCL2L1, BRME1, C1QTNF6, CCNE1, CD82, CDC25A, CDC45, CDC6, CDCA5, CDK2, CENPH, CENPK, CENPM, CENPQ, CENPU, CENPV, CEP78, CHAF1B, CITED2, CKB, CLDN14, CLDN5, CLN6, CLSPN, CMSS1, CRABP2, CX3CL1, CXCL1, CXCL2, DERL3, DHFR, DNMT1, DPH2, DSCC1, DSP, DTL, DUSP2, DUSP9, EEF1A2, EFNB1, EMP2, ENSG00000188985, ENSG00000210049, ENSG00000256514, ENSG00000259316, ENSG00000273420, ENSG00000277605, ENSG00000279573, ENSG00000290032, ENSG00000291224, ERFE, ESCO2, EXOSC5, FAM107A, FAM111B, FAM216A, FANCG, FANCI, FEN1, FGFR3, FOS, FOSL2, FOXRED2, FRMD6, FSD1, GDF15, GINS2, H2BC12, H4C14, HADH, HAGHL, HELLS, HSPA6, HSPD1, IER5L, IFI30, IGFBP5, IL6, IMPA2, KHK, KRT17, LINC01551, LINGO1, LRP3, MAD2L1, MCM2, MCM3, MCM4, MCM5, MCM6, MCM7, MCM8, MELK, MIR22HG, MIR4300HG, MRE11, MSH2, MT1A, MYBL2, MYEOV, MYO19, MYRF, NAT8L, NCAPG2, NCKIPSD, NETO2, NPTX1, NR2C2AP, OLFM2, P2RX5, PCDH12, PCLAF, PDGFB, PIMREG, PLAU, POLA1, POLD1,*

	PRIM1, PRRT4, PSAT1, PSMC3IP, PTPRE, RAC3, RAD51, RAD51AP1, RBBP8, RECQL4, RFC2, RFC3, RFC4, RFC5, RGCC, RGS4, RNASEH2A, RRM1, RRM2, SDC1, SKP2, SLC17A9, SLC19A1, SLC25A10, SLC25A19, SLC47A1, SLC5A6, SPNS2, SRM, TCOF1, TEDC2, TMEM106C, TMEM217, TMEM97, TONSL, TRAF3IP2, TRIP13, TSC22D1, TYMS, UHRF1, UNG, VASN, VPS9D1-AS1, WDR76, YDJC, ZNF367, ZWINT

Supplementary Table 3. Differential expression of KLF6 in ACDMPV endothelial cell (EC) populations.

KLF6 expression in each EC subtype in ACDMPV was compared to its expression in the corresponding cell type in the 3-year-old or preterm neonate control lungs in the snRNA-seq data (PMID: 37463497). Differential expression was performed using Seurat 4 FindMarkers function with Wilcoxon rank sum test. The following criteria were used for significance: p<0.05, FC>=1.5, pct>20%

	snRNA: ACDMPV vs. 3 yr-old control lung										
Symbol in snRNA	Type	ident1	ident2	p_val	p_val_adj	log2FC	pct.1	pct.2	ident1.pct>= 20.nSamp	ident2.pct>= 20.nSamp	DE
KLF6	AEC	AEC_ACDMPV	AEC_Control	9.27E-07	3.39E-02	0.77	49.50%	16.10%	5	1	Up
KLF6	CAP1	CAP1_ACDMPV	CAP1_Control	3.07E-01	1.00E+00	0.09	19.30%	14.80%	1	0	
KLF6	CAP2	CAP2_ACDMPV	CAP2_Control	7.02E-01	1.00E+00	-0.21	24.10%	20.30%	1	2	
KLF6	LEC	LEC_ACDMPV	LEC_Control	1.08E-10	3.96E-06	0.29	71.30%	45.20%	5	3	
KLF6	SVEC	SVEC_ACDMPV	SVEC_Control	3.80E-03	1.00E+00	0.41	54.60%	14.30%	4	1	
KLF6	VEC	VEC_ACDMPV	VEC_Control	1.16E-04	1.00E+00	0.67	47.10%	9.60%	3	0	Up

	snRNA: ACDMPV vs. preterm neonate lung										
Symbol in snRNA	Type	ident1	ident2	p_val	p_val_adj	log2FC	pct.1	pct.2	ident1.pct>= 20.nSamp	ident2.pct>= 20.nSamp	DE
KLF6	AEC	AEC_ACDMPV	AEC_Preterm	2.66E-02	1.00E+00	0.63	49.50%	31.60%	5	2	Up
KLF6	CAP1	CAP1_ACDMPV	CAP1_Preterm	4.80E-01	1.00E+00	0.08	19.30%	23.60%	1	2	
KLF6	CAP2	CAP2_ACDMPV	CAP2_Preterm	9.41E-01	1.00E+00	0.36	24.10%	27.30%	1	2	
KLF6	LEC	LEC_ACDMPV	LEC_Preterm	2.22E-06	8.12E-02	0.47	71.30%	59.50%	5	3	
KLF6	SVEC	SVEC_ACDMPV	SVEC_Preterm	6.58E-01	1.00E+00	0.22	54.60%	44.40%	4	1	
KLF6	VEC	VEC_ACDMPV	VEC_Preterm	1.34E-02	1.00E+00	0.53	47.10%	26.00%	3	2	

.

SUPPLEMENTAL METHODS

Cell Culture

Human Pulmonary Artery Endothelial Cells (HPAECs) were obtained from PromoCell (Germany, Cat. No. C-12241) at passage 3. The cells were used between passages 5-10. Information on the HPAEC donors – including age and gender population doubling time – are provided in Table M1.

Table M1. Donor information for HPAECs used in cell culture experiments.

Lot #	Age	Gender	Doubling Time	Company
431Z031	23	Female	48.3h	PromoCell
433Z034.1	34	Female	55.4h	PromoCell
433Z034.2	34	Female	58.1h	PromoCell
458Z016.12	51	Female	28.5h	PromoCell
458Z016.13	51	Female	28.5h	PromoCell
483Z021.6	49	Male	43.3h	PromoCell
451Z031.14	68	Male	25.4h	PromoCell
18TL155113	42	Male	25.0h	Lonza

Cells were cultured in T75 culture flasks (Sarstedt, Germany, Cat. No. 83.3911.002) that had been pre-coated with bovine plasma fibronectin (10 µg/mL, EMD Millipore Corp, USA, Cat. No. 341631) in an Endothelial Cell Basal Medium 2 (PromoCell, Germany, Cat. No. C-22211). This medium was supplemented with Endothelial Cell Growth Medium2 (EGM2 SupplementPack; PromoCell, Germany, Cat. No. C-39211), 1% penicillin (100 U/mL)-streptomycin (100 µg/mL, Invitrogen, UK, Cat. No. 15140- 122) and 1% MycoZap™ Prophylactic (Lonza, Cat. No. VZA-2032). The cells were then incubated in a humidified incubator at 37 °C with 21% O_2, 5% CO_2, and the endothelial cell growth medium was replaced with a fresh medium every 2-3 days. Only HPAECs displaying endothelium-specific cobblestone morphology at confluence were used for experimentation. The cells were used between passages 5 and 8.

Human Pulmonary Artery Smooth Muscle Cells (HPASMCs) were obtained from Lonza (Walkersville, USA, Cat. No. CC-2581) at passage 3. Donor information is provided in Table M2. Cells were cultured in T75 tissue culture flasks pre-coated with 0.2% porcine gelatin (Sigma, Cat. no. G1890) in smooth muscle cell growth medium 2 (SmGM2, PromoCell, Cat. no. C-22062), supplemented with 5% FBS and growth factor supplement (PromoCell, Cat. no. C-39267) and antibiotics. The cells were used between passages 4-6. For HPAEC and HPASMC co-culture, EGM2 medium was supplemented with 10% foetal bovine serum (FBS). VEGF, heparin, ascorbic acid, and hydrocortisone, known to inhibit HPASMC proliferation, were excluded.

Table M2. Donor information for HPASMCs (Promocell) used in cell culture experiments.

Assigned	Company	Catalogue #	Lot number	Age	Sex
Donor 1	Lonza	CC-2581	0000578443	51	Female
Donor 2	Lonza	CC-2581	0000650387	64	Male
Donor 3	Lonza	CC-2581	0000658401	52	Female

Peripheral blood mononuclear cells (PBMCs) and endothelial colony forming cells (ECFCs).

All ethical regulations relevant to human research participants were followed. Venous blood samples were obtained from healthy volunteers (n=5) and from heritable PAH (HPAH) patients with rare pathogenic BMPR2 variants (n=5)[3] with the informed written consent and approval of the local ethics committee (REC Ref 17/LO/0563) at the Pulmonary Hypertension Clinic, Hammersmith Hospital, Imperial College London. Demographic and clinical characteristics of HPAH patients and healthy volunteers are shown in Table M3.

Peripheral blood mononuclear cells (PBMCs) were isolated using a method described by Ormiston et al. 2015[4] and peripheral blood samples were collected into EDTA coated tubes to avoid coagulation. 50 mL of blood was diluted 1:1 with PBS (Mg^{2+}/Ca^{2+} free), and 22 mL of the diluted blood was carefully applied over 15 mL of Ficoll-Paque Plus gradient solution (GE Healthcare, Amersham, UK, Cat. No. 17-1440-03) in a 50 mL falcon tube and then centrifuged

at 400 g for 30 minutes at room temperature with the brake off. After centrifugation, the buffy coat layer containing the PBMCs was carefully collected into a new 50 mL falcon tube and then diluted 1:1 with PBS and centrifuged at 300 g for 20 minutes with the brake on. Pelleted PBMCs were then either immediately seeded in a 24-wells plate pre-coated with 1% gelatin or resuspended and cryopreserved in a freezing medium containing 10% dimethyl sulfoxide (DMSO; Sigma-Aldrich, UK, Cat. No. D2650) and 90% foetal bovine serum (FBS).

The cells (3-5 $\times 10^7$ cells in 4 mL of EGM2 medium per well) were seeded in 6 well-plates which had been pre-coated with type 1 rat tail collagen (BD Biosciences, Bedford, MA, USA). The culture medium was changed every 24 hours for the first week to remove non-adherent cells and every 48 hours thereafter. Colonies typically appeared between days 7 and 28 and were isolated using an 8 mm or 10 mm in diameter attaching cloning cylinder (Millipore, Watford, UK) with vacuum grease. All the cells from the inside of the cylinder were trypsinised and resuspended in an EGM2 medium and then plated onto 24-well plates which had been precoated with fresh 1% gelatin.

After reaching 70-80% confluence, the colonies were trypsinised and subcultured into 6 well plates and T75 flasks. These late outgrowth endothelial cells (ECFCs) showed stable population doubling times and a typical endothelial cobblestone morphology. The cells were immunostained with endothelial-specific markers VE-cadherin (CDH5), PECAM1 (CD31) and von Willebrand Factor (vWF) to confirm endothelial cell lineage.

ECFCs were cultured in endothelial cell growth medium2 (EGM2, Lonza, Cat. No. CC-3156) containing 20% FBS (HyClone; GE Healthcare, USA, Cat. No. SH30070.03), 1% penicillin (100 U/mL)-streptomycin (100μg/mL, Invitrogen, UK, Cat. No. 15140- 122) and growth factors (EGM-2 bullet kit, Lonza, Cat. No. CC- 4176) in T75 culture flasks pre-coated with 1% gelatin. They were then incubated at 37 °C, 21% O_2, 5% CO_2. ECFCs were used for experiments between passages 3 and 6.

Table M3. Demographic and clinical characteristics of HPAH patients and healthy volunteers. Data represented as median (range). mPAP=mean Pulmonary Arterial Pressure, measured by right heart catheterization; Drug treatment time = time from diagnosis; Mean cardiac index in PAH patients was 1.9 and mean pulmonary vascular resistance (PVR) was 12.9 WU (Woods Units). PDE5=Phosphodiesterase type 5; ET-1R=Endothelin-1 Receptor.

		Control (n=5)	HPAH (n=5)
**Females**		4/5	3/5
**Age (years)**		29 (23- 56)	43 (30-58)
**Time from diagnosis (months)**		-	84 (29-171)
**mPAP (mmHg)**		-	58 (58-70)
**Six-minute walk distance (m)**		-	366 (261-441)
**WHO Functional Class**	**II**	-	3
	**III**	-	2
**PDE5 Inhibitors**		-	5
**ET-1R Antagonists**		-	5
**Prostanoids**		-	2
**Anticoagulants**		-	3
**Statins**		-	1

Adenoviral overexpression KLF2, KLF4 and KLF6 in HPAECs

The overexpression of most common (long) isoforms of KLF2, KLF4 and KLF6 was achieved by adenoviral gene transfer (AdKLF2-GFP (Cat. No. ADV-213187), AdKLF4-FLAG (Cat. No. ADV- 213191), AdKLF6-HA (Cat. No. ADV- 213194); Vector Biolabs (Pennsylvania, USA). Adenoviral control (AdCTRL-GFP) for AdKLF2-GFP was AdGFP (Vector Biolabs, Cat. No. 1060), while AdTet-off [5]was kind gift from Professor Stuart Yuspa (National Cancer Institute, NIH, Bethesda, USA) and was used as adenoviral control (AdCTRL) for AdKLF4 and AdKLF6.

HPAECs were cultured in 6-well cell culture plates or 8-well ibidi μ-slides (Thistle Scientific, UK, Cat. No.IB-80829). At 80% confluency, the cells were infected with AdCTRL, AdGFP, AdKLF2, AdKLF4, AdKLF6 at multiplicity of infection (MOI) of 1:100. 3 hours post infection, the EGM2 media were replaced with a fresh medium, and the cells were then incubated at 37°C, 21% O_2, 5% CO_2 for 24 hours to allow for target gene overexpression before further experimentation.

KLF6 silencing

The knockdown of KLF6 expression was performed using silencer select siRNA targeting KLF6 (siRNA-KLF6, Thermo Fisher Scientific, UK, Cat. No. s3376). Control siRNAs included a scrambled siRNA control (siRNA-CTRL Cat. No. 4390846) and a positive siRNA control targeting GAPDH (siRNA-GAPDH, Cat. No. 4390849).
HPAECs were cultured in 6-well plates in EGM2 media until confluence reached 90%. After 24 hours of incubation, 6 µL of Lipofectamine RNAiMAX reagent (Thermo Fisher Scientific, UK, Cat. No. 13778075) was diluted in a 100 µLOpti-MEM medium (Thermo Fisher Scientific, UK, Cat. No. 31985070). 2 µL(20 pmol) of the appropriate siRNA (10 µM) was diluted in a 100 µL Opti-MEM medium. Following this, 100 µLof Lipofectamine RNAiMAX/OPTIMEM mix was combined with 100 µLof the siRNA/OPTIMEM mix and the mixture was incubated at room temperature for 5 minutes. During this time, the EGM2 medium was removed from the wells, and the cells were then washed with 1 mLof phosphate buffered saline (PBS; Sigma-Aldrich, UK, Cat. No. D8537).

1 mL of OPTIMEM medium and 100 µL of the siRNA /Lipofectamine RNAiMAX/OPTIMEM mix were added to each well. After 6 hours, the cells were washed with 1 mL of PBS, and the EGM2 media (containing 10% FBS) were added to each well. After 24 hours, the medium was removed, the cells were washed with 1 mL PBS, and 1mL of reduced serum EGM2 (containing 0.2% FBS) was added to each well to synchronise the cells for maximal response to stimuli in further experiments. At 48 hours after transfection, the transfected cells were used for experiments. Since the basal KLF6 expression in unstimulated, quiescent HPAECs was insufficient to produce a measurable effect, the effects of KLF6 silencing on cells were studied in cells cultured under flow (4 dynes/cm^2, 24 hours).

HPAEC culture under flow.

HPAECs were cultured in Nunc Slide Flaskettes (Thermo Fisher Scientific, UK, Cat. No. 170920) until 95% confluent. The bottom slides with the cells were then detached from the flask and placed inside the flow chamber in a parallel flow apparatus[6]. The cells were then subjected to a laminar flow at 4 dynes/cm^2, physiological for lung arteries[7], for different periods (30 mins,1h, 4h, 6h and 24h). After the flow exposure, the cells were used for RNA extraction and RT-qPCR to measure mRNA expression levels of KLF2, KLF4 and KLF6.

Real-time quantitative PCR (qPCR), RNA sequencing (RNA-seq) and spatial transcriptomics

RNA extraction from lung tissues. Frozen lung tissue samples from MCT rats were cut into small pieces and placed in a BioMasher tube (Takara Bio Europe, France, Cat. No. 9791A) containing 100 µL of TRIzol Reagent (Life Technologies, UK, Cat. No. 15596026). After grinding the samples 10-15 times, 600 µL of TRIzol Reagent was added and the homogenized solution was transferred into 1.5 mL Eppendorf tubes and vortexed for ~30 seconds. 140 µLRNase-free chloroform (Thermo Fisher Scientific, UK, Cat. No. 327155000) was added and the sample was incubated at room temperature for 2-3 minutes and then placed on ice. The mixture was centrifuged at 12,000 g for 15 minutes at 4°C. The upper aqueous phase was transferred into a new Eppendorf tube. 4 µL of glycogen (5 mg/mL; Thermo Fisher Scientific, UK, Cat. No. AM9510) and 450 µL of Isopropanol (Thermo Fisher Scientific, UK, Cat. No. 327272500) were then added and inverted to mix for 10 seconds. The mixture was placed in a fridge at 4°C for 10 minutes to enhance RNA precipitation, and was then centrifuged at 12,000 g for 10 minutes at 4°C. The supernatant was aspirated, and the pellet was air dried for about 10 minutes. The RNA pellet was then resuspended in 20 µL RNase-free water. The total RNA was quantified using a NanoDrop^{TM} ND-2000 spectrophotometer (Thermo Scientific, UK).

RNA extraction from cultured cells. HPAECs were washed with pre-warmed PBS, then trypsin solution (Gibco, UK, Cat. No. 25200072) was added, followed by incubation at 37°C, 21% O_2, 5% CO_2 for ~5 minutes. Dulbecco's Modified Eagle's Medium (DMEM; Sigma-Aldrich, UK, Cat. No. D6429), supplemented with 10% foetal bovine serum (FBS; Thermo Fisher Scientific, UK, Cat. No. 10270098), was then added to neutralize the enzymatic reaction of the trypsin. The cell suspension was transferred to a 1.5 mLRNase-free microcentrifuge tube and centrifuged at 500 g for 5 minutes at room temperature. The supernatant was discarded, and the cell pellet was washed once with PBS and pelleted again. The cell pellet was either stored at -80 °C to be used later, or was used immediately for RNA extraction. Before starting the RNA extraction procedure, the bench surfaces, pipettes and all the equipment was sprayed with RNaseZap™ RNase Decontamination Solution (Invitrogen, USA, Cat. No. AM9780).

Total RNA was extracted using Monarch Total RNA Miniprep Kit (New England Biolabs UK, Cat. No. T2010S) according to the manufacturer's instructions. All centrifugation steps were performed at (8,000-16,000 x g). Briefly, 300 µL of RNA Lysis Buffer containing 1% beta-mercaptoethanol (β-ME) were added to the cell pellet and the sample was vortexed vigorously

for 30 seconds to lyse the cells. The mixture was then transferred to a gDNA removal column and centrifuged for 30 seconds to remove the genomic DNA. After centrifuging, the gDNA removal column was discarded and the flow-through in the collection tube which contained the RNA was saved. 300 µL of ethanol (≥ 95%) were added to 300 µL of the flow-through and mixed thoroughly by pipetting up and down. The mixture was then transferred to an RNA Purification Column and centrifuged for 30 seconds. After discarding the flow-through, 500 µL of RNA Wash Buffer were added to the RNA Purification Column and centrifuged for 30 seconds. The flow-through was then discarded, and 500 µL of RNA Priming Buffer were added and centrifuged again for 30 seconds. For the second wash, another 500 µL of RNA Wash Buffer were added and centrifuged for 1 minute. After discarding the flow-through, the RNA Purification Column was placed in a RNase-free microcentrifuge tube and the RNA was eluted in 50 µL nuclease-free water and centrifuged at maximum speed of 16,000 x g for 1 minute. The elution step was repeated twice by pipetting the eluted RNA back into the same RNA purification column and centrifuging it for 1 minute to maximize total RNA yield per sample. RNA concentration was determined using a NanoDrop™ ND-2000 spectrophotometer (Thermo Fisher Scientific, UK).

The integrity of all RNA samples used in this study, including those used for RT-qPCR analyses and RNA-seq, was assessed in an Agilent TapeStation 2200 (Agilent Technologies, UK) using High Sensitivity RNA ScreenTape (Agilent, cat no 5067-5579) and High Sensitivity RNA ScreenTape Ladder (Agilent, cat no 5067-5581). The RNA Integrity Number equivalent (RINe), a user-independent algorithm that automatically assesses RNA degradation, was calculated automatically using the Agilent TapeStation. All samples used in this study had RIN values of 8 or above, which indicates good sample integrity and low levels of degradation. To ensure the quality of the samples was kept high after QC, we only worked on surfaces cleaned with RNaseZap™ (Fisher Scientific, cat. no 10708345). Samples were manipulated wearing gloves and RNase-free filter tips. All reactions were carried out in RNase-free tubes (Life Technologies, cat. no AM12450). To further ensure maintenance of RNA sample integrity, samples were stored at -80˚C and thawed on ice prior to reverse transcription and NGS library preparation.

Reverse Transcription (RT). 100 ng of the total RNA was reverse-transcribed into complementary DNA (cDNA) using a LunaScript RT SuperMix Kit (New England Biolabs, UK, Cat. No. E3010L) according to the manufacturer's instructions. 20 µL reaction volume

were prepared in 96-well PCR plates (Thermo Fisher scientific, UK, Cat. No. 4306737), as shown in Table M4. A negative No-RT control was added to each plate to verify the absence of DNA contamination in the RNA samples. Before the reverse transcription, the PCR plates were sealed, centrifuged and placed in a SimpliAmp™ Thermal Cycler (Applied Biosystems, USA). Thermal conditions are shown in Table M5. cDNA samples were then diluted to 1 ng/µL using nuclease-free water and stored at -20°C.

Table M4. Components of reverse transcription reaction mix. Modified from New England Biolabs (https://uk.neb.com).

Component	20 µL Reaction	Final Concentration
LunaScript RT SuperMix (5x)	4 µL	1X
RNA sample	variable	100ng
Nuclease-free Water	up to 20 µL	-

Table M5. Thermal conditions for reverse transcription. Modified from New England Biolabs (https://uk.neb.com).

**Cycle Step**	**Temperature**	**Time**	**Cycles**
Primer Annealing	25°C	2 minutes	1
cDNA Synthesis	55°C	10 minutes	1
Heat Inactivation	95°C	1 minute	1
Hold	4°C	∞	1

Real-time quantitative PCR (qPCR).

A qPCR master mix solution was prepared for each gene of interest as described in Table M6 below.

Table M6. Components of qPCR master mix. Modified from New England Biolabs (https://uk.neb.com).

Component	10 µL Reaction	Final Concentration
Luna Universal qPCR Master Mix	5 µL	1X
10 µM forward primer	0.5µ L	0.25 µM
10 µM reverse primer	0.5 µL	0.25 µM
Nuclease-free water	3 µL	-
cDNA products	1 µL	1ng

9 µL of qPCR master mix solution were added in a 384-well PCR plate (StarLab, UK, Cat. No. E1042-3840). After preparing the qPCR master mix solution, 1 ng/µL of the cDNA sample was first added in a 384-well PCR plate (StarLab, UK, Cat. No. E1042-3840), and 9 µL of qPCR master mix solution was then added to each well of the plate. A negative control was included for each gene of interest in which the cDNA was replaced by Nuclease-free water. The qPCR was carried out using a QuantStudio™ 6 Flex Real-Time PCR System (Applied Biosystems, USA). Amplification steps for qPCR reactions are described in Table M7.

Table M7. Cycling conditions for qPCR.

Cycle Stage	Temperature	Time	Cycles
Hold Stage	50°C	2 minutes	1
	95°C	5 minutes	
PCR Stage	95°C	15 seconds	40
	62°C	30 seconds	
Melt Curve Stage	95°C	15 seconds	1
	60°C	1 minute	
	95°C	15 seconds	

Gene-specific primers for qRT-PCR were designed using FASTA sequences (PubMed, NCBI) and PrimerBlast (NCBI, USA), using default parameters. At least one primer in each pair spanned the exon–exon junction. All primer sequences are listed in Table M8.

The relative expression level was calculated using the $2^{-\Delta\Delta CT}$ method with a target gene that had been normalized to the expression of appropriate housekeeping gene, which was actin beta (*ACTB*) for static experiments or beta-2 microglobulin (*B2M*) for flow experiments.

Table M8. Primer sequences used for qPCR.

Gene	Forward Primer (5'-3')	Reverse Primer (5'-3')
**Human**		
KLF2	GCAAGACCTACACCAAGAGTTCG	CATGTGCCGTTTCATGTGC
KLF4	TGGACCCCCTCTCAGCAATG	CTCTTGGTAATGGAGCGGCG
KLF6	GGGTGTGGCTCTTTGCTTTA	AGCGTTAGTCACTGCTCATTTC
BMPR2	CTGCGGCTGCTTCGCAGAAT	TGGTGTTGTGTCAGGAGGTGG
SOX17	GGACCGCACGGAATTTGAAC	GGACACCACCGAGGAAATGG
ERG	GGAGTGGGCGGTGAAAGA	AAGGATGTCGGCGTTGTAGC
MKI67	TGCTCCCCACCTCAGAGAGTT	GACCTTCCTGACCTGTTTGCAG
PCNA	GTGAACCTCACCAGTATGTCCAA	ACTAGCGCCAAGGTATCCGC
BRD4	GATTGCCGGCTCCTCCAAGAT	ATGGTGCTTCTTCTGCTCCCTC
PECAM1	TGGAAGGAGTGCCCAGTCCCA	CGGAAGGATAAAACGCGGTCCTG
CDH5	CCTCCGATACATGAGCCCTCC	TCGGAAGAACTGGCCCTTGT
KDR	CACCAGAAATGTACCAGACCATGC	AGTCCAGAATCCTCTTCCATGCTC
HES1	AGAAAGATAGCTCGCGGCATT	CGGAGGTGCTTCACTGTCAT
FLT1	GTTTCCAGACCCGGCTCTCT	TGGTTACAGGGGTGCCAGAA
TIE2	CCCTGAATGCCCCAAACGTG	ACGGACCAGTTGCACACAGA
SLC2A1	GGGCCAAGAGTGTGCTAAAGA	GGTGACCTTCTTCTCCCGCA
ACTB1	GCACCACACCTTCTACAATGA	GTCATCTTCTCGCGGTTGGC
B2M	CCAGCGTACTCCAAAGATTCAGG	TCAATGTCGGATGGATGAAACCC
**Rat**		
ACTB	ACCCCAGCCATGTACGTAGC	TGCCTGGGTACATGGTGGTG
KLF2	ACTTGCAGCTACACCAACTG	CTGTGACCCGTGTGCTTG
KLF4	TGAACTGACCAGGCACTACC	GCCTCTTCATGTGTAAGGCA
KLF6	CGGACGCACACAGGAGAAAA	CGGTGTGCTTTCGGAAGTG
**Mouse**		

B2M	GCCTGTATGCTATCCAGAAAAC<br>C	TCAATGTGAGGCGGGTGGAA
KLF2	GAGCCTATCTTGCCGTCCTTT	CACGTTGTTTAGGTCCTCATCC
KLF4	CCAGAGGAGCCCAAGCCAAAG	CGGTAGTGCCTGGTCAGTTCAT
KLF6	ACTGTCTTTTCCAACCCGAC	AAGATAGCGTTCCAACTCCAG

RNA Sequencing

RNA sequencing (RNAseq) (75bp paired-end reads) was performed in an Illumina NextSeq 2000 sequencer (Illumina, USA) at the Imperial BRC Genomics Facility (Imperial College London) to assess the whole transcriptomic effects of KLF2, KLF4, and KLF6 overexpression by adenoviral gene transfer (AdKLF2, AdKLF4 and AdKLF6 conditions, respectively).

The quality of the paired end reads within the fastq files were assessed using FastQC (https://www.bioinformatics.babraham.ac.uk/projects/fastqc/) and MultiQC. To determine transcript abundance values, Salmon (v1.5.2) was used to map sequencing reads in the fastq files to the human genome (GENCODE v44) (https://www.gencodegenes.org/human/) and to obtain gene counts. To identify differentially expressed genes (DEGs), the gene expressions of the AdKLF2, AdKLF4 and AdKLF6 conditions (n=4 biological replicates/condition) were compared with the corresponding negative control conditions using the R package DESeq2 (v1.38.3)[8], with default settings. Enhanced volcano (v1.16.0) (https://github.com/kevinblighe/EnhancedVolcano 2020) and heatmaps were generated using the R package pheatmap (v1.0.12). Gene annotation was performed using biomaRt (2.46.3). The list of PAH genes was derived from [9].

Gene ontology (GO) and Kyoto Encyclopaedia of Genes and Genomes (KEGG) enrichment analysis

GO and KEGG pathway enrichment analyses were carried out separately on the sets of up- and down-regulated genes from each comparison using the Metascape (https://metascape.org) online tool[10], the WEB-based GEne SeT AnaLysis Toolkit (WebGestalt) (https://www.webgestalt.org)[11] and the clusterProfiler package (v3.14.3) in R. The DisGeNET enrichment analysis and network of enriched terms of up- and down-regulated genes was performed using Metascape. Genes that passed the thresholds of FDR <0.01, absolute value of $\log_2$ (fold change)>2 were used for GO and KEGG pathway enrichment analyses, unless indicated otherwise.

Comparative analysis with published RNA-seq datasets and disease-gene databases. To investigate whether differentially expressed genes from KLF6-overexpressing HPAECs were enriched in PAH-related genes, a comparative analysis was carried out using RNA-seq datasets from HPAECs and ECFCs from the "double hit" microfluidic model of PAH[3] and from PAECs that had been isolated from IPAH patients[12] or with known PAH and IPAH-related genes from DisGeNET databases (https://www.semanticscholar.org/paper/GeneOverlap%3A-An-R-package-to-test-and-visualize-Shen/117e12840af966176bbc348db6edf034b0ea479c). Overlapping genes were visualized as UpSet plots using the ComplexUpset package (v1.3.3) in R. The significance of the overlaps between gene sets in comparison to the genomic background was calculated by one-tailed Fisher's exact test using the GeneOverlap package (v.1.22.0) in R (https://www.semanticscholar.org/paper/GeneOverlap%3A-An-R-package-to-test-and-visualize-Shen/117e12840af966176bbc348db6edf034b0ea479c)

Spatial transcriptomics

To assess the transcriptomic changes in PAH lungs, spatial transcriptomics of lung tissues from healthy controls and PAH patients were carried out using NanoString GeoMx® to enable high-plex spatial profiling of RNA or protein within specific areas of interest in the tissue [13]

Formalin fixed and paraffin embedded (FFPE) lung tissue sections (3-4µm) from PAH patients (n = 6) and age- and sex-matched healthy volunteers (n = 5) were obtained from the Royal Papworth Hospital NHS Foundation Trust Tissue Bank (Cambridge, UK) with informed written consent and ethical approval by (ICHTB HTA licence: 12275; REC Wales approval: 22/WA/0214). Donor information for FFPE lung tissue samples is shown in Table M9.

Table M9. List of human lung tissue donors used in this study. The first control (a 49-year-old female) was excluded due to the abnormal appearance of the lung tissue.

Pair	Category	Age	Sex
1	Control	49	Female
	PAH	32	Female
2	Control	53	Female

	PAH	51	Female
3	Control	60	Female
	PAH	57	Female
4	Control	21	Male
	PAH	27	Male
5	Control	58	Male
	PAH	37	Male
6	Control	64	Male
	PAH	61	Male

.

The lung tissue slides were first hybridized overnight at 37°C with GeoMx Human Whole Transcriptome Atlas Human RNA probe mix (GeoMx Hu WTA, NanoString Technologies, USA). After incubation with the RNA probe mix, stringent washes were performed using a mixture of an equal volume of 100% (v/v) formamide and 4X saline-sodium citrate (SSC) to remove off-target probes and the slides were then blocked with 200 µL Buffer W (NanoString Technologies, USA) and incubated for 30 minutes at room temperature in a humidity chamber. The slides were stained with SYTO13 (nuclear marker), vWF (von Willebrand Factor; endothelial cell marker), and ACTA2 (Alpha-smooth muscle actin or α-SMA; smooth muscle cell marker) to allow the identification of blood vessels during the ROI selection. The slides were then washed with 2x SSC for 5 minutes twice and immediately loaded into the GeoMx Digital Spatial Profiler (DSP) instrument for scanning (x20 magnification) and ROI selection. For each lung tissue section, a total of 30 regions of interest (ROIs) were selected with the "circle" or "freehand" selection tool available on the GeoMx DSP software to profile blood vessels within the lungs, specifically focusing on plexiform lesions observed in PAH patients. After ROI selection on the GeoMx DSP instrument, the DSP barcodes in ROIs were ultraviolet (UV)-cleaved and collected into a 96-well DSP collection plate before being sequenced (paired-end reads) on an Illumina sequencer (Illumina, USA) at the Imperial BRC Genomics Facility (Imperial College London).

Spatial transcriptomics data analysis and visualization

The raw counts were processed using NanoString's GeoMx NGS pipeline software V2.2 (Nanostring Technologies, USA), where they were converted into digital count conversion (DCC) files. The GeomxTools package (V3.5.0) was used for quality control (QC), and the downstream analysis of the DCC files was performed using R

(https://bioconductor.org/packages/GeomxTools/). Differential gene expression analysis was performed using a linear mixed model (LMM) as recommended in the GeomxTools manual. The adjusted p values were calculated using the Benjamini-Hochberg multiple test correction, and differentially expressed genes in all analyses were defined as FDR <0.05 and absolute value of $\log_2$ (fold change) > 0.25. For spatial deconvolution, the SpatialDecon R package (https://www.nature.com/articles/s41467-022-28020-5) was used to identify the cell type composition of each ROI using the IPF Lung Cell Atlas (https://www.science.org/doi/10.1126/sciadv.aba1983) as the cell profile matrix, which is composed of 36 cell types.

Gene ontology and KEGG pathway analyses were performed using the WEB-based Gene SeT AnaLysis Toolkit (WebGestalt) (https://www.webgestalt.org)

[11] The ComplexUpset R package (v1.3.3) was used to visualize the number of overlapping genes between different DE gene datasets (https://zenodo.org/records/7314197).

The GeneOverlap R package (v.1.22.0) was used to test the degree and significance of overlap between two gene lists in comparison with a genomic background using Fisher's exact test (https://www.semanticscholar.org/paper/GeneOverlap%3A-An-R-package-to-test-and-visualize-Shen/117e12840af966176bbc348db6edf034b0ea479c)

Analysis of endothelial *KLF6* expression in single nucleus RNA sequencing of alveolar capillary dysplasia with misalignment of pulmonary veins (ACDMPV) and control lungs

Integrated single nucleus RNA-seq (snRNA-seq) of ACDMPV and control lungs were obtained from Guo et al. [14], which contains snRNA-seq of five ACDMPV lungs (2 weeks to 3.5 years), three 3-year-old control lungs, and three preterm neonate lungs (1-4 days old, 29-31 weeks of gestational age). Detail donor information can be found in Guo et al[14]. We subset the integrated data to the identified endothelial cells (ECs). Gene expression was SoupX-corrected and normalized by Seurat NormalizeData function. Differential expression test was performed in each EC cell type for *KLF6* between ACDMPV vs. 3-yr control lung cells and between ACDMPV vs. preterm neonate lung cells.

Tests were performed using the Seurat 4 FindMarkers function using Wilcoxon Rank Sum test. Genes with the following criteria were considered up in an EC subtype in ACDMPV: p_val<0.05, log2FC>=log2(1.5), pct.1>=0.2, "ident1.pct>=20.nSample" >=2 (i.e., pct>=20% in at least 2 ACDMPV samples) in the cell type. Genes with the following criteria were considered down in an EC subtype in ACDMPV: p val<0.05, log2FC <= -log2(1.5), pct.2>=0.2, "ident2.pct>=20.nSample" >=2 (i.e., pct>=20% in at least 2 Control or 2 Preterm

samples) in the cell type.

Immunocytochemistry (ICC)

Cells were cultured in ibidi μ-slide 8 well chamber slides (Thistle Scientific, UK, Cat. No. IB-80806) or on plastic coverslips (13mm diameter, Nunc Thermanox, USA, Cat. No. 174950). They were then washed once with pre-warmed PBS and fixed with 4% paraformaldehyde (w/v) (Sigma-Aldrich, UK, Cat. No. P6148) in PBS for 15 minutes at room temperature. After fixation, the cells were washed twice with PBS and permeabilized using 0.1% TritonX-100 (v/v) (Sigma-Aldrich, UK, Cat. No. 9002-93-1) in PBS. They were then gently washed twice with PBS.

For immunostaining of nuclear KLF6, PBS was removed, and the cells were incubated in 100% methanol for 5 minutes at -20°C, then air dried for 1 minute. The non-specific binding sites were then blocked by incubating cells with 2% bovine serum albumin (w/v) (BSA; Sigma-Aldrich, UK, Cat. No. A2058) in PBS for 15 minutes at room temperature, followed by incubation with primary antibody overnight at 4 °C, then secondary antibody for 2 hours at room temperature. Primary and secondary antibodies were both diluted in 0.1% BSA (v/v) in PBS. After each incubation time with the antibody, the cells were washed three times for 5 minutes in PBS. All primary and secondary antibodies used are listed in Table M10. Samples were then mounted in Vectashield Antifade Mounting medium containing nuclear stain DAPI (Vector Laboratories, Cat. No. H-1200).

Immunofluorescence images were observed using a STELLARIS 8 inverted confocal microscope (Leica Microsystems, Mannheim, Germany) at 10x or 20x objectives.

Immunohistochemistry (IHC)

Immunohistochemical staining was carried out as described[15], with some modifications. Briefly, formalin fixed and paraffin embedded lung tissue sections of healthy control (n = 6) and PAH patients (n = 6) (listed in Table M10) (Royal Papworth Hospital NHS Foundation Trust Tissue Bank, Cambridge, UK), sections of human placenta and lung tissue from a patient with pulmonary tuberculosis (Imperial College Healthcare Tissue Bank) were dewaxed in Histo-Clear (National Diagnostics, USA, Cat. No. HS-202) twice for 5 minutes, before being rehydrated in graded ethanol solutions (100%, 70% and 50%) (v/v) in distilled water (VWR, UK, Cat. No. 20821.330) for 5 minutes per wash and then washed twice

in distilled water for 5 minutes per wash. The sections were then subjected to antigen retrieval by boiling them at 95°C for 10 minutes (2 x 5 minutes on a hot plate to avoid overboiling) in a solution containing 40 mL of Low pH IHC Antigen Retrieval Solution (v/v) (Life Technologies, UK, Cat. No.00-4955-58) and 360 mL of distilled water. The sections were then washed in PBS three times for 5 minutes per wash. After air-drying, the tissue sections were encircled with a Hydrophobic Barrier PAP Pen (Thermo Scientific, UK, Cat. No. R3777). The tissue sections were then blocked with 5% bovine serum albumin (BSA) (v/v) in PBS with 4 drops of 2.5% Normal Horse Serum (Vector Laboratories, USA, Cat. No. S-2012) added and incubated for 1 hour at room temperature in a humidified chamber. After the removal of the blocking solution, sections were incubated overnight with primary antibodies diluted in 5% BSA in PBS in a humidified chamber at 4 °C before being washed with PBS three times for 5 minutes per wash. The sections were then incubated with secondary antibodies diluted in 5% BSA in PBS for 2 hours at room temperature in a humidified chamber before being washed with PBS three times for 5 minutes per wash. The primary and secondary antibodies used are listed in Table M12. The slides were then mounted in Vectashield Antifade Mounting medium with nuclear stain DAPI. Immunofluorescence images of lung tissues were acquired using a STELLARIS 8 inverted confocal microscope (Leica Microsystems, Mannheim, Germany).

Table M10. List of primary and secondary antibodies used for **immunofluorescence staining.** vWF, von Willebrand factor; α-SMA and α-Smooth Muscle Actin. In addition to primary and secondary antibodies, phalloidin-TRITC (Bio-Techne, UK, Cat. No. 5783) was used as an affinity-stain for filamentous actin (F-actin) in endothelial cells.

Antibody Name	Species	Type	Dilution	Cat No.	Company
KLF2 (Anti-Human)	Mouse	Monoclonal IgG	1:200	MAB5466	R&D Systems
KLF4/GKLF (Anti-Human)	Mouse	Monoclonal IgG	1:100	sc-393462	Santa Cruz Biotechnology
KLF6 (Anti-Human)	Mouse	Monoclonal IgG	1:100	sc-365633	Santa Cruz Biotechnology
KLF6 (Anti-Human)	Rabbit	Polyclonal IgG	1:100	PA5-84819	Invitrogen
ERG (Anti-Human)	Mouse	Monoclonal IgG	1:200	sc-376293	Santa Cruz Biotechnology

VE-Cadherin/CD144 (Alexa Fluor 488) (Anti-Human)	Mouse	Monoclonal IgG	1:200	53-1449-42	Invitrogen
α-SMA (Cy3-Conjugated) (Anti-Human)	Mouse	Monoclonal IgG	1:300	C6198	Sigma-Aldrich
CD31/PECAM1 (Anti-Human)	Mouse	Monoclonal IgG	1:500	M0823	Dako
HA-Tag (Anti-Human)	Rabbit	Monoclonal IgG	1:300	C29F4	Cell Signaling Technology
FLAG (Anti-Human)	Rabbit	Polyclonal IgG	1:300	F7425	Sigma-Aldrich
vWF (Anti-Human)	Rabbit	Polyclonal IgG	1:200	A0082	Dako
Cy5-Conjugated (anti-Mouse)	Goat	Polyclonal IgG	1:200	16855-AAT	AAT Bioquest
Alexa Fluor Plus 594 (Anti-Mouse)	Goat	Polyclonal IgG	1:200	A32742	Invitrogen

Semi-quantitative evaluation of KLF6 protein expression in lung tissues.

The intensity of fluorescence was measured with FIJI (Image J) in three 800 μm^2 1024x1024 pixels confocal images/section. The images were acquired under 20x objective with constant setting of laser power (20%), detector gain (727), pinhole size (416µm, 4.9 air), zoom (1x).

NF-κB Luciferase Reporter Assay

HPAECs that had been cultured in 96-well plates or in 6 channel µ-Slides (Thistle Scientific, UK, Cat. No. IB-80806) were left untreated or were infected with adenoviral NF-κB luciferase reporter[15] (AdNFkB-luc, Vector Biolabs, Cat. No.1740), AdCTRL and AdKLF6 or were transfected with siRNA-KLF6 and scrambled-siRNA control, as required.

Since the basal KLF6 expression in unstimulated, quiescent HPAECs was insufficient to produce a measurable effect, the effects of KLF6 silencing were studied in cells cultured under flow.

3 hours after adenoviral infection or 48 hours after siRNA exposure, the culture media were replaced with a fresh EGM2 medium with or without 10 ng/mL of TNF-α (R&D Systems, USA, Cat. No. 210-TA-020). The cells were then incubated under either normoxic or hypoxic conditions for 24 hours. A luciferase reporter assay (Promega, USA, Cat. No. E1500) was then performed according to the manufacturer's instructions. Briefly, 20 µLof cell lysates were combined with 100 µL of luciferase assay reagent in 96-microwell white opaque polystyrene plates (Thermo Scientific, UK, Cat. No. 136101). The intensity of luminescence, proportional to the level of NF-κB–driven luciferase activity, was measured in GloMax® luminometer (Promega, UK).

Transwell permeability assay.

Endothelial permeability assay was carried out using a 6.5 mm transwell plates with sterile 0.4 µm pore size polycarbonate membrane inserts (Corning, USA, Cat. No. 3413).

Cells were seeded at a density 2 $x10^4$ cells per insert with 200 µLof cell culture medium in the apical compartments of transwell inserts that had been pre-coated with bovine plasma fibronectin (10 µg/mL, EMD Millipore Corp, USA, Cat. No. 341631). 500 µLof the medium was then added to the basal compartments of the transwell. Once the cells formed a confluent monolayer, they were infected with either AdCTRL or AdKLF6. After 2 hours of adenoviral infection, the media were changed and the cells were either left untreated or were treated with 10 ng/mL of TNF-α (R&D Systems, USA, Cat. No. 210-TA-020). The cells were then placed in normoxia (21% O_2) or hypoxia (2% O_2) at 37°C and 5% CO_2 for 24 hours. After this period, the cells were incubated in fresh medium containing 1 mg/mL of 40 kDa FITC-Dextran (Sigma-Aldrich, Dorset, UK, Cat. No. FD40S) for 1 hour. After an hour of incubation, 500 µLof the medium was collected from the basal compartment, and the fluorescence intensity of FITC- dextran was measured at excitation/emission 490/525 nm using a GloMax® luminometer (Promega, UK).

To test thrombin-induced cell permeability, cells were infected with either AdCTRL or AdKLF6. After 24 hours, they were left untreated or stimulated with 1 U/mL of thrombin (Sigma-Aldrich, Dorset, UK, Cat. No. T7513) in a serum-free medium containing 1 mg/mL FITC-Dextran for 1 hour, then the rest of experiment was carried out as described above.

Angiogenesis assays.

Angiogenesis was assessed in a tube formation assay *in vitro* and in an *ex vivo* pulmonary arterial explants sprouting assay.
For the tube formation assay, HPAECs were infected with either AdCTRL or AdKLF6. 24h after infection, the cells were seeded into a 96-well plate pre-coated with growth factor-reduced Matrigel (Scientific Laboratory Supplies, UK, Cat. No. 354230). The cells were then incubated for 20 hours under normoxic conditions. After 20 hours of incubation, endothelial tube formation was imaged using a phase-contrast microscope. Images were then analysed using ImageJ software (Fiji, version 2.14.0) with an Angiogenesis Analyzer plugin to quantify the number of nodes and meshes as well as the total tube length.

For the arterial explants sprouting assay, surgical specimens of human pulmonary arteries were cleaned of blood and attached connective tissues in PBS. Donor characteristics – including age, gender, height, weight and diagnosis – are summarized in Table M11. The arteries were cut longitudinally with a scalpel, opened and pinned down on the dissection dish with the endothelium facing up. 50-100 μL of EGM2 containing AdCTRL or AdKLF6 was added on top of the endothelial layer, and the explants were then incubated for 3 hours in a humidified incubator. Afterwards, the arterial tissue was thoroughly washed in PBS and cut into roughly 1 mm^2 fragments which were then embedded in Matrigel and incubated for 3 weeks in a humidified incubator under normoxic conditions. AdKLF6 was added to the explants again at 2 weeks to maintain the efficiency of gene transduction. Endothelial sprouts were fluorescently labelled with Vybrant™ CFDA SE Cell Tracer (Invitrogen, USA, Cat. No. V12883) before being imaged under a fluorescent microscope. The total area of sprouting was measured using ImageJ software (Fiji, version 2.14.0).

Table M11. Pulmonary artery donor characteristics. ADC: adenocarcinoma, AVM: arteriovenous malformation.

ID	Age	Gender	Height (cm)	Weight (kg)	Diagnosis
CX22000050	56	M	170	79.8	Pulmonary AVM
CX22000077	33	F	160	88.9	Pulmonary AVM
CX22000115	70	F	158	53	Non-mucinous lung ADC
CX22000116	21	F	157	45.7	Pulmonary AVM
CX22000221	72	M	181	76	Non-mucinous lung ADC

EdU Cell Proliferation Assay

Cell proliferation assay was performed using an EdU Cell Proliferation Assay Kit (EdU-594, EMD Millipore Corp, USA, Cat. No. 17-10527), according to the manufacturer's instructions. EdU solution was diluted 1:1000 in cell culture medium. 24 hours later cells were fixed with 3.7% formaldehyde in PBS for 15 minutes and permeabilized using 0.5% Triton X-100 in PBS for 20 minutes. The cells were then treated with an EdU reaction cocktail which was prepared according to the manufacturer's instructions and were then incubated for 30 minutes.

Cells were mounted with Vectashield Antifade Mounting medium containing DAPI (Vector Laboratories, H-1200). Images of EdU positive cells were acquired under a fluorescent Zeiss Axio Observer widefield microscope (Carl Zeiss AG, Germany) at a 10x magnification and analysed with ImageJ software (Fiji, version 2.14.0). The results were quantified as a ratio of the number of EdU positive cells against the total number of cells.

Proliferation of PASMCs in co-culture with HPAECs.

Control (AdCTRL) and AdKLF6-overexpressing HPAECs were grown to confluence in 6.5 mm Transwell inserts with 1.0 μm pore size transparent PET membrane (Corning, Cat. No 353104) in EGM2 culture media containing serum and growth factors. HPASMCs were seeded at the bottom of 24-well plates (50 x10^3 cells/well) or on the other side of empty Transwell insterts or inserts containing endothelial cells (20 x 10^3 cells/insert) in EGM2 medium containing 5% FBS and no growth factors. Following 2-hour incubation in a humidified culture incubator, unattached PASMCs were washed away with PBS and Transwell inserts containing HPAECs or co-cultures of HPAECs and HPASMCs were inserted into the wells of 24 well plate. Culture media were replaced with EGM2 medium containing 0.2% FBS, antibiotics and EdU (for further detail please see EdU Cell Proliferation Assay), with no growth factors. PASMCs cultured in full PASMC medium served as a positive control.

Wound healing assay.

Endothelial cell migration was assessed in a wound healing assay using ibidi Culture-Insert 2 Well in μ-Dish 35 mm (ibidi, Germany, Cat. No. 81176) or in a scratch assay under flow. Cells were seeded at a density of 3×10^4 cells per well. After overnight incubation, the cells were infected with either AdCTRL or AdKLF6. Next day, cells were serum- and growth factor starved (0.2% FBS, with no growth factors) for 6 hours. Culture inserts were removed

after starvation, and the cells were washed with PBS to remove non-adherent cells. They were then either left in a starvation medium (0.2% FBS, with no growth factors) or were stimulated by the addition of full EGM2 containing serum and growth factors (EGM2, 2 mL) to the dish. Images of the cells were captured at 0 hour and 20 hours using a Rebel phase contrast microscope (ECHO, USA) with a 10x objective.

In the scratch assay, HPAECs were seeded in Nunc Lab-Tek Flaskettes (Thermo Fisher Scientific, UK, Cat. No. 170920) at a density of 2×10^5 cells per Flaskette. After an overnight incubation, the cells were transfected with KLF6 siRNA or negative control siRNA. After 48 hours, the confluent endothelial cell monolayers were serum- and growth factor starved (0.2% FBS, with no growth factors) for 6 hours. The bottom slides containing the cells were then detached and placed into petri dishes (150 x15 mm standard style, VWR International Ltd, UK, Cat. No. 391-2003). A straight "wound" was created by scratching endothelial monolayers with a sterile 200 µL pipette tip in parallel to the direction of flow, as in[16]. The slide was then washed in PBS and placed inside the flow chamber of a parallel flow apparatus, where the cells were exposed to laminar flow (4 dynes/cm^2) for 20 hours. Images of the wound were taken at 0 hour and 20 hours using a phase contrast microscope (ECHO Rebel, USA) with a 10x objective.

The wound area was measured using a custom-made Wound Healing Tool ImageJ macro (written by Stephen Rothery, Facility for Imaging by Light Microscopy (FILM), Imperial College London)

.

Apoptosis Caspase-Glo 3/7 Assay

Apoptosis was assessed by measuring caspase 3/7 activity using a Caspase-Glo 3/7 Assay Kit (Promega, Southampton, UK, Cat. No. G8091), according to the manufacturer's instructions. Apoptosis was measured in control and KLF6-overexpressing HPAECs following serum- and growth factor- starvation or treatment with staurosporine (STS; 0.1µM, 24h) [17] (Sigma, Cat. No. S5921). Briefly, following the cell treatments, Caspase-Glo 3/7 reagent was prepared by adding a Caspase-Glo 3/7 substrate to buffer and allowing them to equilibrate to room temperature. An equal volume of reagent (100 µL) was added to each well of the 96-well plate containing 100 µL of blank (medium only) and treated cells in a culture medium.

The plate was then placed on a plate shaker at 300-500 rpm for 30 seconds and incubated at room temperature for 1 hour. Luminescence was measured using a GloMax® luminometer (Promega, UK), according to the manufacturer's instructions.

TUNEL Apoptosis Assay

TUNEL Assay was performed using a Click-iT Plus for In Situ Apoptosis Detection Kit with Alexa Fluor 647 dye (Invitrogen, Cat. No. C10619), according to the manufacturer's instructions. This assay was used to evaluate apoptosis in HPAECs 48 hours following KLF6 silencing. Control siRNA- or KLF6 siRNA-treated cells were subjected to flow of 4 dynes/cm^2 with, or without STS (0.1μM)[17]. TUNEL positive cells were identified using a fluorescent Zeiss Axio Observer widefield microscope (Carl Zeiss AG, Germany) with a 10x objective, and analysed with ImageJ software (Fiji, version 2.14.0). Results were quantified as a ratio of TUNEL positive cells against the total number of cells.

Statistical Analysis

All graphs and statistical analyses were performed using either GraphPad Prism software 9 (GraphPad Software, USA) or Rstudio (RStudio Inc, version 1.2.5042).

All experiments were performed in at least four biological replicates, with 3 technical replicates performed per experiment, unless stated otherwise. All data were tested for normal distribution using the Shapiro-Wilk test. An unpaired student's t test was used to analyse the normally distributed data from two sample groups, while a one- or two-way ANOVA test was used to analyse three or more sample groups as appropriate.

Statistical significance was accepted when p-values were less than 0.05. All error bars are representative of mean (± SEM).

Supplementary References

1 Arnold, N. D. *et al.* A therapeutic antibody targeting osteoprotegerin attenuates severe experimental pulmonary arterial hypertension. *Nat Commun* **10**, 5183 (2019). https://doi.org:10.1038/s41467-019-13139-9

2 Pontes-Quero, S. *et al.* High mitogenic stimulation arrests angiogenesis. *Nat Commun* **10**, 2016 (2019). https://doi.org:10.1038/s41467-019-09875-7

3 Ainscough, A. J. *et al.* An organ-on-chip model of pulmonary arterial hypertension identifies a BMPR2-SOX17-prostacyclin signalling axis. *Commun Biol* **5**, 1192 (2022). https://doi.org:10.1038/s42003-022-04169-z

4 Ormiston, M. L. *et al.* Generation and Culture of Blood Outgrowth Endothelial Cells from Human Peripheral Blood. *J Vis Exp*, e53384 (2015). https://doi.org:10.3791/53384

5 Suh, K. S. *et al.* The organellular chloride channel protein CLIC4/mtCLIC translocates to the nucleus in response to cellular stress and accelerates apoptosis. *J Biol Chem* **279**, 4632-4641 (2004). https://doi.org:10.1074/jbc.M311632200
6 Wojciak-Stothard, B. Endothelial cell migration under flow. *Methods Mol Biol* **769**, 137-147 (2011). https://doi.org:10.1007/978-1-61779-207-6_10
7 Salibe-Filho, W. *et al.* Shear stress-exposed pulmonary artery endothelial cells fail to upregulate HSP70 in chronic thromboembolic pulmonary hypertension. *PLoS One* **15**, e0242960 (2020). https://doi.org:10.1371/journal.pone.0242960
8 Love, M. I., Huber, W. & Anders, S. Moderated estimation of fold change and dispersion for RNA-seq data with DESeq2. *Genome Biol* **15**, 550 (2014). https://doi.org:10.1186/s13059-014-0550-8
9 Welch, C. L. *et al.* Defining the clinical validity of genes reported to cause pulmonary arterial hypertension. *Genet Med* **25**, 100925 (2023). https://doi.org:10.1016/j.gim.2023.100925
10 Zhou, Y. *et al.* Metascape provides a biologist-oriented resource for the analysis of systems-level datasets. *Nat Commun* **10**, 1523 (2019). https://doi.org:10.1038/s41467-019-09234-6
11 Liao, Y., Wang, J., Jaehnig, E. J., Shi, Z. & Zhang, B. WebGestalt 2019: gene set analysis toolkit with revamped UIs and APIs. *Nucleic Acids Res* **47**, W199-W205 (2019). https://doi.org:10.1093/nar/gkz401
12 Rhodes, C. J. *et al.* RNA Sequencing Analysis Detection of a Novel Pathway of Endothelial Dysfunction in Pulmonary Arterial Hypertension. *Am J Respir Crit Care Med* **192**, 356-366 (2015). https://doi.org:10.1164/rccm.201408-1528OC
13 Hernandez, S. *et al.* Challenges and Opportunities for Immunoprofiling Using a Spatial High-Plex Technology: The NanoString GeoMx((R)) Digital Spatial Profiler. *Front Oncol* **12**, 890410 (2022). https://doi.org:10.3389/fonc.2022.890410
14 Guo, M. *et al.* Single Cell Multiomics Identifies Cells and Genetic Networks Underlying Alveolar Capillary Dysplasia. *Am J Respir Crit Care Med* **208**, 709-725 (2023). https://doi.org:10.1164/rccm.202210-2015OC
15 Wojciak-Stothard, B. *et al.* Aberrant chloride intracellular channel 4 expression contributes to endothelial dysfunction in pulmonary arterial hypertension. *Circulation* **129**, 1770-1780 (2014). https://doi.org:10.1161/CIRCULATIONAHA.113.006797
16 Albuquerque, M. L., Waters, C. M., Savla, U., Schnaper, H. W. & Flozak, A. S. Shear stress enhances human endothelial cell wound closure in vitro. *Am J Physiol Heart Circ Physiol* **279**, H293-302 (2000). https://doi.org:10.1152/ajpheart.2000.279.1.H293
17 Kabir, J., Lobo, M. & Zachary, I. Staurosporine induces endothelial cell apoptosis via focal adhesion kinase dephosphorylation and focal adhesion disassembly independent of focal adhesion kinase proteolysis. *Biochem J* **367**, 145-155 (2002). https://doi.org:10.1042/BJ20020665
