# Supplementary material for: KLF6 activation marks an angiogenic and apoptosis resistant endothelial phenotype in pulmonary arterial hypertension
Source: Commun Biol. 2026 Jun 15;9:846. doi: 10.1038/s42003-026-10493-5 (PMC13282386; doi:10.1038/s42003-026-10493-5)
Supplement: Supplementary file 3 — Description of Additional Supplementary Files [file 42003_2026_10493_MOESM3_ESM.pdf]

Description of Additional Supplementary File

File name: Supplementary data 1
Description: List of DEGs AdCTRLvsAdKLF6

File name: Supplementary data 2
Description: List of DEGs AdCTRLvsAdCTRL_hypoxia

File name: Supplementary data 3
Description: List of DEGs AdCTRLvsAdCTRL_+TNFalpha.

File name: Supplementary data 4
Description: List of DEGs AdCTRLvsAdKLF6+hypoxia

File name: Supplementary data 5
Description: List of DEGs AdCTRL+TNFalpha vs AdKLF6+TNFalpha.

File name: Supplementary data 6
Description: Description: List of DEGs AdCTRL-GFP vs AdKLF2- GFP

File name: Supplementary data 7
Description: List of DEGs AdCTRL vs AdKLF4

File name: Supplementary data 8
Description: List of KLF6 DEGs in PAH databases

File name: Supplementary data 9
Description: List of DEGS – comparison between nonplexiform vs plexiform lesions

File name: Supplementary data 10
Description: KLF6 DEGs shared with non-plexiform and plexiform DEGs

File name: Supplementary data 11
Description: List of DEGs – comparison Healthy controls vs PAH nonplexiform lesions

File name: Supplementary data 12
Description: Source data for all graphs
