# Supplementary material for: KLF6 activation marks an angiogenic and apoptosis resistant endothelial phenotype in pulmonary arterial hypertension
Source: Commun Biol. 2026 Jun 15;9:846. doi: 10.1038/s42003-026-10493-5 (PMC13282386; doi:10.1038/s42003-026-10493-5)
Supplement: Supplementary file 1 — Transparent Peer Review file [file 42003_2026_10493_MOESM1_ESM.pdf]

nature portfolio

Peer Review File

KLF6 Activation Marks an Angiogenic and Apoptosis Resistant Endothelial Phenotype in PAH

Corresponding Author: Professor Beata Wojciak-Stothard

This file contains all reviewer reports in order by version, followed by all author rebuttals in order by version.

Version 0:

Reviewer comments:

Reviewer #1

(Remarks to the Author)
The MS by Alharbi et al describes the role of KLF6 in the pulmonary vascular remodeling and PAH development. It showed that KLF6 overexpression promoted endothelial repair and angiogenesis in PAECs and proliferation of vascular smooth muscle cells in vitro, which can be inhibited by combination treatment of bosentan and imatinib. DisGeNET and spatial transcriptomics analysis of PAH lung vascular tissues revealed strong associations of KLF6 with human PAH. Increased expression of KLF6 was also shown in human PAH endothelium, PAH blood-derived endothelial progenitor cells, and pulmonary arterial cells in PAH associated with Alveolar Capillary Dysplasia. However, the study is descriptive, many data are not convincing, there is no direct evidence about the role KLF6 in PAH development and the dual role is not demonstrated.

Major comments
1. The expression of KLF6 in pulmonary vascular ECs in animal models is not explicitly demonstrated. The data did not support increased expression of KLF6 in MCT rats and SuHx rats, except in hypoxic mice. The increase showing in Fig 1B is minimal without any physiological significance. Immunostaining should be carried out to detect KLF6 expression in the 3 animal models at different time points. There are only a few cells expressing KLF6 in lungs of SuHx rats. What is the SuHx rat model used in the study, i.e., how many weeks of SuHx then normoxia? Are there occlusive vessels? Occlusive vessels should be assessed as they are more related to plexiform lesions PAH patients. The rat vessels in Fig S18 are quite normal.
2. Genetically modified mouse or rat models should be employed to determine whether overexpression or knockout of KLF6 affect pulmonary vascular remodeling and PAH development.
3. The evidence of KLF6 regulating endothelial repair and regeneration is not strong. The data do not support the hypothesis that exaggerated endothelial repair promotes vascular remodeling.
4. The vessels showing Fig 6E-G are more like normal vessels not occlusive vessels or plexiform lesions.
5. The data in Fig S13A showing minimal or neglectable induction of PASMC proliferation by AdKLF6.

Minor comments
6. Micrographs of Proliferation staining should be shown in Fig 2G.
7. Fig S16D, the cells in the last micrograph of PAH2 are not ECs, more likely fibroblasts (adventitial)

Reviewer #2

(Remarks to the Author)
General comments
In the present article, the authors explored whether Krüppel-like transcription factor 6 (KLF6) could play a role in pulmonary arterial hypertension (PAH) using in vivo (animal models) and in vitro approaches, as well as expression analyses in lung tissues and exploring datasets generated by them and others. The authors observed that KLF6 expression was increased in early stages of pulmonary hypertension (PH) in different animal models. In vitro, they showed that overexpressing KLF6 in pulmonary arterial endothelial cells resulted in endothelial repair and angiogenesis; in addition, they found that co-culture of PAECs overexpressing KLF6 with pulmonary arterial smooth muscle cells (PASMCs) promoted their proliferation. Finally, they analyzed DisGeNET and spatial transcriptomics of lung tissues from PAH patients and healthy controls and found

some associations between KLF6 and genes expressed in PAH. They also observed high expression levels of KLF6 in blood-derived endothelial progenitor cells and pulmonary arterial cells derived from PÄH patients. The manuscript is well written, but the structure and the reasoning is not always easy to follow. The reviewer would like to highlight the novelty of the manuscript. However, major issues would deserve to be carefully addressed, as detailed below.

Major comments
1. The title is not representative of the findings and looks like to the title of a review. The authors should consider to find a more appropriate title.
2. In the Abstract, the authors did not mention any research question/hypothesis, the aim of the study is not clearly described and there is no indication about the methodology.
3. Similarly, the hypothesis and aim of the study are crucially missing in the Introduction.
4. Why the authors used only one donor for PASMCs and 8 different donors for HPAECs? The authors should consider including at least 3 different donors for PASMCs.
5. Patients and healthy controls:
• The referee assumes that patients described in Table M2 and M8 are different, although this is not clear at all. The authors did not mention in which institution/hospital the patients described in Table M2 were recruited.
• Patients described in Table M2 are patients receiving double/triple therapies for long-term; this can seriously bias the results; it would be much more relevant to select patients at diagnosis.
• The mean pulmonary arterial pressure seems rather elevated for treated patients, and not compatible with patients with NYHA functional class I-II; the NYHA FC is missing for one of the patients.
• ECFCs: patients with PAH are much older than the controls. Are sure are the authors that the differences observed are not due to age? Could the authors comment on that? It would have been more relevant to select older donors, age-matched with the patients.
6. RNA quality: the authors did not check whether RNA used for qrt-PCR were degraded or not; ratio A260/A280 only indicates whether RNAs are contaminated by proteins or chemicals. Regarding RNA used for sequencing, they all had RIN numbers higher than 8, how these RIN numbers were determined? Why only RNA for sequencing had RIN numbers?
7. Statistics: only parametric test were used, which means that all parameters were normally distributed. Can the authors confirm that?
8. MCT rats: monocrotaline induces pulmonary hypertension in rats, but not pulmonary arterial hypertension; please correct. The authors should provide hemodynamics and Fulton index data from MCT-rats. Expression of KLF6 mRNAs is much lower in MCT rats compared to hypoxic mice, whereas PH is known to be much more severe in MCT rats. In addition, remodeling of pulmonary arteries is much more prominent in MCT rats compared to hypoxic mice (see Figure 1C). Can the authors comment on that?
9. The reviewer does not understand how the authors concluded that KLF6 is a key regulator of arterial identity. Could the authors elaborate on that?
10. It is not clear how the authors identify and define plexiform lesions. This should be better documented. Usually, plexiform lesions are also characterized by the presence of immune cells, which is not the case in the lesions shown in the manuscript.
11. Whether angiogenesis is beneficial or detrimental in the pathogenesis of PAH is under debate; for example different studies have shown that when bmpr2 signaling is impaired, angiogenesis is seriously altered. The authors did not really consider this aspect. Can they comment on that?
12. The reviewer is not really convinced by the relevance of the findings regarding alveolar capillary dysplasia and wonders whether they should be included within the results.
13. According to the “Integrated Single-Cell Atlas of Endothelial Cells of the Human Lung” (Schupp 2021 Circulation PMID 34030460), KLF6 is also highly expressed in immune cells including dendritic cells, B- and T-lymphocytes. Can the authors comment on that?
14. The authors mentioned in the discussion that targeting KLF6 could be a new therapeutic approach in PAH, but did not really elaborate on a therapeutic strategy. On one hand, they showed that in vivo in animal models, KLF6 is highly expressed in early phases of disease progression with a return to almost baseline values at later stages of disease progression. On the other hand, they show KLF6 is highly expressed in plexiform lesions and in a lesser extend in remodeled pulmonary artery arteries of PAH patients; this refers to advanced stages of the disease, as the reviewer assumes that are lungs collected at lung transplantation. Can the authors comment on this rather contradictory aspects?

Minor comments
1. In figure 6, scale and scale bar are missing in C and scale in A.
2. It is not clear whether EGM2 and ECGM2 media are different media or not.

Version 1:

Reviewer comments:

Reviewer #1

(Remarks to the Author)
The revision has only addressed some but not all of the comments. With the title change, the original major comment 2 is not required to be addressed. However, the data do not support the conclusion. For example, Fig 2G shows AdKLF6 decreased not increased HPAEC proliferation, thus it did not support the angiogenic effect of KLF6. KLF6-mediated apoptosis-resistance was not supported either (comment 1 below). The conclusion was overstated, there is no endothelial

regeneration, the repair may be ascribed to migration only.

Other comments,
1. FigS2, why there is a marked decrease of apoptosis in siRNA control+STS compared to control+STS? The Fig legend 9D) is confusing that treatment with TNFa or STS but only STS treatment was shown. Where is the data from TNFa treatment. These data failed to demonstrate KLF6 induces apoptosis-resistance. Overexpression of KLF6 should be carried out. However, Fig1H shows only 30% reduction of apoptosis but still markedly greater (about 10-fold) than control .
2. Fig S12B is not representative, there is no + nuclei in AdKLF6+B+I group.
3. Fig. S17B is unlikely a plexiform lesion. Please explain why KLF6 was highly expressed in EC in one vessel but not in other vessels (both A and B).
4. Fig. S18, why normal lung does not have SMA staining and also KLF6 expression was totally absent. The patient data does not support the conclusion as it is from patients with tuberculosis. Quantification is needed.
5. Fig S19, Hypoxic mouse is not a PAH model. Fig S19B shows only minor increase of KLF6 mRNA (20%) in MCT rat lungs, not impressive. It seems KLF6 expression is more likely regulated at the protein level not transcription under PH pathological condition. If this is the case, all transcriptomic data about KLF6 expression may be of concern.
6. The abstract and text talk about experimental PAH, but hypoxic mouse is not a PAH model.

Reviewer #2

(Remarks to the Author)
General comments
Several issues have been addressed by the authors. The reviewer would like to acknowledge the efforts made by the authors to improve their manuscript, including a couple of additional experiments. However, there are a couple of issues that should be further clarified, as detailed below.
Major comments
1. The hypothesis is still missing in the Introduction.
2. Patients described in Table M2 are patients receiving double/triple therapies for long-term; this can seriously bias the results; it would be much more relevant to select patients at diagnosis. The authors answered: “Given that these cells were isolated from patients' blood and then cultured for 2–3 weeks with clonal selection and regular media changes, it is unlikely that any effects of the drugs would persist in culture”. The reviewer would like to know whether the authors have any concrete clue that drugs cannot have any effects on cells. Actually, it is not known whether long-term exposition to vasodilators may affect the cells or not. Longitudinal studies should be performed to assert it. This should be better highlighted in the discussion.
3. The reviewer is still puzzled by the elevated values of the mean PAP of treated patients. Are the authors sure that PAP values they show are mean PAP and not systolic PAP? Could the authors mention whether the PAP values are measured by right heart catheterization or transthoracic echocardiography? Can the authors provide other hemodynamic data such cardiac index, pulmonary vascular resistance? Could the authors mention how long the patients were treated by PAH drugs.
4. The authors did not answer the question regarding the RNA quality. How the authors can be sure that the RNA is not degraded?
5. The authors mentioned that they used archived lung samples from MCT and SuHx rats. The authors should provide hemodynamics and Fulton index data from these rats to illustrate the severity of PH.
6. The authors did not comment about the previous reviewer’s question:” Expression of KLF6 mRNAs is much lower in MCT rats compared to hypoxic mice, whereas PH is known to be much more severe in MCT rats. In addition, remodelling of pulmonary arteries is much more prominent in MCT rats compared to hypoxic mice (see Figure 1C)”. Can the authors comment on that and clarify that point?
7. “The reviewer does not understand how the authors concluded that KLF6 is a key regulator of arterial identity”. The answer of the authors is rather brief. Could the authors further elaborate on that?
8. “Whether angiogenesis is beneficial or detrimental in the pathogenesis of PAH is under debate”. The answer of the authors is rather brief. The authors should consider to better document the statement: “Inhibition of BMPR2 compromises angiogenic responses”. Please see Gu 2017 Cell Stem Cell; Tielemans 2023 Pulm Circ; Spiekerkoetter 2013 J Clin Invest.
9. “The reviewer is not really convinced by the relevance of the findings regarding alveolar capillary dysplasia and wonder whether they should be included within the results.” The authors answered that they would like to retain this part. The reviewer may understand but the authors should be more convincing and better elaborate on that.
10. The authors answered that “Predicting treatment outcomes might be too speculative, given that the role of angiogenesis in PAH is not fully understood”. Could the authors consider that KLF6 may have a dual role in early stages versus advanced stages of the diseases? What about the discrepancies between MCT rats, SuHx rats and patients?

Version 2:

Reviewer comments:

Reviewer #1

(Remarks to the Author)
The 2nd revision has addressed most of the comments. However, comments 1 is not appropriately addressed. New experiment with additional data needs to determine whether there is a marked decrease of apoptosis in siRNA control+STS compared to control+STS (Fig S2). The current data shows a 40% decrease. The answer about statistical significance is not

sufficient. Additionally, the 30% decrease of apoptosis by AdKLF6 (Fig 1H) is not strong to support the conclusion about KLF6 activation marking of the apoptosis-resistant endothelial phenotype in PAH.

Reviewer #2

(Remarks to the Author)

Open Access This Peer Review File is licensed under a Creative Commons Attribution 4.0 International License, which permits use, sharing, adaptation, distribution and reproduction in any medium or format, as long as you give appropriate credit to the original author(s) and the source, provide a link to the Creative Commons license, and indicate if changes were made.
In cases where reviewers are anonymous, credit should be given to 'Anonymous Referee' and the source.
The images or other third party material in this Peer Review File are included in the article’s Creative Commons license, unless indicated otherwise in a credit line to the material. If material is not included in the article’s Creative Commons license and your intended use is not permitted by statutory regulation or exceeds the permitted use, you will need to obtain permission directly from the copyright holder.
To view a copy of this license, visit https://creativecommons.org/licenses/by/4.0/

We thank the Reviewers for their insightful comments.

In the revised manuscript, all changes are highlighted in yellow.

Reviewers' comments:

Reviewer #1 (Remarks to the Author):
…However, the study is descriptive, many data are not convincing, there is no direct evidence about the role KLF6 in PAH development and the dual role is not demonstrated.
Major comments
1. The expression of KLF6 in pulmonary vascular ECs in animal models is not explicitly demonstrated. The data did not support increased expression of KLF6 in MCT rats and SuHx rats, except in hypoxic mice. The increase showing in Fig 1B is minimal without any physiological significance. Immunostaining should be carried out to detect KLF6 expression in the 3 animal models at different time points. There are only a few cells expressing KLF6 in lungs of SuHx rats. What is the SuHx rat model used in the study, i.e., how many weeks of SuHx then normoxia? Are there occlusive vessels? Occlusive vessels should be assessed as they are more related to plexiform lesions PAH patients. The rat vessels in Fig S18 are quite normal.

2. Genetically modified mouse or rat models should be employed to determine whether overexpression or knockout of KLF6 affect pulmonary vascular remodelling and PAH development.

Response: Our study focuses on the potential involvement of KLF6 in the pathology of human PAH. To enhance clarity, we have reorganized the manuscript to place greater emphasis on the responses of human pulmonary endothelial cells and on human PAH.

We have added new immunofluorescent staining of lung sections from MCT rats (28 days post-MCT) and Sugen/hypoxia rats (please see revised Supplementary Figure 18), which demonstrates the accumulation of KLF6$^+$ cells. These cells exhibit some degree of structural organization, although not to the same extent as observed in human PAH. This aligns with the limited ability of rodent models to replicate human plexiform-like arteriopathy[1]. We state this in Discussion on p.15

The data showing early changes in KLF6 mRNA levels in whole-lung extracts from hypoxic mice and MCT rats—previously presented in Figure 1—are now shown in Supplementary Figure S19. We used archived samples from MCT and Sugen/hypoxia rats, with ethical approvals and phenotypic details described in the original publication[2]. The information regarding animal experimentation is now provided in the main manuscript on page 26 and in Supplemental Methods section on page 59-60.

We have also moderated our statements regarding the dual role of KLF6 in PAH pathogenesis and revised the manuscript title to better reflect the experimental work conducted.

Our study highlights the potential role of KLF6 in the pathology of human PAH, as investigated through *in vitro* experiments, transcriptomic analyses, and validation using human PAH lung tissues and PAH datasets. Determining the precise role of KLF6 in disease development would require extensive studies using endothelium-specific, inducible KLF6 knockout or knock-in models, which are beyond the scope of the current investigation

3. The evidence of KLF6 regulating endothelial repair and regeneration is not strong. The data do not support the hypothesis that exaggerated endothelial repair promotes vascular remodelling.

RESPONSE: KLF6 is known to promote endothelial repair and angiogenesis, largely through enhancement of TGF-β signalling [3 4,5]. Our data strongly support this conclusion. The analysis of KLF6-induced transcriptomic changes clearly demonstrates regulation of genes associated with angiogenesis and vascular repair, and we have identified a distinct KLF6 transcriptomic signature in PAH vasculature. Multiple *in vitro* functional assays further confirm the role of KLF6 in promoting endothelial survival and angiogenesis.

In addition, we have shown that KLF6 localizes to cells expressing endothelial markers within remodelled vascular tissues, and that KLF6 is significantly upregulated in PAH-derived endothelial colony-forming cells (ECFCs) compared to healthy controls. Taken together with the activation of KLF6 under vascular stress conditions, these observations raise the possibility that KLF6 contributes to vascular repair and remodelling in the PAH lung.

While genetically modified mouse or rat models—particularly endothelium-specific, inducible knockout animals—would be valuable for defining the temporal dynamics of KLF6 activation and its role at different disease stages, such an investigation is extensive and beyond the scope of the current study

4. The vessels showing Fig 6E-G are more like normal vessels not occlusive vessels or plexiform ***lesions.***

RESPONSE: The vessels depicted in Figure 6 E, F, G come from the large plexiform lesion shown in Fig. 6C. We have replaced one of the images in Fig 6E and provided additional images of sections stained for vWF, α-SMA and KLF6 in the Supplementary Figure 16 for further clarification.

5. The data in Fig S13A showing minimal or neglectable induction of PASMC proliferation by AdKLF6.

RESPONSE: A ~ 2.5-fold increase in PASMC proliferation is comparable with the effects of PAH-relevant stressors such as BMPR2 knockdown combined with BMP6[6] and difference in basal proliferation rate between healthy and IPAH PASMCs[7]

Minor comments
6. Micrographs of Proliferation staining should be shown in Fig 2G.

RESPONSE: We now show images of proliferation staining in Fig 2G

7. Fig S16D, the cells in the last micrograph of PAH2 are not ECs, more likely fibroblasts (adventitial)

RESPONSE: WE have provided more representative images of KLF6 localization in IPAH2 lung in Figure S15D.

While KLF6 activation was predominantly observed in PAH endothelial cells, we cannot exclude the possibility that other cell types might also be involved. For example, both human and murine macrophages express high levels of KLF6, which regulates immune responses[8]. However, human TB lung, that typically presents high accumulation of immune cells, including T-lymphocytes and macrophages[9], did not reproduce the pattern of KLF6$^+$ staining seen in PAH lung.

We comment on potential involvement of other cell types in Discussion on page 15

Reviewer #2 (Remarks to the Author):

*….The manuscript is well written, but the **structure and the reasoning is not always easy to follow.** The reviewer would like to highlight the novelty of the manuscript. However, major issues would deserve to be carefully addressed, as detailed below.*

Major comments

1. The title is not representative of the findings and looks like to the title of a review. The authors should consider to find a more appropriate title.

RESPONSE: To enhance clarity, we have reorganized the manuscript to place greater emphasis on the responses of human pulmonary endothelial cells and on human PAH.

We hope that the new title "KLF6 Activation Marks an Angiogenic and Apoptosis Resistant Endothelial Phenotype in PAH", is more reflective of the study content.

2. In the Abstract, the authors did not mention any research question/hypothesis, the aim of the study is not clearly described and there is no indication about the methodology.

3. Similarly, the hypothesis and aim of the study are crucially missing in the Introduction.

RESPONSE: The hypothesis and aim are now stated in the Abstract and Introduction.

In the Abstract (shortened to 150 words): Krüppel-like factor 6 (KLF6) promotes arterial neointima formation and we hypothesized that KLF6 contributes to endothelial dysfunction in PAH.

In the Introduction: We aimed to examine effects of PAH-associated hypoxic and inflammatory stressors on KLF6 expression *in vitro* and *in vivo* and characterise functional and transcriptional effects of KLF6 activation in pulmonary vascular endothelium.

4. Why the authors used only one donor for PASMCs and 8 different donors for HPAECs? The authors should consider including at least 3 different donors for PASMCs.

RESPONSE: We have provided new data using cells from 3 different donors to new Figure S12 (old Figure S13). Donor list has been included in the Table M2 on page 40 of Supplement.

5. Patients and healthy controls:

• The referee assumes that patients described in Table M2 and M8 are different, although this is not clear at all. The authors did not mention in which institution/hospital the patients described in Table M2 were recruited.

Response: Donor information for healthy donors of PASMCs listed in Table M2 has been provided by the company Promocell.

Table M3 lists healthy and PAH donors of endothelial colony forming cells (ECFCs) obtained from patient blood collected at Pulmonary Hypertension Clinic, Hammersmith Hospital, Imperial College London. We have added this information in Supplement on page 40 (in sub-chapter " Peripheral blood mononuclear cells (PBMCs) and endothelial colony forming cells (ECFCs)") and on page 17 in the main manuscript.

• Patients described in Table M2 are patients receiving double/triple therapies for long-term; this can seriously bias the results; it would be much more relevant to select patients at diagnosis.

Response: We derived ECFCs from the peripheral blood of patients undergoing treatment (listed in Table M3 on page 41-42 in Supplement). Given that these cells were isolated from patients' blood and then cultured for 2–3 weeks with clonal selection and regular media changes, it is unlikely that any effects of the drugs would persist in culture. We comment on this potential limitation in Discussion on page 15.

• The mean pulmonary arterial pressure seems rather elevated for treated patients, and not compatible with patients with NYHA functional class I-II; the NYHA FC is missing for one of the patients.

Response: We thank the Reviewer for pointing out this mistake – we have now made corrections in the Supplementary Table M3: 3 patients were from WHO FC II and 2 from WHO FC III.

• ECFCs: patients with PAH are much older than the controls. Are sure are the authors that the differences observed are not due to age? Could the authors comment on that? It would have been more relevant to select older donors, age-matched with the patients.

Response: We have made the best effort to age-match donors and used donors of a similar age range. However, as noted by the Reviewer, healthy donors were on average younger, and this is mentioned as a potential limitation in Discussion on page 15

6. RNA quality: the authors did not check whether RNA used for qrt-PCR were degraded or not; ratio A260/A280 only indicates whether RNAs are contaminated by proteins or chemicals. Regarding RNA used for sequencing, they all had RIN numbers higher than 8, how these RIN numbers were determined? Why only RNA for sequencing had RIN numbers?

Response: We checked both the quality and concentration twice (duplicates), using the Agilent tapestation for the quality 'RIN' and then crossed referenced the concentrations on the LifeTech Qubit. These two together gives us a very good understanding of the sample's quality and concentration.

7. Statistics: only parametric test were used, which means that all parameters were normally distributed. Can the authors confirm that?

Response: We have confirmed normal distribution of data before choosing parametric tests. We mention this in the Statistical analysis paragraph on page 26.

8. *MCT rats: monocrotaline induces pulmonary hypertension in rats, but not pulmonary arterial hypertension; please correct*.

Response: We thank the Reviewer for spotting this mistake - it has now been corrected

The authors should provide hemodynamics and Fulton index data from MCT-rats. Expression of KLF6 mRNAs is much lower in MCT rats compared to hypoxic mice, whereas PH is known to be much more

severe in MCT rats. In addition, remodelling of pulmonary arteries is much more prominent in MCT rats compared to hypoxic mice (see Figure 1C). Can the authors comment on that?

We used archived lung samples from MCT and Sugen/hypoxia rats[2]. The information regarding the source of animal tissues, ethical agreements and animal phenotyping has been provided on pages 25-26 in the main manuscript and on pages 59-60 in Supplemental Methods.

9. The reviewer does not understand how the authors concluded that KLF6 is a key regulator of arterial identity. Could the authors elaborate on that?

Response: We have clarified which genes are common regulators of endothelial function and which are specifically associated with arterial identity (Abstract and Discussion on page 8)

…Notably, KLF6 enhanced expression of several key regulators of endothelial repair and angiogenesis, including *ERG[10], ANG-2[11], TIE2[11], BMPR2[12], KDR[13], HES1[14]*, *CDH5[10], PECAM1[15]*, *FLT1[16]* and arterial identity regulator *SOX17[17]…*

10. It is not clear how the authors identify and define plexiform lesions. This should be better documented. Usually, plexiform lesions are also characterized by the presence of immune cells, which is not the case in the lesions shown in the manuscript.

RESPONSE: Plexiform lesions were identified based on established structural characteristics using α-SMA and CD31 immunostaining, along with spatial morphology consistent with published definitions[18,19]. Specifically, regions were selected where there was evidence of endothelial cell proliferation around remodelled vessels lacking a continuous media. These selection criteria are now described in Methods on page 11.

To further support the identity of these regions as plexiform lesions, we performed differential gene expression (DEG) and pathway analysis (Metascape) comparing plexiform versus non-plexiform regions. These analyses revealed upregulation of genes and pathways related to ECM remodelling (e.g., *COL18A1, SERPINE1*), TGF-β signalling (e.g., *LTBP1, HTRA1*), angiogenesis (e.g., *ECE1, AQP1, MCAM*), apoptosis, and some indirect inflammatory markers (e.g *IRAG1, LGALS1*), consistent with spatial profiling results reported by Tuder et al. (2024) [20]. Metascape pathway analysis of upregulated DEGs from comparison between plexiform vs non-plexiform vessels in spatial transcriptomics is provided in Figure R1 below.

These findings align with the complex remodelling and endothelial proliferation described in plexiform lesions and support our morphological classification. While immune-related genes were not among the most differentially expressed, this may reflect spatial resolution limits, or the stage of lesion captured.

Metascape Pathway Analysis

DEGs in plexiform regions compared to non-plexiform vessels

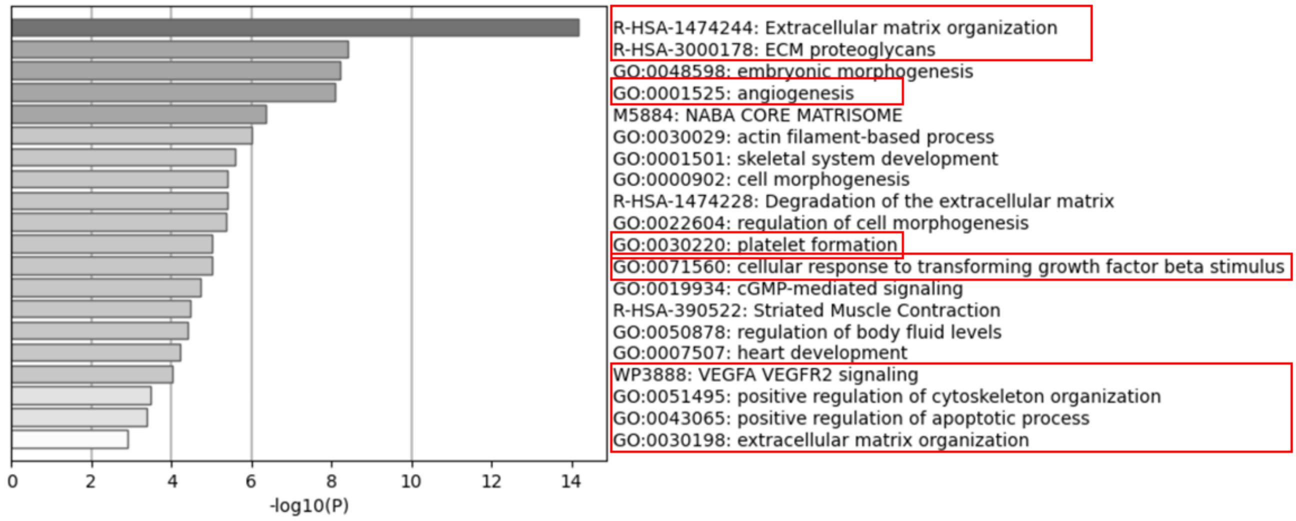

Figure R1: Metascape pathway analysis of upregulated DEGs from comparison between plexiform vs non-plexiform vasculature selected for spatial transcriptomics. *30 ROIs/lung in sections from 6 PAH patients*

11. Whether angiogenesis is beneficial or detrimental in the pathogenesis of PAH is under debate; for example different studies have shown that when bmpr2 signaling is impaired, angiogenesis is seriously altered. The authors did not really consider this aspect. Can they comment on that?

Response:

To address the Reviewer's comment, we have included the following statement in Discussion on page 16-17:

"…It is important to note that the effects of KLF6 activation or inhibition are likely to depend on the stage of the disease and the cellular context, similar to its target, BMPR2. While inhibition of BMPR2 compromises angiogenic responses[12], it can also promote endothelial proliferation and angiogenesis in the presence of BMP9[21]. Given that the role of angiogenesis in PAH remains under debate, determining the potentially beneficial or detrimental effects of KLF6 activation will require further investigation…"

12. The reviewer is not really convinced by the relevance of the findings regarding alveolar capillary

Response: We would like to retain this part of the Results section, as we believe it provides important context for pulmonary diseases associated with vascular remodelling.

13. According to the "Integrated Single-Cell Atlas of Endothelial Cells of the Human Lung" (Schupp 2021 Circulation PMID 34030460), KLF6 is also highly expressed in immune cells including dendritic cells, B- and T-lymphocytes. Can the authors comment on that?

: While KLF6 activation was predominantly observed in PAH endothelial cells, we cannot exclude the possibility that other cell types are also involved. For example, both human and murine macrophages express high levels of KLF6, which plays a role in regulating immune responses[8]. However, human TB lung, from disease that typically presents high accumulation of immune cells,

including T-lymphocytes and macrophages[9], did not reproduce the pattern of KLF6$^+$ staining seen in PAH lung.

We comment on potential involvement of other cell types in Discussion on page 15

14. The authors mentioned in the discussion that targeting KLF6 could be a new therapeutic approach in PAH, but did not really elaborate on a therapeutic strategy. On one hand, they showed that in vivo in animal models, KLF6 is highly expressed in early phases of disease progression with a return to almost baseline values at later stages of disease progression. On the other hand, they show KLF6 is highly expressed in plexiform lesions and in a lesser extend in remodeled pulmonary artery arteries of PAH patients; this refers to advanced stages of the disease, as the reviewer assumes that are lungs collected at lung transplantation. Can the authors comment on this rather contradictory aspects?

RESPONSE: Predicting treatment outcomes might be too speculative, given that the role of angiogenesis in PAH is not fully understood. This limitation is discussed on page 16-17:

It is important to note that the effects of KLF6 activation or inhibition are likely to depend on the stage of the disease and the cellular context, similar to its target, BMPR2. While inhibition of BMPR2 compromises angiogenic responses[12], it can also promote endothelial proliferation and angiogenesis in the presence of BMP9[21]. Given that the role of angiogenesis in PAH remains under debate, determining the potentially beneficial or detrimental effects of KLF6 activation will require further investigation.

Minor comments

1. In figure 6, scale and scale bar are missing in C and scale in A.

Response: This has now been corrected

2. It is not clear whether EGM2 and ECGM2 media are different media or not.

Response: EGM2 and ECGM2 are the same medium, we have corrected to EGM2

1 Boucherat, O., Agrawal, V., Lawrie, A. & Bonnet, S. The Latest in Animal Models of Pulmonary Hypertension and Right Ventricular Failure. *Circ Res* **130**, 1466-1486 (2022). https://doi.org:10.1161/CIRCRESAHA.121.319971

2 Arnold, N. D. *et al.* A therapeutic antibody targeting osteoprotegerin attenuates severe experimental pulmonary arterial hypertension. *Nat Commun* **10**, 5183 (2019). https://doi.org:10.1038/s41467-019-13139-9

3 Gallardo-Vara, E. *et al.* Transcription factor KLF6 upregulates expression of metalloprotease MMP14 and subsequent release of soluble endoglin during vascular injury. *Angiogenesis* **19**, 155-171 (2016). https://doi.org:10.1007/s10456-016-9495-8

4 Botella, L. M. *et al.* Transcriptional activation of endoglin and transforming growth factor-beta signaling components by cooperative interaction between Sp1 and KLF6: their potential role in the response to vascular injury. *Blood* **100**, 4001-4010 (2002). https://doi.org:10.1182/blood.V100.12.4001

5 Garrido-Martin, E. M. *et al.* Vascular injury triggers Kruppel-like factor 6 mobilization and cooperation with specificity protein 1 to promote endothelial activation through upregulation of the activin receptor-like kinase 1 gene. *Circ Res* **112**, 113-127 (2013). https://doi.org:10.1161/CIRCRESAHA.112.275586

6 Hurst, L. A. *et al.* TNFalpha drives pulmonary arterial hypertension by suppressing the BMP type-II receptor and altering NOTCH signalling. *Nat Commun* **8**, 14079 (2017). https://doi.org:10.1038/ncomms14079
7 Wu, K. *et al.* Endothelial platelet-derived growth factor-mediated activation of smooth muscle platelet-derived growth factor receptors in pulmonary arterial hypertension. *Pulm Circ* **10**, 2045894020948470 (2020). https://doi.org:10.1177/2045894020948470
8 Syafruddin, S. E., Mohtar, M. A., Wan Mohamad Nazarie, W. F. & Low, T. Y. Two Sides of the Same Coin: The Roles of KLF6 in Physiology and Pathophysiology. *Biomolecules* **10** (2020). https://doi.org:10.3390/biom10101378
9 Flynn, J. L. & Chan, J. Immune cell interactions in tuberculosis. *Cell* **185**, 4682-4702 (2022). https://doi.org:10.1016/j.cell.2022.10.025
10 Birdsey, G. M. *et al.* Transcription factor Erg regulates angiogenesis and endothelial apoptosis through VE-cadherin. *Blood* **111**, 3498-3506 (2008). https://doi.org:10.1182/blood-2007-08-105346
11 Felcht, M. *et al.* Angiopoietin-2 differentially regulates angiogenesis through TIE2 and integrin signaling. *J Clin Invest* **122**, 1991-2005 (2012). https://doi.org:10.1172/JCI58832
12 Hiepen, C. *et al.* Endothelial tip-cell position, filopodia formation and biomechanics require BMPR2 expression and signaling. *Commun Biol* **8**, 21 (2025). https://doi.org:10.1038/s42003-024-07431-8
13 Ferrara, N. Role of vascular endothelial growth factor in the regulation of angiogenesis. *Kidney Int* **56**, 794-814 (1999). https://doi.org:10.1046/j.1523-1755.1999.00610.x
14 Magesh, V. *et al.* HES1 in cancer: a key player in tumorigenesis and its prognostic significance. *Mol Genet Genomics* **300**, 49 (2025). https://doi.org:10.1007/s00438-025-02259-1
15 Park, S., Sorenson, C. M. & Sheibani, N. PECAM-1 isoforms, eNOS and endoglin axis in regulation of angiogenesis. *Clin Sci (Lond)* **129**, 217-234 (2015). https://doi.org:10.1042/CS20140714
16 Wazan, L. E., Widhibrata, A. & Liu, G. S. Soluble FLT-1 in angiogenesis: pathophysiological roles and therapeutic implications. *Angiogenesis* **27**, 641-661 (2024). https://doi.org:10.1007/s10456-024-09942-8
17 Simmons Beck, R., Liang, O. D. & Klinger, J. R. Light at the ENDothelium-role of Sox17 and Runx1 in endothelial dysfunction and pulmonary arterial hypertension. *Front Cardiovasc Med* **10**, 1274033 (2023). https://doi.org:10.3389/fcvm.2023.1274033
18 Jonigk, D. *et al.* Plexiform lesions in pulmonary arterial hypertension composition, architecture, and microenvironment. *Am J Pathol* **179**, 167-179 (2011). https://doi.org:10.1016/j.ajpath.2011.03.040
19 Tuder, R. M. *et al.* Expression of angiogenesis-related molecules in plexiform lesions in severe pulmonary hypertension: evidence for a process of disordered angiogenesis. *J Pathol* **195**, 367-374 (2001). https://doi.org:10.1002/path.953
20 Tuder, R. M. *et al.* Digital Spatial Profiling Identifies Distinct Molecular Signatures of Vascular Lesions in Pulmonary Arterial Hypertension. *Am J Respir Crit Care Med* **210**, 329-342 (2024). https://doi.org:10.1164/rccm.202307-1310OC
21 Theilmann, A. L. *et al.* Endothelial BMPR2 Loss Drives a Proliferative Response to BMP (Bone Morphogenetic Protein) 9 via Prolonged Canonical Signaling. *Arterioscler Thromb Vasc Biol* **40**, 2605-2618 (2020). https://doi.org:10.1161/ATVBAHA.119.313357

Response to Reviewers COMMSBIO-25-1136A

We would like to thank the the Reviewers for the time and effort devoted to evaluating our manuscript and for the constructive feedback provided.

In response to the Reviewers' comments, we have

1. Removed the hypoxia-induced mouse model and transcriptomic data showing changes in KLF6 mRNA levels in the lungs of hypoxic mice and MCT rats.
2. Expanded and reorganized the Discussion section to strengthen the narrative flow and improve the coherence of the results presentation.
3. Added further information on the rat PAH models and ECFC donors.
4. Included a semi-quantitative evaluation of KLF6 protein expression in human lungs as well as in MCT and SuHx rat lungs.
5. Added a new figure (Fig. S20) showing KLF6 expression levels in endothelial tip cells in the healthy human lung.
6. Modified the summary diagram.

A detailed response to specific queries raised by the Reviewers is below:

Reviewer #1 (Remarks to the Author):

The revision has only addressed some but not all of the comments. With the title change, the original major comment 2 is not required to be addressed. However, the data do not support the conclusion.

For example, Fig 2G shows AdKLF6 decreased not increased HPAEC proliferation, thus it did not support the angiogenic effect of KLF6. KLF6-mediated apoptosis-resistance was not supported either (comment 1 below). The conclusion was overstated, there is no endothelial regeneration, the repair may be ascribed to migration only.

Response: The endothelial wound healing in vitro is predominantly dependent on cell motility[1] and so, the effects of KLF6 observed here are likely to result from enhanced endothelial migratory activity. We now comment on this in Results on page 6.

Angiogenesis is a complex process involving timely coordinated changes in cell-cell adhesion, migration, proliferation and matrix degradation. Whilst most factors considered as "pro-angiogenic" simultaneously promote or inhibit both proliferation and sprouting of endothelial cells, several recent and higher resolution studies are proposing the existence of pro-angiogenic mechanisms that have an opposing effect on proliferative and migratory cellular responses. This is elegantly argued in the review [2].

To clarify this issue, we extended Discussion on page 13:

"…Interestingly, and perhaps counterintuitively, KLF6 also reduced the expression of genes involved in cell cycle progression and inhibited PAEC proliferation *in vitro*. Angiogenesis is a complex process involving a series of temporally and spatially controlled events involving dissolution and re-formation of cell-cell

adhesions, cell migration and proliferation. Whilst the common perception is that "pro-angiogenic" factors should promote both proliferation and sprouting/migration of endothelial cells, recent studies revealed the existence of pro-angiogenic mechanisms that have an opposing effect on proliferative and migratory cellular responses[2]. For example, mitogen-induced cell cycle arrest is observed primarily in tip cells at the angiogenic front, while an increase in proliferating ECs can be observed in more mature and quiescent vascular regions[3]

The observation of differential regulation of migration and proliferation in vascular tip cells is particularly intriguing, considering that the highest expression of KLF6 in the healthy human lung endothelium is observed in endothelial tip cells[3] (Figure shown below has been included in the manuscript as Supplemental Figure S21). The subtype of endothelial cells showing elevated KLF6 expression in PAH remains to be established but KLF6+ arterial endothelial cells seen in the occluded PAH vasculature may potentially originate from pulmonary-venous ECs and capillary ECs[4 5].

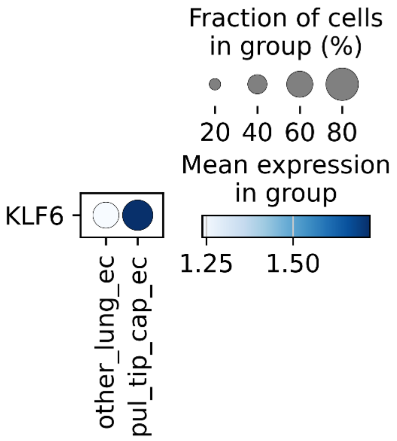

Figure S21. *Dot plot representation of KLF6 expression in lung tip endothelial cells (pul_tip_cap_ec) compared to other merged lung blood vessel endothelial cell populations (other_lung_ec) using single-cell RNA-seq data acquired from Barnett et al. 2024 Nature Medicine*[3].

Our transcriptomic data demonstrate a clear involvement of KLF6 in the regulation of pathways involved in vascular repair and angiogenesis and the spatial transcriptomic analysis of PAH lung vascular tissues uncovered a significant enrichment of KLF6-regulated genes in processes that drive vascular repair and angiogenesis.

Other comments,

1. Fig S2, why there is a marked decrease of apoptosis in siRNA control+STS compared to control+STS?

Response: There is no statistically significant decrease of apoptosis in siRNA control+STS compared to control+STS

The Fig legend 9D) is confusing that treatment with TNFa or STS but only STS treatment was shown. Where is the data from TNFa treatment.

Response: We have provided a clearer explanation which treatment was used in panels (C) and (D) in Figure S2: the inflammatory effect (NFκB activity) was

evaluated using TNF-α, whereas the apoptotic effect was evaluated using staurosporine (STS).

2. These data failed to demonstrate KLF6 induces apoptosis-resistance. Overexpression of KLF6 should be carried out. However, Fig1H shows only 30% reduction of apoptosis but still markedly greater (about 10-fold) than control .

Response: Our experiments provide a "proof of concept" that KLF6 overexpression attenuates apoptosis, whilst KLF6 silencing has an opposite effect. 30% reduction in STS-induced apoptosis, seen in in KLF6-overexpressing HPAECs in Figure 1H was a statistically significant effect.

Effects of KLF6 silencing were studied in flow-exposed HPAECs, as the basal KLF6 expression in unstimulated, quiescent HPAECs was too low to produce a measurable effect and flow exposure elevates basal levels of KLF6 expression (Fig. 1B).

To improve clarity, additional information has been provided in Supplemental Methods on page 43 and 60 and figure legends in Figures S2 and S4.

3. Fig S12B is not representative, there is no + nuclei in AdKLF6+B+I group.

Response: We have provided a more representative image for no + nuclei in AdKLF6+B+I group.

4. Fig. S17B is unlikely a plexiform lesion.

Response: Figure S17 shows a fragment of a vascular channel contained within the larger lesion illustrated in Figures S15 and S16. We now clarify this in the figure legend.
The lesion shown in Figures S15 and S16 demonstrates complex, glomeruloid-like vascular structures surrounding a remodelled pulmonary artery, consistent with the accepted definition of a plexiform lesion[6].

Please explain why KLF6 was highly expressed in EC in one vessel but not in other vessels (both A and B).

Response: The reasons why all vessels are not equally affected by the disease, and why there is heterogeneity in KLF6 expression amongst endothelial cells, remains unclear. We comment on this in Discussion on page 14:

"…Human PAH lung sections showed accumulation of $KLF6^+$ vWF^+ ERG^+ cells within vascular channels in plexiform lesions and, to a lesser extent, in other parts of the remodelled lung. This selective overexpression of KLF6 in PAH endothelium is corroborated by recent data showing that only 2 out of 8 endothelial cell clusters in PAH lung display a pro-angiogenic phenotype[7]… "

and later, on page 15: "….Establishing the identity of KLF6+ cells through targeted functional genetics with single-cell multiomics will be vital to understand the role of KLF6 in the vascular remodelling in PAH."

5. Fig. S18, why normal lung does not have SMA staining and also KLF6 expression was totally absent. The patient data does not support the conclusion as it is from patients with tuberculosis. Quantification is needed.

Response: We have provided two new images showing αSMA staining and KLF6 staining in the untreated lung. The images focus on peripheral lung tissues to emphasize an increase in small vessel muscularization happening in the disease.

We have also provided a semi-quantitative evaluation of changes in KLF6 expression in MCT and SuHx lungs in new Figure S19B (fluorescence intensity; fold-change of control). Method is described in Supplemental Methods on page 55.

Lung tissue from a patient with tuberculosis, an infectious lung disease characterised by intense inflammation and lung-parenchymal damage[8] was used as a disease control for human PAH lung. To improve clarity, this image has been moved to Figure S16 showing human PAH lung.

Fig S19, Hypoxic mouse is not a PAH model. Fig S19B shows only minor increase of KLF6 mRNA (20%) in MCT rat lungs, not impressive. It seems KLF6 expression is more likely regulated at the protein level not transcription under PH pathological condition. If this is the case, all transcriptomic data about KLF6 expression may be of concern.

Response: We have removed the hypoxic mouse PH model from the manuscript and now refer to the MCT rat and Sugen/hypoxia models as preclinical models of PAH. The transcriptomic data from MCT rats have also been removed, as we do not have sufficient data to establish correlations between KLF6 expression levels and the degree of vascular remodelling at different stages of disease progression. Given the interspecies differences and distinct pathogenic mechanisms among these models, a meaningful comparative analysis would be difficult to perform.

6. The abstract and text talk about experimental PAH, but hypoxic mouse is not a PAH model.

We acknowledge that referring to the chronic hypoxia-induced mouse model as a model of PAH may be inappropriate. The model has been removed from the manuscript.

A mitigating argument might be that in many publications, animal models used to investigate mechanisms contributing to PAH - including non-invasive, invasive, gene-editing, and multifactorial models, each representing different aspects of the disease - are collectively referred to as models of PAH [9,10]

Reviewer #2 (Remarks to the Author):

General comments

Several issues have been addressed by the authors. The reviewer would like to acknowledge the efforts made by the authors to improve their manuscript, including a couple of additional experiments. However, there are a couple of issues that should be further clarified, as detailed below.

Major comments

1. The hypothesis is still missing in the Introduction.

Response: We have added “We hypothesized that KLF6 activation is a feature of vascular pathology in PAH” in Introduction on page 4

2. Patients described in Table M2 are patients receiving double/triple therapies for long-term; this can seriously bias the results; it would be much more relevant to select patients at diagnosis. The authors answered: “Given that these cells were isolated from patients' blood and then cultured for 2–3 weeks with clonal selection and regular media changes, it is unlikely that any effects of the drugs would persist in culture”.

The reviewer would like to know whether the authors have any concrete clue that drugs cannot have any effects on cells. Actually, it is not known whether long-term exposition to vasodilators may affect the cells or not. Longitudinal studies should be performed to assert it. This should be better highlighted in the discussion.

Response: We do not have any data indicating whether the drug treatment affected ECFCs in culture. Importantly, regardless of the treatment, at the time of sampling, the patients still presented with PAH. We address this potential limitation in Discussion on page 15:

“…One limitation of this approach is that PAH patients were generally older and receiving treatment at the time of blood collection. To account for possible effects of age and medication, future work should consider obtaining cells at diagnosis or performing longitudinal studies to assess whether PAH therapies exert lasting effects on cultured ECFCs.…”

3. The reviewer is still puzzled by the elevated values of the mean PAP of treated patients. Are the authors sure that PAP values they show are mean PAP and not systolic PAP? Could the authors mention whether the PAP values are measured by right heart catheterization or transthoracic echocardiography? Can the authors provide other hemodynamic data such cardiac index, pulmonary vascular resistance? Could the authors mention how long the patients were treated by PAH drugs.

RESPONSE: We confirm that these mPAP data are indeed mPAP and were measured at right heart catheter. Mean cardiac index was 1.9 and mean PVR was 12.9 WU

Drug treatment was taken from the time of diagnosis to sampling time

This information has been included in the legend of Table M3 on page 41 of Supplement

4. The authors did not answer the question regarding the RNA quality. How the authors can be sure that the RNA is not degraded?

Response: We would like to clarify that the integrity of all RNA samples used in this study, including those used for RT-qPCR analyses and RNA-seq, was assessed in an Agilent TapeStation using High Sensitivity RNA ScreenTape (Agilent, cat no 5067-5579) and High Sensitivity RNA ScreenTape Ladder (Agilent, cat no 5067-5581). The RNA Integrity Number equivalent (RINe), a user-independent algorithm that automatically assesses RNA degradation, was calculated automatically using the Agilent TapeStation. All samples used in this study had RIN values of 8 or above, which indicates good sample integrity and low levels of degradation. To ensure the quality of the samples was kept high after QC, we only worked on surfaces cleaned with RNaseZap™ (Fisher Scientific, cat. no 10708345). Samples were manipulated wearing gloves and RNase-free filter tips. All reactions were carried out in RNase-free tubes (Life Technologies, cat. no AM12450). To further ensure maintenance of RNA sample integrity, samples were stored at -80˚C and thawed on ice prior to reverse transcription and NGS library preparation.

This information has now been added to Supplemental Methods on page 45

The authors mentioned that they used archived lung samples from MCT and SuHx rats. The authors should provide hemodynamics and Fulton index data from these rats to illustrate the severity of PH.

Response: We provide the haemodynamics and Fulton index data in the new Figure S19. The graphs are based on data published in "Arnold, N. D. et al. A therapeutic antibody targeting osteoprotegerin attenuates severe experimental pulmonary arterial hypertension. Nat Commun 10, 5183 (2019)[11].

We have also provided a brief description of experimental procedures in Supplemental Methods on page 60, based on [11]

6. The authors did not comment about the previous reviewer's question:" Expression of KLF6 mRNAs is much lower in MCT rats compared to hypoxic mice, whereas PH is known to be much more severe in MCT rats. In addition, remodelling of pulmonary arteries is much more prominent in MCT rats compared to hypoxic mice (see Figure 1C)". Can the authors comment on that and clarify that point?

Response: We have removed the mouse model together with the analysis of KLF6 mRNA levels in hypoxic mice and MCT rats from the manuscript. We have provided a semi-quantitative evaluation of KLF6 expression in MCT and SuHx rat lungs (New Figure S19B).

The total lung KLF6 mRNA levels did not appear to correlate with the degree of vascular remodelling. Differences in KLF6 mRNA expression in hypoxic mice compared with MCT-treated rats may result from different mechanisms of

hypoxia and MCT (the latter involving poorly understood mechanism of action and liver and lung toxicity[12]) and interspecies variation, so direct comparison between these two models and correlation of the level of KLF6 expression with the disease severity may not be possible to perform.

7. "The reviewer does not understand how the authors concluded that KLF6 is a key regulator of arterial identity". The answer of the authors is rather brief. Could the authors further elaborate on that?

Response: In the revised version of the manuscript (Revision 1), we did not describe KLF6 as a key regulator of arterial identity. Arterial identity was only mentioned in the context of SOX17.

SOX17 has been described as a regulator of arterial identity in Corada, M., Orsenigo, F., Morini, M. et al. "Sox17 is indispensable for acquisition and maintenance of arterial identity" Nat Commun 4, 2609 (2013)[13] and in [14]

We have made a small modification in the text on page 7, using a broader, and perhaps more accurate term describing SOX17 role as "a regulator of arterio-venous specification".

6. "Whether angiogenesis is beneficial or detrimental in the pathogenesis of PAH is under debate". The answer of the authors is rather brief. The authors should consider to better document the statement: "Inhibition of BMPR2 compromises angiogenic responses". Please see Gu 2017 Cell Stem Cell; Tielemans 2023 Pulm Circ; Spiekerkoetter 2013 J Clin Invest.

Response: Following the Reviewer's suggestions, we have expanded this part of Discussion on page 15

9. "The reviewer is not really convinced by the relevance of the findings regarding alveolar capillary dysplasia and wonder whether they should be included within the results." The authors answered that they would like to retain this part. The reviewer may understand but the authors should be more convincing and better elaborate on that.

Response: To better explain the reason for including ACD analysis, we have added the following text in Discussion on page 16:
"…To determine whether the molecular mechanisms identified may represent more general features of pulmonary vascular disease, we analysed single nucleus RNA sequencing data of samples from alveolar capillary dysplasia (ACD)[15,16]. Although ACD is distinct from PAH, the two conditions are characterised by hypoxemia and pulmonary hypertension and share several key pathological features, including the loss of capillaries, occlusive remodelling of the pulmonary arterial circulation and dysregulated angiogenesis[17]. Through this analysis, we identified an elevation in KLF6 expression within pulmonary arterial endothelial cells, suggesting that KLF6 may potentially act as an activator of

shared molecular pathways prominent in PAH and ACD[17], including vascular endothelial growth factor (VEGF)/VEGFR signalling, angiopoietin, hypoxia-inducible factor 1α, transforming growth factor-β, bone morphogenetic protein and NF-κB signalling.…"

10. The authors answered that "Predicting treatment outcomes might be too speculative, given that the role of angiogenesis in PAH is not fully understood". Could the authors consider that KLF6 may have a dual role in early stages versus advanced stages of the diseases? What about the discrepancies between MCT rats, SuHx rats and patients?

Response: We have expanded the final part of Discussion on pages 15 and 16. We argue that KLF6 activation may be beneficial, consistent with the reasoning that enhanced angiogenesis could be advantageous. We further speculate that pro-survival effects during the early stages of the disease may help prevent endothelial damage, whereas sustained or chronic activation could have detrimental consequences. We also consider the potential adverse effect of increased PASMC proliferation. As these hypotheses require experimental validation, we prefer to refrain from further speculation at this stage.

1 Ammann, K. R., DeCook, K. J., Li, M. & Slepian, M. J. Migration versus proliferation as contributor to in vitro wound healing of vascular endothelial and smooth muscle cells. *Exp Cell Res* **376**, 58-66 (2019). https://doi.org:10.1016/j.yexcr.2019.01.011

2 Muhleder, S., Fernandez-Chacon, M., Garcia-Gonzalez, I. & Benedito, R. Endothelial sprouting, proliferation, or senescence: tipping the balance from physiology to pathology. *Cell Mol Life Sci* **78**, 1329-1354 (2021). https://doi.org:10.1007/s00018-020-03664-y

3 Pontes-Quero, S. *et al.* High mitogenic stimulation arrests angiogenesis. *Nat Commun* **10**, 2016 (2019). https://doi.org:10.1038/s41467-019-09875-7

4 Shen, Y. H. *et al.* Panorama of artery endothelial cell dysfunction in pulmonary arterial hypertension. *J Mol Cell Cardiol* **197**, 61-77 (2024). https://doi.org:10.1016/j.yjmcc.2024.10.004

5 Liu, B. *et al.* General Capillary Endothelial Cells Undergo Reprogramming Into Arterial Endothelial Cells in Pulmonary Hypertension Through HIF-2alpha/Notch4 Pathway. *Circulation* **150**, 414-417 (2024). https://doi.org:10.1161/CIRCULATIONAHA.123.067981

6 Jonigk, D. *et al.* Plexiform lesions in pulmonary arterial hypertension composition, architecture, and microenvironment. *Am J Pathol* **179**, 167-179 (2011). https://doi.org:10.1016/j.ajpath.2011.03.040

7 Asosingh, K. *et al.* Single-cell transcriptomic profile of human pulmonary artery endothelial cells in health and pulmonary arterial hypertension. *Sci Rep* **11**, 14714 (2021). https://doi.org:10.1038/s41598-021-94163-y

8 Stek, C. *et al.* The Immune Mechanisms of Lung Parenchymal Damage in Tuberculosis and the Role of Host-Directed Therapy. *Front Microbiol* **9**, 2603 (2018). https://doi.org:10.3389/fmicb.2018.02603

9 Singh, E. & Bisserier, M. Experimental animal models and patient-derived platforms to bridge preclinical discovery and translational therapeutics in pulmonary arterial hypertension. *J Transl Med* **23**, 665 (2025). https://doi.org:10.1186/s12967-025-06709-7

10 Reynolds, A. M., Holmes, M. D., Danilov, S. M. & Reynolds, P. N. Targeted gene delivery of BMPR2 attenuates pulmonary hypertension. *Eur Respir J* **39**, 329-343 (2012). https://doi.org:10.1183/09031936.00187310

11 Arnold, N. D. *et al.* A therapeutic antibody targeting osteoprotegerin attenuates severe experimental pulmonary arterial hypertension. *Nat Commun* **10**, 5183 (2019). https://doi.org:10.1038/s41467-019-13139-9
12 Gomez-Arroyo, J. G. *et al.* The monocrotaline model of pulmonary hypertension in perspective. *Am J Physiol Lung Cell Mol Physiol* **302**, L363-369 (2012). https://doi.org:10.1152/ajplung.00212.2011
13 Corada, M. *et al.* Sox17 is indispensable for acquisition and maintenance of arterial identity. *Nat Commun* **4**, 2609 (2013). https://doi.org:10.1038/ncomms3609
14 Simmons Beck, R., Liang, O. D. & Klinger, J. R. Light at the ENDothelium-role of Sox17 and Runx1 in endothelial dysfunction and pulmonary arterial hypertension. *Front Cardiovasc Med* **10**, 1274033 (2023). https://doi.org:10.3389/fcvm.2023.1274033
15 Slot, E. *et al.* Alveolar capillary dysplasia with misalignment of the pulmonary veins: clinical, histological, and genetic aspects. *Pulm Circ* **8**, 2045894018795143 (2018). https://doi.org:10.1177/2045894018795143
16 Castilla-Fernandez, Y. *et al.* Alveolar capillary dysplasia with misalignment of pulmonary [corrected] veins: concordance between pathological and molecular diagnosis. *J Perinatol* **33**, 401-403 (2013). https://doi.org:10.1038/jp.2012.63
17 Kamp, J. C. *et al.* A Morphomolecular Approach to Alveolar Capillary Dysplasia. *Am J Pathol* **192**, 1110-1121 (2022). https://doi.org:10.1016/j.ajpath.2022.05.004
